# MoveTraits—A Database for Integrating Animal Behaviour Into Trait‐Based Ecology

**DOI:** 10.1111/ele.70297

**Published:** 2025-12-31

**Authors:** Larissa T. Beumer, Anne G. Hertel, Raphaël Royauté, Marlee A. Tucker, Jörg Albrecht, Roxanne S. Beltran, Francesca Cagnacci, Sarah C. Davidson, Nandintsetseg Dejid, Roland Kays, Andrea Kölzsch, Ashley Lohr, Eike Lena Neuschulz, Kamran Safi, Anne K. Scharf, Matthias Schleuning, Martin Wikelski, Thomas Mueller

**Affiliations:** ^1^ Senckenberg Biodiversity and Climate Research Centre (SBiK‐F) Frankfurt am Main Germany; ^2^ Department of Arctic Biology The University Centre in Svalbard Longyearbyen Norway; ^3^ Behavioural Ecology, Department of Biology Ludwig‐Maximilians University of Munich Planegg‐Martinsried Germany; ^4^ Université Paris‐Saclay, INRAE, AgroParisTech, UMR EcoSys Palaiseau France; ^5^ Department of Environmental Science, Radboud Institute for Biological and Environmental Sciences Radboud University Nijmegen the Netherlands; ^6^ Department of Ecology and Evolutionary Biology University of California Santa Cruz Santa Cruz California USA; ^7^ Animal Ecology Unit, Research and Innovation Centre Edmund Mach Foundation Trento Italy; ^8^ Department of Migration Max Planck Institute of Animal Behavior Radolfzell Germany; ^9^ Department of Biology University of Konstanz Constance Germany; ^10^ North Carolina Museum of Natural Sciences Raleigh North Carolina USA; ^11^ Department of Forestry and Environmental Resources North Carolina State University Raleigh North Carolina USA; ^12^ Ecology Department, Radboud Institute for Biological and Environmental Sciences Radboud University Nijmegen the Netherlands; ^13^ Department of Biology Philipps‐Universität Marburg Marburg Germany; ^14^ Department of Biological Sciences Goethe University Frankfurt am Main Germany

**Keywords:** biodiversity, biologging, Essential Biodiversity Variables, FAIR data, global change, interoperability, macroecology, movement ecology, repository, trait data

## Abstract

Trait‐based approaches are key to understanding eco‐evolutionary processes but rarely account for animal behaviour despite its central role in ecosystem dynamics. We propose integrating behaviour into trait‐based ecology through movement traits—standardised and comparable measures of animal movement derived from biologging data, such as daily displacements or range sizes. Accounting for animal behaviour will advance trait‐based research on species interactions, community structure and ecosystem functioning. Importantly, movement traits allow for quantification of behavioural reaction norms, offering insights into species’ acclimation and adaptive capacity to environmental change. We outline a vision for a ‘living’ global movement trait database that enhances trait data curation by (1) continuously growing alongside shared biologging data, (2) calculating traits directly from individual‐level data using standardised, consistent methodology and (3) providing information on multi‐level (species, individual, within‐individual) trait variation. We present a proof‐of‐concept ‘MoveTraits’ database with 52 mammal and 97 bird species, demonstrating calculation workflows for 5 traits across multiple timescales. Movement traits have significant potential to improve trait‐based global change predictions and contribute to global biodiversity assessments as Essential Biodiversity Variables. By making animal movement data more accessible and interpretable, this database could bridge the gap between movement ecology and biodiversity policy, facilitating evidence‐based conservation.

## Graphical abstract

We propose integrating animal behaviour into trait‐based ecology through movement traits – standardised and comparable metrics of animal movement derived from biologging data, such as daily displacements or range sizes. Explicitly accounting for behaviour will advance trait‐based research and allow for the quantification of behavioural reaction norms, offering insights into species' adaptive capacity to environmental change. We provide a first proof‐of‐concept ‘MoveTraits’ database with standardised, multi‐scale (species, individual, within‐individual) movement traits for 52 mammal and 97 bird species across more than 5000 individuals.

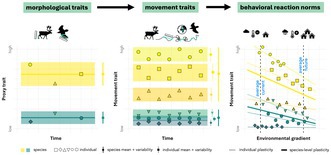

## 1 Introduction

Identifying the mechanisms underpinning organismal interactions is key to move from a simple description of observed patterns to predicting ecological and evolutionary processes (Funk et al. 2017). In this context, trait‐based approaches have emerged as a promising tool, driving rapid progress over the last two decades (Green et al. 2022; McGill et al. 2006; Violle et al. 2007). Instead of solely focusing on species identity, trait‐based ecology posits that an organism's ecological role and distribution in space and time can be understood through the combination and interaction of a suite of measurable traits (Lavorel and Garnier 2002; McGill et al. 2006). Recognising that community and ecosystem functions ultimately arise from the collective interactions of individual organisms, an emphasis on traits boosts our ability to identify generalisable principles governing eco‐evolutionary processes across scales, for instance by facilitating macro‐ecological and macro‐evolutionary studies (Funk et al. 2017; Harfoot et al. 2014; Lavorel and Garnier 2002).

Initially, trait‐based concepts, data collections, and insights into community assembly and ecosystem functioning focused almost entirely on plant traits (Kattge et al. 2020; Lavorel and Garnier 2002; McGill et al. 2006; Suding et al. 2008). Given animals' crucial role in ecosystem functioning, especially in linking trophic levels (Schleuning et al. 2023), attention has recently turned to animal functional traits, leading to the curation of global‐scale trait databases for various animal taxa, for example PanTHERIA (Jones et al. 2009), EltonTraits (Wilman et al. 2014), Amniote (Myhrvold et al. 2015), the SPI‐Birds data hub (Culina et al. 2021), AnimalTraits (Herberstein et al. 2022), COMBINE (Soria et al. 2021), AVONET (Tobias et al. 2022) and FuTRES (Balk et al. 2022). These databases fill an important data gap to facilitate the integration of animal and plant trait data, critical for a broader understanding of plant–animal interactions as a fundamental pillar of ecological communities. While these global animal‐trait databases have been influential (e.g., Newbold et al. 2015; Olival et al. 2017; Rigal et al. 2023; Tucker et al. 2023), they remain mostly restricted to morphological traits (e.g., body or brain size) or general ecological traits (e.g., trophic guild, diet), which are relatively stable between and within individuals and are typically sourced from the literature and museum collections (Balk et al. 2022; Schleuning et al. 2023) (Box 1).

BOX 1 **Glossary.**

**Trait**: A well‐defined, measurable property of organisms, usually measured at the individual level and used comparatively across species (Dawson et al. 2021; McGill et al. 2006). Common categories are morphological, physiological, phenological, life history or behavioural traits; see Figure 2.

**Functional trait**: Traits that influence the organism's performance under different environmental conditions and/or its effect on ecosystem processes (e.g., seed dispersal, nutrient cycling) (Dawson et al. 2021; McGill et al. 2006; but see Sobral 2021).

**Trait‐based ecology**: An ecological discipline, focusing on the role of traits in shaping ecological interactions, species distribution and abundance, patterns of biodiversity and ecosystem functioning (Garnier et al. 2015; Green et al. 2022). Compared to species‐based approaches which focus on the identity of a given organism, trait‐based approaches use traits to derive information about an organism's performance, its functions and the functions of higher levels of organisation such as populations, communities and ecosystems (Zakharova et al. 2019).

**Animal biologging**: The use of miniaturised animal‐attached tags (‘biologgers’) for recording data about an animal's movements, behaviour, physiology and/or environment (Rutz and Hays 2009). Traditionally, biologging (tags logging data in memory) is distinguished from biotelemetry (tags transmitting data to a receiver or satellite) (Watanabe and Papastamatiou 2023). However, due to the emergence of hybrid devices and the increasing complementary use of both device types, this distinction is increasingly fading in practice. Here, we collectively refer to both approaches as biologging.

**Animal movement**: In movement ecology, animal movement is typically defined as the change in the spatial location of an individual in time (Nathan et al. 2008). It is a fundamental aspect of animal behaviour, operating across multiple scales—from fine‐scale local movements to long‐distance migrations. With animal biologging, additional dimensions of movement can be quantified, such as wing flaps, head movements or lying versus standing. For this article, we focus on animal movements as location changes in geographic space.

**Movement metric**: A quantitative measure to describe and analyse the patterns of animal movement in space and time, inferred from biologgers. Typically, metrics either characterise an animal's path (focussing on the one‐dimensional aspects of movement trajectories, such as displacement or speed) or space use (focussing on the two‐dimensional patterns, incl. home range size or utilisation distribution).

**Movement trait**: An agreed‐upon movement metric that is calculated at the individual level and in a comparable and standardised way across many taxa and therefore complies with operational trait definitions. We consider movement traits to be a subcategory of behavioural traits (see Figure 2).

One key aspect that remains underrepresented in trait‐based ecology is animal behaviour, despite its recognised importance for many ecological processes ranging from individuals' fitness to ecosystem‐level functions (e.g., Nagelkerken and Munday 2016; Rahman and Candolin 2022; Réale et al. 2007; Wilson et al. 2020). This is a critical oversight, especially in the context of global change studies (Buchholz et al. 2019; Marske et al. 2023), where behavioural responses can significantly mediate species' abilities to cope with changing environments (see, e.g., Hall and Chalfoun 2019; Mason et al. 2014; Mathewson et al. 2017). Evidence of human impacts on animal behaviour (e.g., Suraci et al. 2019; Tucker et al. 2018; Wong and Candolin 2015), and animals' behavioural adjustments to environmental change (e.g., Abrahms et al. 2018; Johansson et al. 2024; Wong and Candolin 2015) is abundant. Such behavioural changes can trigger cascading effects on population dynamics, species interactions, community structure and ecosystem function (Marske et al. 2023; Wilson et al. 2020). Yet, these insights are often not incorporated into broader trait‐based analyses. A primary impediment to integrating behaviour is the limited availability of standardised and comparable behavioural trait data collected from wild animals (Box 2).

BOX 2 **Trait‐based approaches in behavioural ecology.**

Behavioural ecology aims to understand the ecological and evolutionary basis of animal behaviour, and—like trait‐based approaches in general—concentrates on the individual as the focal unit of analysis (Carter et al. 2013). While in trait‐based ecology, traits are often used to capture differences between species, in behavioural ecology, the use of traits has mainly focused on behavioural differences between individuals of the same species that are repeatable over time and across situations (i.e., animal temperament or personality, Réale et al. 2007). Analogue to the ‘big 5’ temperament traits in human psychological research, initial work focused on quantifying individuals’ aggressiveness, boldness, activity, exploration, and sociability (Réale et al. 2007), using experimental tests in the lab or controlled setups in the wild (Carter et al. 2013). While standardised test setups control for environmental confounds, they are impractical to quantify behavioural differences across a wide range of species, causing a gap of comparable behavioural trait studies in the wild. More recently, an alternative approach has emerged to study behavioural traits, purely based on statistical partitioning of behavioural variation into its environmental, among‐ and within‐individual sources (Dall and Griffith 2014; Dingemanse et al. 2010; Dingemanse and Dochtermann 2013). This ‘statistical’ approach is not limited to the five major personality traits but is instead founded on the notion that individuals can differ in their average expression of any kind of behaviour (Dall and Griffith 2014; Dingemanse et al. 2010). Between‐individual behavioural variation is quantified by repeatedly measuring individual behaviour across different biological contexts (Dingemanse et al. 2010). Using this approach, intrinsic individual variation (i.e., an individual's average behaviour) can be discerned from reversible behavioural plasticity. Because behaviour is at least partially heritable (Dochtermann et al. 2019), between‐individual behavioural variation is the substrate for natural selection and can speed up adaptation to environmental change (Réale et al. 2007; Wolf and Weissing 2012).

A key facet of animal behaviour that can be quantified for wild animals is movement. Animal movements (see Glossary) represent the way animals interact with their biotic and abiotic environment. Their influence on individuals' survival and reproduction, as well as ecological interactions within and across trophic levels, make them ecologically and evolutionarily relevant (Cagnacci et al. 2010; Jeltsch et al. 2013). Using bio‐logging technologies, movement can be recorded in situ for an ever‐growing number of species and individuals (Hussey et al. 2015; Kays et al. 2015; Nathan et al. 2022). However, even though a wealth of animal movement data is available and partly harmonised in shared databases (Davidson et al. 2025; Harcourt et al. 2019), animal movement is still rarely directly integrated into trait‐based approaches. This is due to the fact that the billions of animal location records cannot be readily used as traits in their initial form but need to be processed into informative metrics first. In the past, comparative work mostly relied on sourcing such processed movement metrics from the literature (Tucker et al. 2014, but see e.g., Huang et al. 2021). A major shortcoming of this approach is that these estimates are derived using varying methodologies and calculated at different levels (e.g., individual versus species). In addition, they are based on data collected with varying sampling intervals, which may introduce error and bias as most methods for deriving metrics from animal movement data are sensitive to sampling rates (Calabrese et al. 2016). Therefore, a significant obstacle that remains is the lack of a comprehensive database assembling standardised movement metrics that represent comparable movement traits (see Glossary). This step is essential for animal movement data to be usable in trait‐based approaches.

**FIGURE 1:**
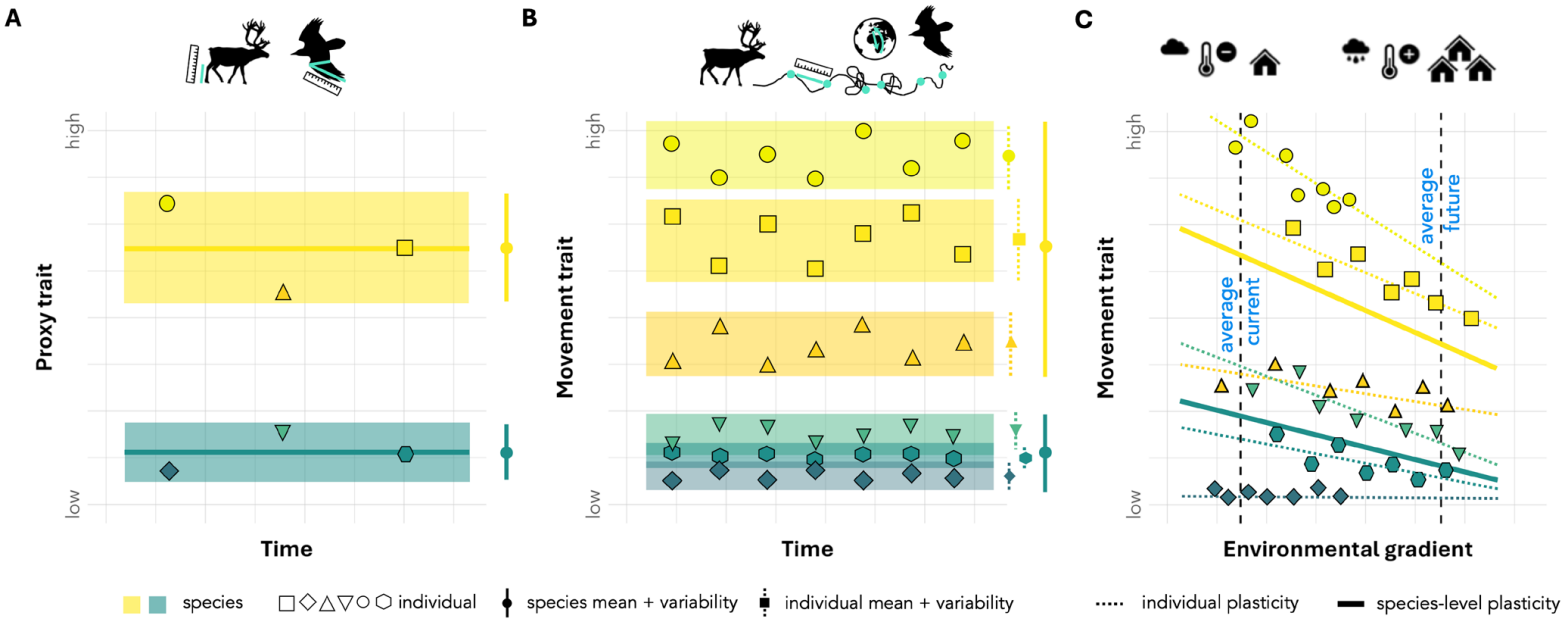
Conceptual advances provided by movement traits. (A) In trait‐based ecological studies, animal movement behaviour is often incorporated via ‘proxy traits’—typically morphological and ecological traits, such as body size/mass, wing morphology or trophic guild, which are typically derived from museum collections and measured only once per individual. This technically allows for quantification of within‐species trait variation, although most trait databases typically only contain trait values averaged at the species level, often without reporting the underlying trait measurements, variance measures, or sample sizes. (B) Biologging devices measure behavioural information repeatedly for the same individual in its natural environment and over ecologically meaningful time periods. Building on these repeated individual‐level raw data, movement traits can be calculated in a standardised workflow with consistent methodology, achieving (1) more comparable measures including variance estimates and sample sizes, and (2) quantification of both between‐species, between‐individual and within‐individual trait variation. (C) Finally, animals may display considerable plasticity in behavioural reactions to their encountered abiotic and biotic environment. Individual measures of movement traits along environmental gradients facilitate incorporation of *behavioural reaction norms*, combining consistent between‐individual differences in behaviour independent of context (different intercepts) with within‐individual behavioural plasticity (individuals adjusting behaviour adaptively to changing conditions; non‐zero slopes). Accounting for behavioural reaction norms should enable better predictions of community dynamics and ecosystem processes under global change. Panel C adapted from Hertel et al. (2020). Animal silhouettes from PhyloPic.org: *Rangifer tarandus* by T. Michael Keesey, *Corvus corax* by Andy Wilson (both CC0 1.0 Universal)

Given the lack of standardised and comparable movement trait data, the relevance of animal mobility to ecosystem dynamics is often indirectly acknowledged in trait‐based studies by incorporating animal movement behaviour via other types of traits. These are usually morphological or general ecological traits (hereafter referred to as ‘proxy traits’), available from the recently developed animal‐focussed trait databases (Figure 1). For example, animals' capacity to disperse seeds is commonly approximated via body size or mass due to a suggested allometric scaling of dispersal movements (Jenkins et al. 2007; Sorensen et al. 2020; Stevens et al. 2014) and daily distance travelled (Carbone et al. 2005). While proxy traits may represent simple categorisations of or scale allometrically with species' average movement behaviour (Sheard et al. 2020; Tucker et al. 2014), they may overlook critical movement variation by (1) discounting behavioural variation between species with similar proxy traits, (2) not adequately reflecting behavioural variation within species, (3) disregarding spatio‐temporal patterns of animal movement and behaviour and/or (4) not accounting for animals' behavioural plasticity in responding to fluctuating biotic and abiotic conditions (Figure 1). Hence, while proxy traits might explain some patterns (e.g., average home range size across mammals with large differences in body size; Tucker et al. 2014) and approximate a species’ movement ‘potential’, they may omit key information on the variability and context‐dependence of trait effects that are critical to improve our understanding of species interactions, community structure and ecosystem functioning.

To overcome these limitations, we outline a vision for the development of a movement trait repository as a ‘living’ database, continuously updated and growing alongside biologging databases. We suggest that such a database would provide a new approach for curating trait databases by (1) directly building on individual‐level data and (2) facilitating trait calculation in a standardised workflow with consistent methodology. In addition, we propose a way to (3) provide information on multi‐level (species, between‐individual, within‐individual) trait variation thus far lacking from existing trait databases. We believe that this approach will achieve more comparable measures and therefore more generality in the inferred ecological insight, which is crucial for effective biodiversity conservation and environmental management. Demonstrating the practical workflow from biologging data to trait calculation, we present a first proof‐of‐concept animal movement trait dataset based on standardised analysis of biologging data and illustrate how these movement traits can fill critical trait data gaps. We conclude by discussing how such a movement trait database will unlock the potential of biologging data to integrate animal behaviour into trait‐based ecology, global change prediction, environmental monitoring, and global biodiversity assessments, with the potential to inform Essential Biodiversity Variables (EBVs) and bridge the gap between movement ecology research and biodiversity policy.

## 2 Conceptual Alignment: Animal Movement Reflects Key Behavioural Traits

**FIGURE 2:**
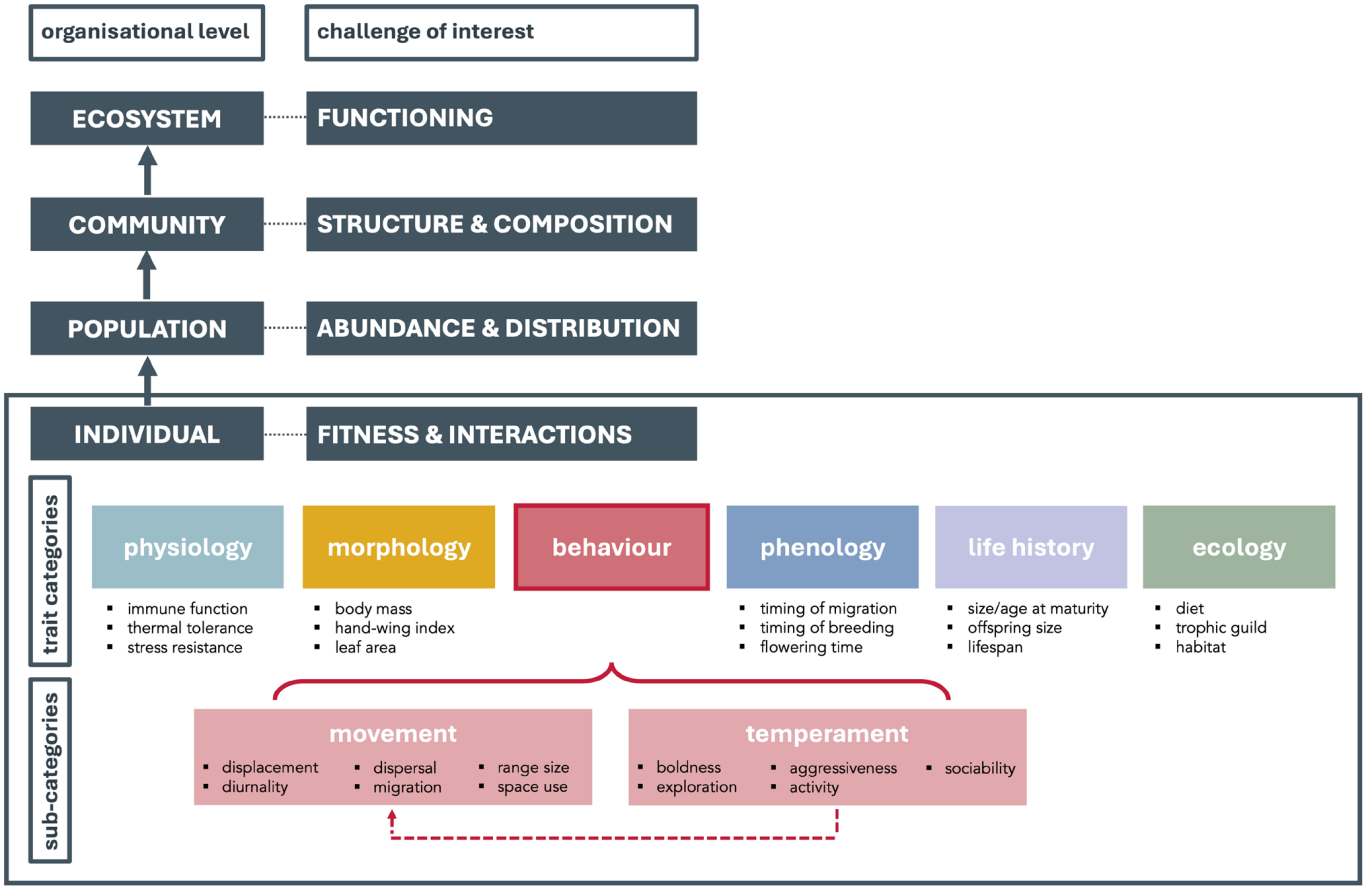
Trait‐based approaches have been used in various ecological disciplines, leading to a plethora of trait concepts, definitions, and categories (Violle et al. 2007). Here, we suggest a ‘taxonomy of traits’ with movement traits as a subcategory of behavioural traits, as movement always reflects behaviour, but not all behaviour is expressed via movement. However, we note that there can be considerable overlap between trait categories and suggest a pragmatic approach to such classifications.

Traits are morphological, physiological, phenological, life history or behavioural properties of organisms (Figure 2), measured at the individual level and often averaged per species for comparative cross‐species studies (Dawson et al. 2021; McGill et al. 2006; see Glossary). The term ‘functional trait’ refers to traits that influence the organism's performance under different environmental conditions (Dawson et al. 2021; Garnier et al. 2015) and/or its effect on ecosystem processes (e.g., seed dispersal, nutrient cycling) (Dawson et al. 2021; McGill et al. 2006; but see Sobral 2021). Although traits are defined without reference to the environment, measured trait *values* need to be associated with spatially and temporally explicit environmental conditions (annotated) to facilitate the interpretation of ecological and evolutionary significance (Dawson et al. 2021; Garnier et al. 2015). Generally, traits, including behavioural traits, are assumed to be heritable to some degree, and thus under natural selection (Dochtermann et al. 2019, see Box 2).

Metrics inferred from animal movement data can comply with these commonly used operational definitions of traits and have the potential to be useful in comparative approaches (see Glossary): Biologging‐derived animal movement data are always collected at the individual level and usually recorded automatically and repeatedly at regular time intervals and without observer bias (Figure 1). The inferred metrics are, in principle, precisely defined and clearly measurable. However, only those movement metrics that can be calculated in a standardised way and that can be averaged and compared across a number of species (Joly et al. 2019; Tucker et al. 2018) can serve as useful movement traits. As the recorded locations are spatio‐temporally explicit, their annotation with environmental context is feasible. Recent studies demonstrate a genetic basis (i.e., heritability) for movement traits such as migration direction or habitat preferences (Bonar et al. 2022; Gervais et al. 2020), indicating the potential for evolution and adaptive responses to selective pressures. Given the importance of animal mobility for individual fitness and ecological processes, most movement traits would qualify as functional traits: Animal movement is a critical determinant of individual fitness, for instance by determining success in resource acquisition and risk avoidance (DeMars and Boutin 2018; Gaillard et al. 2010; McLoughlin et al. 2006; Mitchell and Lima 2002). Likewise, it underpins key ecological processes of importance for (meta‐)community dynamics and ecosystem functioning (Nathan et al. 2008; Schlägel et al. 2020). These include, for instance, seed dispersal (Graf et al. 2024; Kays et al. 2011), nutrient transfer (Doughty et al. 2016; McLoughlin et al. 2016), disease dynamics (Manlove et al. 2022; Scherer et al. 2020), spread of invasive species (Reynolds et al. 2015) and trophic interactions (DeMars and Boutin 2018; Mitchell and Lima 2002). Consequently, animal movement provides critical ecosystem services and Nature Contributions to People (Kremen et al. 2007).

Given this conceptual alignment, we posit that movement data are an under‐used source of trait information, that is, can serve as movement traits to quantify animal behaviour for integration into trait‐based ecology. We suggest that such movement traits are a subcategory of behavioural traits (Figure 2), as movement is always a reflection of behaviour (e.g., foraging, territoriality, exploration) but not all behaviour is necessarily expressed via movements that result in a change of the individuals' spatial location (e.g., sociability or aggressiveness expressed through facial expressions or other body language). We note, however, that there is considerable overlap between some trait categories. For instance, while migration distance is a movement trait (Figure 2), the timing of migration is a phenological trait. Similarly, sprint performance or stamina would be a physiological, rather than a movement trait per se. We suggest a pragmatic approach to such trait classifications.

A particular property of behavioural traits, including movement, is that they are labile traits, meaning that their expression may change from measurement to measurement (Blomberg et al. 2003; Scheiner 1993). Morphological traits, like structural size, tarsus or wing length, are usually more static both between and within individuals outside of ontogeny or organism growth periods. This property has several implications for trait measurements and the eco‐evolutionary insights they afford: (1) Behavioural traits should always be based on repeated measures to approximate their centrality (e.g., mean or median) and should be accompanied by measures of variability (e.g., variance or standard deviation) (Niemelä and Dingemanse 2017). (2) If repeated individual measures are recorded, they also allow for the partitioning of behavioural variability into between‐individual and environmental sources (Dingemanse and Dochtermann 2013). Between‐individual variation in movement traits is generally high (Stuber et al. 2022) and enables natural selection on existing trait variation, facilitating faster adaptation to environmental change as compared to evolution via genetic mutation (Barrett and Schluter 2008; Wolf and Weissing 2012). Species with greater heritable between‐individual variation therefore likely have a greater *adaptive capacity* to fast‐paced environmental change. (3) Animals are able to adjust behaviour more readily than morphology to their environmental context, and this trait lability can support individual‐level acclimation via reversible behavioural plasticity (Beever et al. 2017; Dingemanse et al. 2010). Repeated measurements of individual‐level labile traits like movement allow the quantification of changes in behavioural expression along biotic or abiotic environmental gradients, a concept called ‘behavioural reaction norm’ (Figure 1; Dingemanse et al. 2010). Importantly, individuals can differ in their plasticity (i.e., their reaction norm slope) to experienced environmental gradients—a scenario under which plasticity can be selected and hence evolve. The ability to quantify between‐individual variation in plasticity is therefore likewise critical to predict a population's adaptive capacity to environmental change by offering a lens into the *acclimation potential* of the species to environmental shifts outside the currently experienced range (Johansson et al. 2024; Nussey et al. 2007). As biologging devices measure behavioural information repeatedly for the same individual in its natural environment and over ecologically meaningful time periods (Figure 1; Hertel et al. 2020), movement traits thus provide a powerful opportunity for trait‐based research to investigate the adaptive capacity and acclimation potential of different populations or species to environmental change via (a) the amount of between‐individual trait variability, and (b) the degree of between‐individual variation in behavioural plasticity to environmental change (Hertel et al. 2020, 2021).

## 3 MoveTraits v 0.1—A First Proof‐of‐Concept Movement Trait Database

To demonstrate the practical implementation of movement trait calculation, we provide a first proof‐of‐concept movement trait database (MoveTraits v 0.1) derived from movement data harmonised and publicly available on Movebank (https://www.movebank.org/; 212 studies, effective March 2025, see Table S3) and open access data published by Tucker et al. (2023) (46 studies, Table S3). We focused on GPS tracking data due to their spatial accuracy as well as temporal regularity and resolution. This initial version of the MoveTraits database includes movement traits from 97 bird and 52 mammal species, represented by 5873 individuals (3294 birds, 2579 mammals).

Animal movement trajectories can be partitioned temporally at scales of steps, daily and seasonal routes, life‐history level (e.g., home range), or even lifetime tracks (Kays et al. 2015; Nathan et al. 2008). To this end, a variety of movement metrics have been developed, facilitating direct comparisons of movements across individuals, populations and species. For example, Abrahms et al. (2017) identified several movement metrics useful for classifying broad‐scale movement patterns for large marine and terrestrial vertebrate species (e.g., migratory versus range‐resident). Similarly, metrics deployed by Tucker et al. (2018) proved insightful in determining movement responses to human activity across terrestrial mammal species, and Joly et al. (2019) provided simple, comparable and repeatable metrics for quantifying annual migratory movement distances for terrestrial mammals. For our first proof‐of‐concept version of MoveTraits, we decided on an initial suite of simple movement traits (Table 1) that we consider to (1) provide complementary insights on animals' movement behaviour, (2) be relevant and comparable across a diverse range of taxa, movement modes and environmental contexts and (3) be relatively straightforward to infer from commonly collected animal movement data. This suite is not meant to be a static final set, does not apply to all taxonomic groups and we hope will be expanded in the future.

**TABLE 1:** Initial suite of movement trait metrics estimated at the individual level and for different time scales. Metrics were calculated and summarised at the individual level using the entire tracking period of that individual (excluding data gaps) assuming that enough data were recorded to estimate the traits' centrality and uncertainty (Table S1).

Movement trait	Time scales	Definition/Calculation	Ecological significance
Displacement distance [m]	Hourly (1 h), daily (24 h)	Straight‐line Euclidean distance between subsequent locations	Realised movement distances at different time scales
Maximum displacement distance [m]	Daily (24 h), weekly (7 day)	Maximum distance observed across all pairwise distances in a given period	Long‐distance movement capacity
Range size [km^2^]	Daily, weekly, monthly, annual	95% minimum convex polygon (MCP)	Overall space requirements
Intensity of area use	Daily, monthly	Ratio between total movement distance (cumulative sum of consecutive distances), divided by the square root of the range size (95% MCP)	Summarising linear and area‐based extent of movement; lower values indicate more straight movements (e.g., migrations), higher values more clustered movements
Diurnality index [−1 to 1]	Entire tracking period	The relative activity during the day conditional on day length	Indicates whether movement occurs primarily during the day (1) or night (−1)

First, we resampled the GPS data (available from open‐access Movebank studies and Tucker et al. (2023), see above) to regular time intervals of 1 h (‘hourly’), 24 h (‘daily’) and 7 days (‘weekly’). Then, we used the resampled data to derive movement metrics at different timescales: We calculated hourly and daily step length, that is, the straight‐line distance between locations. We also quantified daily, weekly and annual maximum displacement distances as the maximum of all pairwise (i.e., not consecutive) distances within the sampling period. To summarise individuals’ space requirements, we delineated range size from the 95% Minimum Convex Polygon of all GPS locations at a daily, weekly, monthly and annual timescale. In addition, we also calculated Intensity of Use, a metric that summarises the spread of movement from more linear to more clustered movement patterns (Almeida et al. 2010). Finally, we calculated a diurnality index, indicating whether movement (based on hourly steps) occurs mostly during the local night or day (Hoogenboom et al. 1984). For more details on data requirements and trait calculations, see Table S1 and Supplementary Methods S1.

MoveTraits v 0.1 provides the movement trait data at three hierarchical levels (Figures 1 and 4; Box 3): (1) summarised at the *species level*, facilitating interoperability with other species‐level trait databases, (2) summarised at the *individual level* for studies on between‐individual variation and (3) the underlying repeated movement trait estimates for each individual over time to allow for research questions at the *within‐individual level*. We summarised the underlying repeated movement trait estimates at the individual level as mean, median, coefficient of variation and 5th as well as 95th percentile and provided species means of centrality and variance (e.g., the mean species 95th percentile summarised from individual 95th percentiles for a given trait), facilitating different ecological inquiries. For example, the mean daily displacement distance may signify an individual's daily routine movements, while the 95th percentile represents an individual's occasional daily long‐distance movements. Each metric was only estimated for individuals with sufficient repeated measures to allow for meaningful measures of centrality and variance (Table S1). For instance, we only summarised daily displacement distance (Table 1) for individuals with at least 30 days of data. Therefore, not all traits are calculated for every individual. We annotated each individual's trait summary with mean coordinates (to allow spatial annotation with environmental data), tracking period (length, start and end date; for temporal annotation with environmental data), species name and common name, taxonomic class (mammal, bird), movement mode (walk, fly, swim), individual information such as sex, age and body mass (if available) and details of the data owner (see also Table S2).

BOX 3 **Examples of mammal movement traits at the species, individual and within‐individual level.**

**FIGURE 3:**
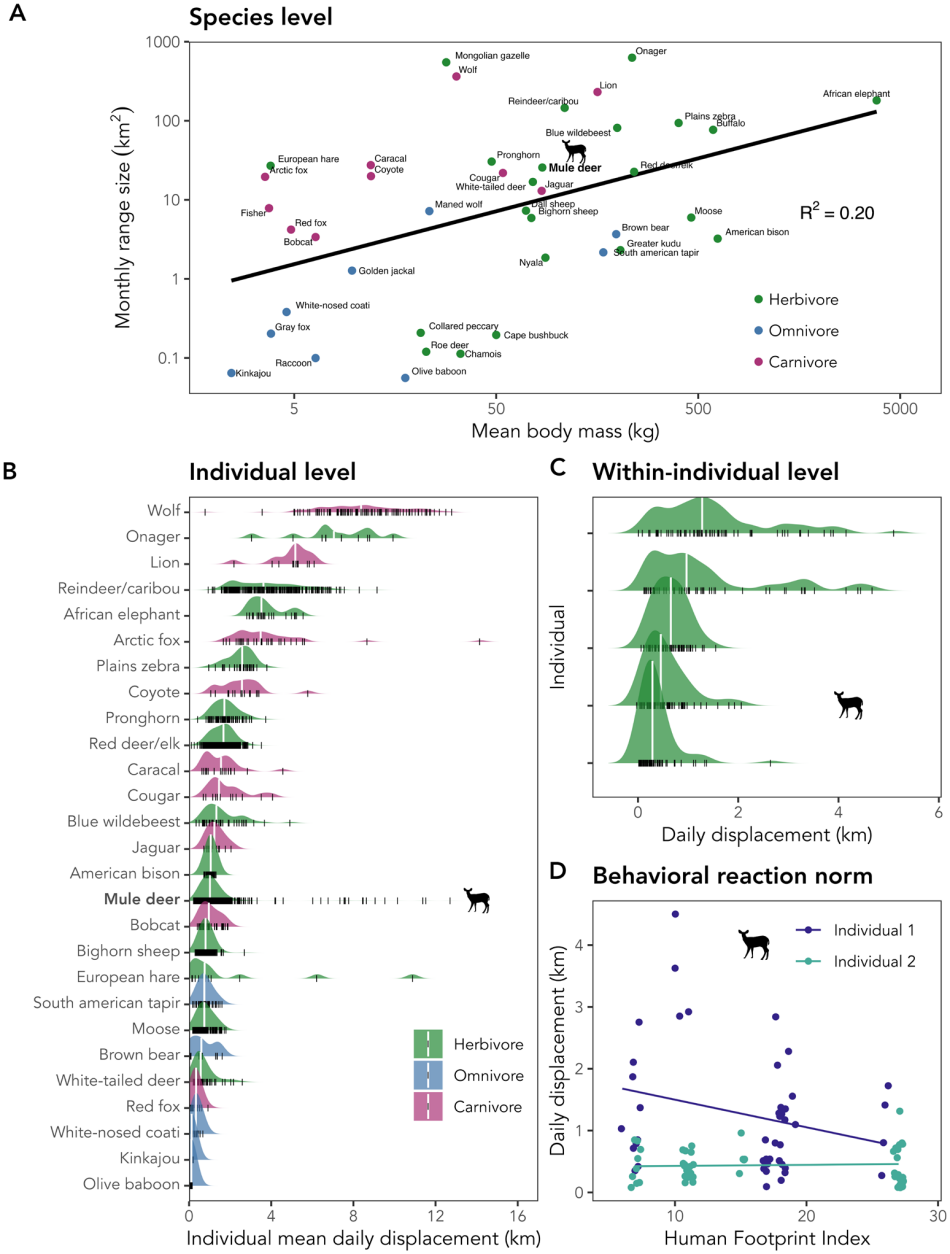
Examples showcasing the utility of movement traits at the species, individual, and within‐individual level, extracted from our first proof‐of‐concept MoveTraits database. (A) Log‐transformed species mean body mass against log‐transformed monthly range size for 41 terrestrial mammal species for which monthly range size could be calculated. (B) Between‐individual variation in mean daily displacement distance for 27 mammal species for which daily displacements were available. Individual means are indicated with black lines and their overall distribution at the species level with density ridges, with species means indicated by white vertical lines. (C) Within‐individual variation in daily displacement distances of five non‐migratory mule deer ( *Odocoileus hemionus* ). (D) Illustrative example for a behavioural reaction norm: Daily displacement distances of two non‐migratory mule deer ( *Odocoileus hemionus* ) measured along a gradient of human disturbance in the central United States (Utah & Wyoming), quantified by the Human Footprint Index. A significant interaction between individuals and HFI (*F*(1, 2229679) = 4.863, *p* = 0.029) suggests that the two individuals adjust movement differently to HFI. Data for panel C and D were collected between March and May 2019 by Matthew Kauffman and Julie Young and were sourced from the Tucker et al. (2023) open‐access dataset. Silhouette of *Odocoileus hemionus* by Gabriela Palomo‐Munoz, from PhyloPic.org, licensed under CC BY‐NC 3.0 (https://creativecommons.org/licenses/by‐nc/3.0/).

To showcase the utility of movement traits summarised at the three hierarchical levels, we present three examples (Figure 3).

Example 1 (species level): Morphological traits are commonly used as proxies to account for species movement capacity in trait‐based studies. Animal movements, such as home range size or maximum dispersal distances (Stevens et al. 2014), have been shown to allometrically scale with body mass in terrestrial animals. For example, body size alone has been reported to explain 52% of the variance in home range size of terrestrial mammals (Tucker et al. 2014). Similarly, the hand‐wing index, a metric of bird wing morphology linked to the wing aspect ratio, has been shown to correlate with flight efficiency, dispersal distance and long‐distance movements in birds (Lockwood et al. 1998; Sheard et al. 2020). To test how closely empirically derived movement traits relate to proxy traits, we linked the species‐mean range size trait to the species‐mean body mass trait from the PanTHERIA mammal trait database, for all terrestrial mammal species with at least one individual with 14 days of daily location data (*n* = 41; Table S1). While the general positive relationship between body mass and range size holds, it explains limited variance (*R* ^2^ = 0.20), and substantial uncertainty remains around the trend line (Figure 3A, see also Figure S1 for a correlation between hand‐wing index and flight distance). Accounting for movement capacity directly, when possible, instead of via proxy traits will thus allow refinement of trait‐based studies.

Example 2 (individual level): Trait‐based studies traditionally rely on species‐averaged traits. However, between‐individual variation can be substantial and is typically not accounted for. Importantly, between‐individual variation may not be the same across species and is generally expected to increase with the trait mean. Using daily displacement distances summarised at the individual level from terrestrial mammal species with at least 10 unique individuals (*n* = 27; Table S1), we find great variation among species in their between‐individual variance (Figure 3B). These findings imply that species averages may reflect the movement capacity of some species (e.g., moose, Figure 3B), while for others, these averages do not capture the breadth of behavioural diversity (e.g., blue wildebeest or mule deer, Figure 3B,C). Disregarding between‐individual variance may therefore impede correct ecological inference.

Example 3 (within‐individual level): As movement data are collected at the individual level and repeatedly through time, they facilitate the observation of individual phenotypic expression of movement traits in response to the environment, that is, individual phenotypic plasticity. The reaction norm framework enables the quantification of between‐individual variation in phenotypic plasticity via random slopes. We here highlight the movement responses of two non‐migratory mule deer along a gradient of human disturbance in the western United States (Utah and Wyoming), measured as Human Footprint Index (Figure 3D). The mean daily location information that is provided at the within‐individual level for repeated measurements of the trait ‘daily displacement distance’ allows for the annotation of movement traits with environmental covariates. In our example, the two individuals vary in their baseline daily displacement distance in undisturbed landscapes (420 m vs. 1600 m at HFI 6) as well as in their responsiveness to human disturbance, with one individual decreasing daily displacements while the other one does not adjust displacements. While reductions in mammalian movements to human disturbance have been shown globally (Tucker et al. 2018), our example demonstrates the importance and potential of studying individual responses via repeated trait measures.

See Figure S1 for corresponding relationships for bird species.

This multi‐level approach is novel: most trait databases only contain trait values averaged at the species level (but see Balk et al. 2022), reported without the underlying individual‐level trait measurements, variance measures or sample sizes (Balk et al. 2022; Beltran et al. 2025; Moran et al. 2016). Therefore, information critical for interpreting species‐level trait values is usually missing, complicating error tracing, preventing discoverability, replicability and interoperability of trait data and limiting ecological insight (Balk et al. 2022; Bartomeus et al. 2016; Moran et al. 2016). Our multi‐scale approach explicitly avoids such a reduction to species‐mean values, while still facilitating easy interoperability with existing species‐mean trait databases. The individual‐level trait data and metadata ensure user flexibility, enabling aggregation to any desired organisational level (e.g., population).

## 4 Towards a Global ‘Living’ MoveTraits Database

### 4.1 Envisioned Implementation

**FIGURE 4:**
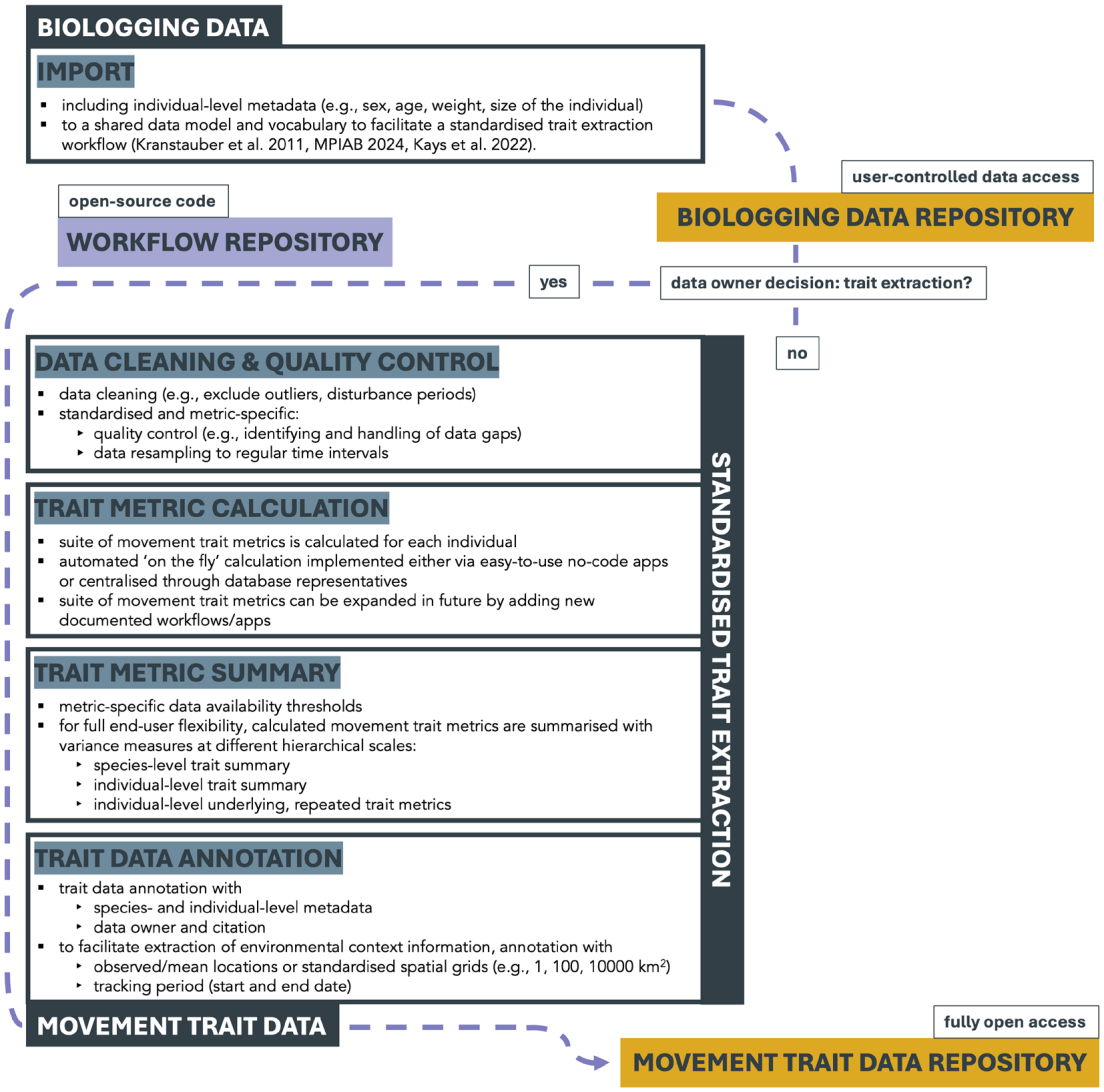
Envisioned standardised workflow from (1) animal biologging data import and organisation in a biologging data repository, to (2) standardised and metric‐specific data cleaning and quality control, trait metric calculation, summary and annotation, to (3) trait data archiving in a connected movement trait data repository (‘MoveTraits’ database). All workflow steps are automated and stored in a workflow repository as open‐source code. While data access in the biologging data repository is user‐controlled (with embargo and access restriction options to protect research agendas and ensure compliance with conservation requirements), all movement trait data is published fully open access, following the FAIR principles (Wilkinson et al. 2016) to improve the findability, accessibility, interoperability and reuse of data.

Ultimately, we envision an open, ‘living’ movement trait database established as an extension to one or several of the existing biologging databases. Many biologging databases exist (Davidson et al. 2025; Harcourt et al. 2019) with different regional, taxonomic (e.g., Urbano et al. 2021), or data/device type foci (e.g., Iverson et al. 2018). Combined, they store billions of animal locations and serve as ‘digital collections’ of animal movement behaviour (Kays et al. 2022; Wikelski et al. 2024). Automating the calculation of movement traits from these digital collections in a standardised workflow (Figure 4) is the logical next step. Over time, the movement trait database could thus grow alongside these databases in terms of regions, species, number of individuals covered and trait metrics extracted, advancing trait databases from relying on published reports to updating dynamically with incoming data. It could also extend trait calculations to additional dimensions of behaviour that can be captured by biologging, for example through measurements of acceleration, body temperature and heart rate (Hussey et al. 2015; Kays et al. 2015). This automated trait curation process can be supported by parallel developments of data and metadata standards, and ‘stewardship’ for biologging data management (Davidson et al. 2025). Leveraging existing infrastructure and data to create and share movement traits will accelerate research in fields ranging from conservation biology to global change ecology, connecting researchers across fields.

Currently, the lack of publicly available movement data is a key obstacle to achieve the promising integration of movement and trait data: While trait databases include data across almost 20,000 species, only 1.8% of these species also have publicly available movement data in Movebank (Beltran et al. 2025). Of all the discoverable studies stored in the main community biologging databases, only 12% are currently available for public download; access to a large majority of data remains restricted, for example to support storage of real‐time and sensitive species locations (Davidson et al. 2025). However, databases support restricted sharing, thus facilitating participation by owners of restricted‐access data in biologging databases, who could choose to allow trait calculation from their tracked individuals without making their underlying biologging data public. For trait data calculated from restricted‐access biologging data, annotation with spatio‐temporally explicit environmental conditions would be enabled through the reporting of the study period and ‘spatially diffused’ coordinates on a standardised spatial grid, for example, at a 1, 100, or 10,000 km^2^ grid cell resolution (for details, see Supplementary Methods S2). This way, individuals' exact locations are protected, while critical information about the general spatial context of the individuals' movements can nonetheless be retrieved. Facilitating environmental data annotation is critical for movement traits, in particular because animals adjust movement first and foremost to the environment (Nathan et al. 2008).

Movebank is currently the largest animal movement database and provides a suite of tools for data cleaning, annotation, visualisation and automated analysis workflows (Kays et al. 2022; Kölzsch et al. 2022). Its MoveApps platform supports no‐code analysis using open‐source modules, creating a possibility for MoveTraits to be based on repeatable trait calculation procedures that are open for contribution by other researchers. We therefore suggest Movebank as the initial basis for MoveTraits v 0.1 and propose integrating the calculation and storage of movement trait data as an extension to the Movebank ‘ecosystem’. However, as interoperability between biologging databases grows (Davidson et al. 2025; Sequeira et al. 2021), MoveTraits should ultimately be expanded to extract traits across databases and be hosted independently.

### 4.2 Implementation Challenges

The integration of movement traits into trait‐based ecology faces several challenges. Foremost among these are size‐based constraints of biologging technology, which have led to a significant data bias towards larger species (Beltran et al. 2025; Kays et al. 2015). This gap currently hampers cross‐taxa generalisations but is expected to narrow as the miniaturisation of tags expands the range of trackable species. In addition, high‐resolution 3D path reconstruction for small species is increasingly facilitated through the development of novel non‐attached remote sensing devices, such as image‐based data collection capturing fine‐scale movements with sonar and video camera arrays (e.g., videography of flying insects, Vo‐Doan et al. 2024), or high‐frequency imaging sonar and automated object detection for fish (Handegard et al. 2012; Lopez‐Marcano et al. 2021). Integrating such data sources into the automated movement trait calculation would be an important future implementation step to incorporate small animals' movements at much smaller spatio‐temporal scales.

Another challenge is the scale‐sensitivity of movement metrics. Most methods for deriving metrics from animal movement data are sensitive to sampling rate and sampling duration (Calabrese et al. 2016). This scale‐sensitivity stems from interacting biases related to sampling frequency (De Solla et al. 1999), movement path tortuosity (Rowcliffe et al. 2012), tracking device measurement error (Ranacher et al. 2016) and multi‐scale autocorrelation inherent in movement data (Fleming et al. 2014). Consequently, current discrete‐time approaches risk reflecting sampling schedules rather than underlying movement processes. Here, novel continuous‐time approaches offer promising solutions for scale‐insensitive estimations (Calabrese et al. 2016), which should be adopted in future implementations.

Finally, biologging databases have been designed primarily to manage data for individual projects. Steps are needed to prepare these data for contribution to aggregate products like MoveTraits (Figure 4; Davidson et al. 2025; Sequeira et al. 2021). For example, data owners may need incentives to make their data fit for use by providing sufficient metadata (e.g., on sex, developmental stage/age) in a standardised way (Davidson et al. 2025; Schneider et al. 2019), applying study‐specific data cleaning and quality control, and uniquely identifying individuals across projects and databases (Wikelski et al. 2024). These steps are critical to avoid misinterpretation of data and wrong inference. In addition, to garner long‐lasting support and data contributions from the community, MoveTraits will rely on approaches to incorporate the expertise of contributors and credit them in ways that are recognised by citation trackers, employers and funding agencies.

### 4.3 Novel Research Avenues

A comprehensive database with movement traits provided at distinct hierarchical levels, interoperable with other trait databases, opens up new opportunities to address key eco‐evolutionary questions (Beltran et al. 2025; Box 4) and to improve global change predictions. Firstly, MoveTraits will enable comparative studies to understand the *patterns and causes of variation in movement traits*, that is, whether there are generalisable patterns between animal movement and other traits, and to which degree these relationships depend on the geographic setting and ecological context. For instance, it could facilitate studies investigating the existence of common cross‐taxa behavioural syndromes (Abrahms et al. 2017; Vander Wal et al. 2022) or exploring the role of learning and memory in animal movement behaviour (Fagan et al. 2013; Lewis et al. 2021). In particular, MoveTraits could serve as an important data basis for the recently proposed field of macrobehaviour which aims to integrate behavioural ecology and macroecology to investigate behavioural variation across space, time and taxa (Brehm and Orrock 2024; Keith et al. 2023).

BOX 4 **Key eco‐evolutionary questions to be addressed with the MoveTraits database.**

We foresee exciting research questions along four main themes that could be addressed with MoveTraits. We highlight a few examples below:

1. *Understanding patterns and causes of variation in animal movement*
  - Are some species more consistent in their movement trait expression while others show greater variability, akin to movement specialists and generalists?
  - To what degree are species movement patterns shaped by other species traits (such as longevity, social behaviour, trophic level, breeding strategy) and/or by geographic and ecological context (e.g., climatic variation, trophic complexity)?
  - Is between‐individual variation in movement trait expression linked to variation in fitness (i.e., survival, reproductive success) and consequently leading to shifts in trait composition over time?
  - How do movement traits develop during ontogeny? Do species with complex ontogeny exhibit greater within‐individual variation in movement traits?
  - How do environmental stressors, pollutants, infections, and anthropogenic disturbances affect movement and behavioural patterns?
2. *Ecological consequences of movement trait variation*
  - How does movement trait expression affect species interactions, such as trophic, competitive, or mutualistic interactions?
  - Can movement traits capture species' roles in ecological communities and contributions to ecosystem functioning?
  - How does variation in movement behaviour contribute to biogeographic patterns of species distributions, biodiversity and ecosystem functioning?
  - How does individual behaviour affect individual contributions to ecosystem functions? How can this be accounted for in assessments of ecosystem functioning?
  - How do infection‐related behavioural changes influence spatiotemporal disease outbreak dynamics? Do ‘superspreaders’ differ in their movement behaviour?
3. *Evolutionary origins and consequences of movement trait variation*
  - What are the evolutionary origins of movement traits?
  - Has the diversity of movement traits increased or decreased over time and why?
  - Are species that display greater within‐ or between‐individual behavioural variance more likely to acclimate/adapt to rapid environmental changes than species with less variance?
  - What are the fitness consequences of divergent behavioural strategies and how stable are such strategies through time or across distinct populations?
4. *Integrating animal behaviour into global change predictions*
  - Can incorporating animal movement behaviour into predictive models help to better understand individual, population, community and ecosystem responses to environmental change?
  - Which environmental and anthropogenic stressors have the most profound impacts on movement traits and their associated ecological processes and ecosystem functions?
  - Can the degree of movement specialisation predict whether a species can persist or alter its range under scenarios of global change?

Secondly, MoveTraits will improve our ability to study the *ecological consequences of movement trait variation* by providing rich data to (a) map ecological interactions in space and time and (b) investigate how variation in movement affects ecosystem functioning. This will afford novel insights into crucial processes such as seed dispersal, predator–prey dynamics, pollination, nutrient transfer or disease transmission across landscapes, linking individual‐level behaviours to ecosystem‐level processes (e.g., Graf et al. 2024) and animal species to ecosystem services (e.g., Savoca et al. 2021). Work on animal‐mediated seed dispersal highlights this potential: biologging data provide fine‐scale insights into the spatio‐temporal patterns of animal movement behaviour (e.g., diel patterns of seed dispersal) typically masked in species‐level (proxy) traits and offer new insights into seed dispersal kernels and the coevolution of plants and their dispersers (Kays et al. 2011). In addition, it shows that between‐individual variation in animal movement impacts seed dispersal, with critical implications for plant species colonisation and habitat recovery (Graf et al. 2024; Russo et al. 2024). Another case in point is wildlife diseases, where MoveTraits could help harness biologging data to enhance outbreak detection, track spatiotemporal transmission dynamics, assess ecological impacts, and guide interventions by providing data to establish behavioural baselines, identify infection‐driven behavioural changes and reveal contact networks and shared resource use (Lopes et al. 2021; Talmon et al. 2025).

Thirdly, MoveTraits will improve our capability to assess the evolutionary origins and consequences of movement trait variation. Combining movement trait data with species ecology and evolutionary history provides us with unique possibilities to examine the evolutionary origins of animal movement, and how movement traits have evolved over space and time. For example, we can examine which biotic or abiotic factors have triggered evolutionary events and the diversification of movement traits (Abraham et al. 2022). In addition, MoveTraits will open up new avenues to predict the adaptive evolutionary potential of movement traits and animal behaviour by quantifying between‐individual variation in mean behaviour as well as behavioural plasticity (Hertel et al. 2020, 2021). For instance, while Devarajan et al. (2025) found diel activity to be highly plastic at the species level (often contradicting classifications from the reference literature), their extensive global camera trapping dataset cannot resolve between‐ or within‐individual plasticity. Diel activity patterns are of high eco‐evolutionary importance, for example as a mechanism to adjust to increased human activity and through their impact on species interactions. As the first database to provide methodologically consistent, empirical estimates of multi‐scale variation in diel activity, MoveTraits will expand our possibilities to investigate the extent, drivers, and eco‐evolutionary implications of circadian plasticity across scales and taxa.

Finally, MoveTraits will facilitate the *integration of animal behaviour into global change prediction*. While changes in species abundance, distribution, physiology, morphology, and phenology in response to global change have been widely documented, the role of animal behaviour as a mechanism for acclimation and adaptation remains underexplored (Beever et al. 2017; Buchholz et al. 2019). This oversight is problematic as it neglects a key pathway through which animals can react to rapidly changing conditions. Therefore, incorporating animal behaviour into predictive models is essential for anticipating individual, population, and community responses under future environmental change scenarios (Gil et al. 2020; Mason et al. 2014; Mathewson et al. 2017). Especially, the ability to account for individual behavioural reaction norms will boost a more mechanistic understanding—critical to robust predictions of altered dynamics under novel conditions (Moran et al. 2016).

### 4.4 Relevance for Global Biodiversity Assessments and Biodiversity Policy

Movement traits derived from biologging data have significant potential to strengthen global biodiversity assessments. For example, MoveTraits could aid the monitoring of movement diversity as an aspect of biodiversity (Russo et al. 2025). Specific traits, such as migration distances, home range sizes, or daily movement rates, could form the basis for new Essential Biodiversity Variables (EBVs) or indicators of biodiversity health, making movement data more policy‐relevant (Jetz et al. 2019; Kissling et al. 2018; Pereira et al. 2013). Movement is already listed as an EBV under ‘species traits’ (https://geobon.org/ebvs/what‐are‐ebvs/), but without clear definition and ignoring within‐species variation. MoveTraits could address this gap by quantitatively characterising movement at multiple scales. Since all traits at the individual‐ and within‐individual level are time‐annotated, monitoring these variables could provide crucial information about species’ responses to environmental changes, habitat fragmentation or human disturbances (Ellis Soto et al. 2025). For instance, changes in migration patterns or home range sizes across populations or species could serve as early warning indicators of ecosystem stress or biodiversity loss; altered daily movement rates might indicate changes in habitat quality or resource availability. Such movement‐based EBVs would complement existing biodiversity indicators, offering a more comprehensive picture of ecosystem health and functioning.

Moreover, standardised movement traits could enhance the assessment of ecosystem services and Nature Contributions to People that depend on animal movement (Kremen et al. 2007), providing invaluable information for conservation planning and sustainable resource management. By making movement data more accessible and interpretable, MoveTraits can help bridge the gap between movement ecology research and biodiversity policy (Jeltsch et al. 2013). It would provide policymakers with robust and comparable quantitative metrics of animal behaviour and ecosystem functioning, facilitating evidence‐based decision‐making and success monitoring in conservation and species management.

## 5 Conclusion

The creation of a movement trait database provides a unique opportunity to unlock a valuable and rapidly growing pool of standardised behavioural information for trait‐based ecology. Our proposed database outlines a novel pathway to trait data curation that allows for semi‐automated, continuous growth instead of one‐off static summaries, enables greater data sharing by allowing data owners to contribute open‐access trait data while restricting access to the underlying biologging data and fosters an open‐source collaborative development of reproducible and standardised trait calculation workflows. The integration of movement behaviour into trait‐based ecology represents a significant step towards a more comprehensive understanding of community and ecosystem dynamics. By providing standardised, comparable measures of animal movement behaviour, movement traits will enrich eco‐evolutionary research and enhance our ability to predict ecosystem responses to global change. The new possibility to integrate individual plastic responses via behavioural reaction norms into trait‐based global change ecology constitutes a major conceptual advance and an effective link between the fields of trait‐based and behavioural ecology. Furthermore, the relevance of these movement traits extends beyond academic research into global biodiversity assessments and policy. As potential EBVs and indicators of ecosystem health, MoveTraits will make biologging data more accessible and meaningful to policymakers and conservation practitioners.

In an era of rapid environmental change, the ability to quantify and predict animal responses across scales—from individual behaviour to ecosystem functioning—is crucial. Our proposed movement trait database promises to be a valuable tool in this endeavour, representing a significant step towards a more mechanistic understanding of ecological systems, crucial for effective biodiversity conservation and environmental management.

## Author Contributions

Larissa T. Beumer, Anne G. Hertel, Raphaël Royauté, Marlee A. Tucker and Thomas Mueller conceived the idea. Anne G. Hertel led the development of the MoveTraits database with help from Anne K. Scharf and additional input from Larissa T. Beumer, Kamran Safi, Andrea Kölzsch, Sarah C. Davidson and Thomas Mueller. Larissa T. Beumer and Anne G. Hertel created the figures. Larissa T. Beumer wrote the manuscript with support from Anne G. Hertel and Thomas Mueller. All authors reviewed and edited the manuscript.

## Funding

This work was supported by the Hessian Ministry of Science and Research, Arts and Culture, LOEWE priority project Nature 4.0—Sensing Biodiversity. NASA's Ecological Forecasting Program, 80NSSC21K1182. HORIZON EUROPE Research and Innovation Programme, 101086640. German Federal Ministry for Research, Technology and Aeronautics, 01LC2320A. Deutsche Forschungsgemeinschaft, HE 8857/1‐1, NE 1863/2‐2.

## Supporting information


**Appendix S1:** ele70297‐sup‐0001‐AppendixS1.pdf.

**Appendix S2:** ele70297‐sup‐0002‐AppendixS2.pdf.

## Data Availability

The data and R code that support the findings of this study are openly available in the OpenScience Framework at https://osf.io/sp8z6/ (DOI 10.17605/OSF.IO/SP8Z6). Future updates to the database code can be found under the MoveTraits developmental version on GitHub https://github.com/annescharf/MoveTraitsDatabase/releases/tag/v0.1. Important: The MoveTraits database will be updated regularly. If you want to use MoveTraits, please download the most updated version from https://doi.org/10.6084/m9.figshare.28611890 (Hertel et al. 2025).
