# Supplementary material for: MoveTraits—A Database for Integrating Animal Behaviour Into Trait‐Based Ecology
Source: Ecol Lett. 2025 Dec 31;29(1):e70297. doi: 10.1111/ele.70297 (PMC12755192; doi:10.1111/ele.70297)
Supplement: Supplementary file 1 — Appendix S1: ele70297‐sup‐0001‐AppendixS1.pdf. [file ELE-29-0-s001.pdf]

Supplementary materials

MoveTraits – A database for integrating animal behaviour into trait-based ecology

Beumer, L.T.[1,2,*,†], Hertel, A.G.[3,*], Royauté, R.[4], Tucker, M.A.[5], Albrecht, J.[1], Beltran, R.S.[6], Cagnacci, F.[7], Davidson, S.C.[8,9], Dejid, N.[1], Kays, R.[10,11], Kölzsch, A.[8,12], Lohr, A.[10], Neuschulz, E.L.[1], Safi, K.[8], Scharf, A.K.[8], Schleuning, M.[1], Wikelski, M.[8,9] and Mueller, T.[1,13]

*Shared first authorship

[1] Senckenberg Biodiversity and Climate Research Centre (SBiK-F), Frankfurt am Main, Germany

[2] Department of Arctic Biology, The University Centre in Svalbard, Longyearbyen, Svalbard

[3] Behavioural Ecology, Department of Biology, Ludwig-Maximilians University of Munich, Planegg-Martinsried, Germany

[4] Université Paris-Saclay, INRAE, AgroParisTech, UMR EcoSys, Palaiseau, France

[5] Department of Environmental Science, Radboud Institute for Biological and Environmental Sciences, Radboud University, Nijmegen, Netherlands

[6] Department of Ecology and Evolutionary Biology, University of California Santa Cruz, Santa Cruz, USA

[7] Animal Ecology Unit, Research and Innovation Centre, Edmund Mach Foundation, Trento, Italy

[8] Department of Migration, Max Planck Institute of Animal Behavior, Radolfzell, Germany

[9] Department of Biology, University of Konstanz, Constance, Germany

[10] North Carolina Museum of Natural Sciences, Raleigh, USA

[11] Department of Forestry and Environmental Resources, North Carolina State University, Raleigh, USA

[12] Ecology Department, Radboud Institute for Biological and Environmental Sciences, Radboud University, Nijmegen, Netherlands

[13] Department of Biological Sciences, Goethe University, Frankfurt am Main, Germany

[†] Corresponding author: larissab@unis.no

Table S1: Initial suite of movement trait metrics calculated at the individual level

**Trait metric**	**Definition/ Calculation**	**Fix rate threshold**	**Data availability threshold**	**Values reported in MoveTraits database**
Displacement distances				
Hourly [m]	Straight-line displacement distance between subsequent 1h locations	hourly	167 locations (7 days)	Mean, median, CV, 5th and 95th percentile
Daily [m]	Straight-line displacement distance between subsequent 24 h relocations.	daily	30 locations (30 days)	Mean, median, CV, 5th and 95th percentile
Daily - maximum [m]	Observed maximum distance of all pairwise hourly distances in a 24 h period	hourly	Days with at least 12 locations; minimum 7 days	Mean, median, CV, 5th and 95th percentile<br><br>Summarized across all days within criteria
Weekly - maximum [m]	Observed maximum distance of all pairwise daily distances in a 1-week period	daily	Weeks with at least 5 relocations; minimum 14 weeks	Mean, median, CV, 5th and 95th percentile<br><br>Summarized across all weeks within criteria
Annual - maximum [m]	Observed maximum distance of all pairwise weekly distances in a 1-year period	weekly	Minimum 36 weeks, i.e. 9 months of data	Mean, median, CV, 5th and 95th percentile<br><br>Summarized across all years within criteria
Diel activity				
Annual Diurnality index	((Total movement distance during day / daylength) - (Total movement distance during night / nightlength)) / ((Total movement distance during day / daylength) + (Total movement distance during night / nightlength))	hourly	Calculated for days with minimum 19 locations	Mean, median, CV, 5th and 95th percentile<br><br>Summarized for entire track
Range size				
Daily	95% Minimum	hourly	12 locations/day	Mean, median, CV, 5th

	Convex Polygon			and 95th percentile<br><br>Summarized across all days within criteria
Weekly	95% Minimum Convex Polygon	hourly	84 hourly relocations (3.5 days)	Mean, median, CV, 5th and 95th percentile<br><br>Summarized across all weeks within criteria
Monthly	95% Minimum Convex Polygon	daily	14 days of data	Mean, median, CV, 5th and 95th percentile<br><br>Summarized across all months within criteria
Annual	95% Minimum Convex Polygon	weekly	36 weeks of data	Mean, median, CV, 5th and 95th percentile<br><br>Summarized across all years within criteria
Range use/Tortuosity				
Intensity of area use - daily	Cumulative hourly movement distance during 24 h, divided by the square root of the range size during the same 24 h	hourly	12 locations/day	Mean, median, CV, 5th and 95th percentile
Intensity of area use - monthly	Cumulative daily movement distance during a given month, divided by the square root of the range size during the same month	daily	14 days of data	Mean, median, CV, 5th and 95th percentile
Intensity of area use - annual	Cumulative weekly movement distance during a given year, divided by the square root of the range size during the same year	weekly	36 weeks of data	Mean, median, CV, 5th and 95th percentile

*Table S2: Suggested metadata to be included in future versions of a living animal movement trait database. This list could grow in the future, motivated by additions of new traits and trait-based analyses. Data automatically passed on from Movebank (or, in the future, other biologging repositories) are marked by *, data that can be derived directly from the provided biologging (meta-)data are marked by †, information types that may not be mandatory in biologging repositories but would be important for movement trait interpretation and therefore require additional contributions from the data owners are marked by ∞.*

**Metadata**	**Level**
Data owner*	Study
Data owner contact details*	Study
Study identifier (e.g., repository-specific study ID)*	Study
Licence type*	Study
Citation (e.g, DOI)*, ∞	Study
Individual ID*	Individual
Species*	Individual
Species common name†	Individual
Sex ∞	Individual
Age [years, juvenile, adult]∞	Individual
Body mass [g]∞	Individual
Tracking start and end date†	Individual
Most common sampling interval†	Individual
Mean location - entire track†	Individual
Mean location - daily, weekly, monthly, annual, either from the observed data or placed in a reference grid cell†	Intra-individual
Movement mode [walk, fly, swim, arboreal]†	Species
Sample size [n individuals in dataset]†	Species
Class (mammal, bird)†	Species

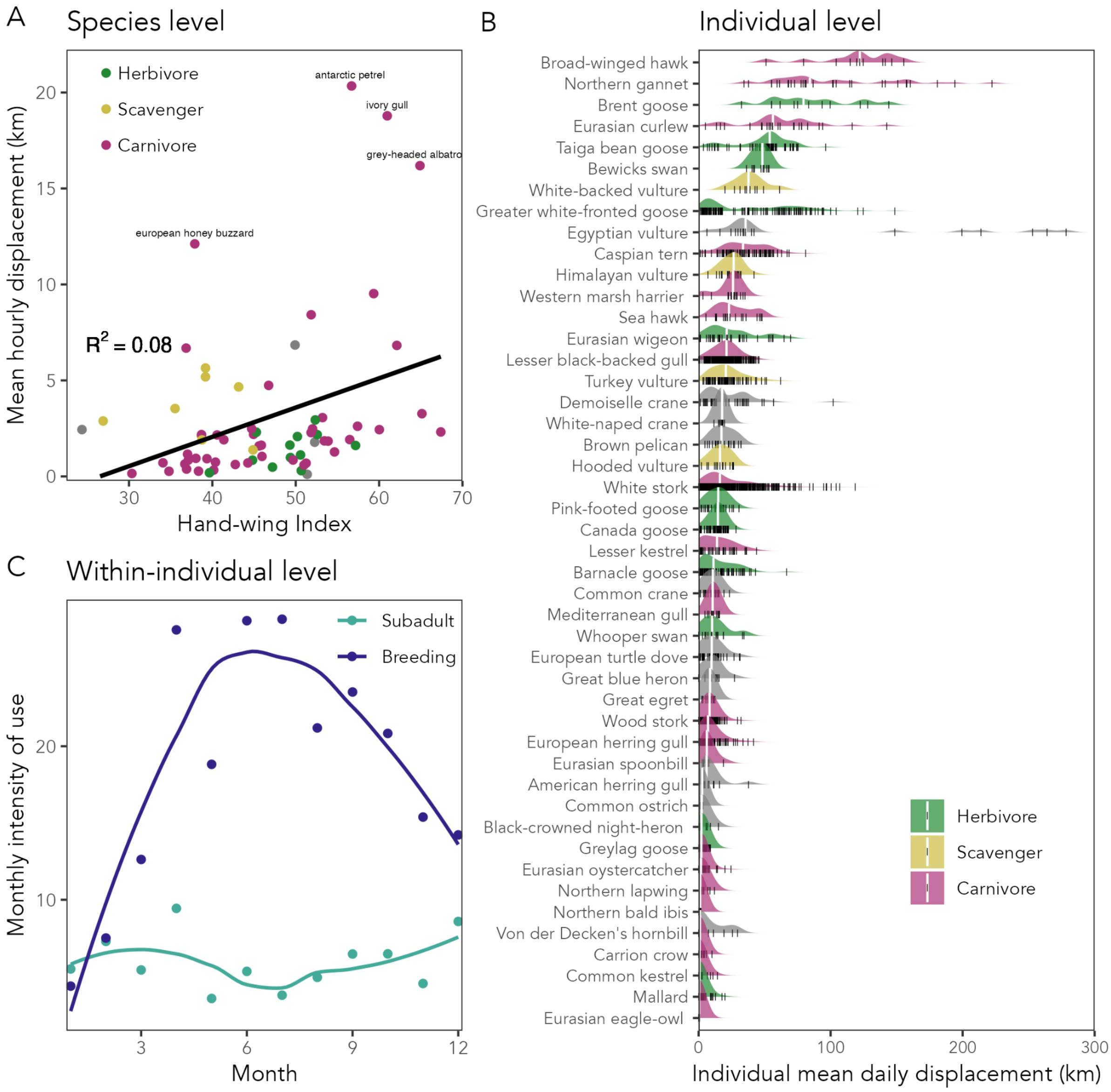

Figure S1: Illustrative case study examples for bird species in our MoveTraits database. (A) Hand-wing index is a common proxy for dispersal potential. Using 72 species for which hourly displacements were observed, the hand-wing index only correlated weekly with hourly displacements ($R^2 = 0.07$). (B) Between-individual variation in daily displacement across 46 bird species. Species are coloured according to their trophic position, as noted in the AVONET trait database. Gray shaded species were missing information for trophic position. (C) The monthly intensity of use for one Eurasian griffon vulture (*Gyps fulvus*) individual measures as a subadult in 2017 (green) and as a breeding adult in 2023 (purple). The monthly intensity of use is a measure of space use clusteredness. The individual shows more directed movements throughout the year as a subadult (green) while its breeding status is revealed through clustered movements throughout the summer (purple). Purple and green lines represent smoothers for each respective year over the twelve-month gradient. Data were sourced from the Movebank study ‘Raptors NABU Moessingen public’ (Study ID 186178781, Table S3).

Supplementary methods S1: Detailed description of database workflow

We have built a first proof-of-concept MoveTraits database for birds and mammals from open-access Movebank studies (licence types CC_0, CC_BY, CC_BY_NC, see below) and from an open-access dataset published by Tucker et al. (2023) and publicly available for download under: https://zenodo.org/records/7704108 (file 'Tucker_Road_Spatial.rds'). The workflow to reproduce this database and the MoveTraits database version 0.1.0 at the species, individual, and intra-individual level has been published under the Open Science Framework (https://doi.org/10.17605/OSF.IO/SP8Z6).

Licence types

We downloaded publicly available data from Movebank studies with the following open access licence types:

**Licence type**	**Licence terms**
CC_0	• No Copyright<br>• The person who associated a work with this deed has dedicated the work to the public domain by waiving all of his or her rights to the work worldwide under copyright law, including all related and neighbouring rights, to the extent allowed by law.<br>• You can copy, modify, distribute and perform the work, even for commercial purposes, all without asking permission. See Other Information below.<br>• Other Information:<br>○ In no way are the patent or trademark rights of any person affected by CC0, nor are the rights that other persons may have in the work or in how the work is used, such as publicity or privacy rights.<br>○ Unless expressly stated otherwise, the person who associated a work with this deed makes no warranties about the work, and disclaims liability for all uses of the work, to the fullest extent permitted by applicable law.<br>○ When using or citing the work, you should not imply endorsement by the author or the affirmer.<br>• https://creativecommons.org/publicdomain/zero/1.0/
CC_BY	• You are free to:<br>○ Share — copy and redistribute the material in any medium or format for any purpose, even commercially.<br>○ Adapt — remix, transform, and build upon the material for any purpose, even commercially.<br>• The licensor cannot revoke these freedoms as long as you follow the licence terms.<br>• Under the following terms:<br>○ Attribution — You must give appropriate credit, provide a link to the licence, and indicate if changes were made. You may do so in any reasonable manner, but not in any way that suggests the licensor endorses you or your use.<br>○ No additional restrictions — You may not apply legal terms or technological measures that legally restrict others from doing anything the licence permits.<br>• https://creativecommons.org/licenses/by/4.0/

CC_BY_NC	• You are free to:<br>○ Share — copy and redistribute the material in any medium or format<br>○ Adapt — remix, transform, and build upon the material<br>• The licensor cannot revoke these freedoms as long as you follow the licence terms.<br>• Under the following terms:<br>○ Attribution — You must give appropriate credit, provide a link to the licence, and indicate if changes were made . You may do so in any reasonable manner, but not in any way that suggests the licensor endorses you or your use.<br>○ NonCommercial — You may not use the material for commercial purposes.<br>○ No additional restrictions — You may not apply legal terms or technological measures that legally restrict others from doing anything the licence permits.<br>• https://creativecommons.org/licenses/by-nc/4.0/

We contacted all data owners about the use of their data in the MoveTraits database with the option to exclude their data from MoveTraits. Traits from two studies were removed.

Workflow details

1. The workflow for all biologging data downloaded from Movebank entailed data download and cleaning. We (a) removed records without location coordinates or necessary metadata such as taxon identity, (b) removed trajectories with too few observations (< 167 hourly locations, < 30 daily locations), (c) filtered for duplicates, e.g., in cases where tracks of some individuals are stored in multiple studies, (d) visually screened each movement trajectory for issues such as movements in fenced enclosures (and removed the respective individuals), and (e) removed the most obvious outliers with cut-off thresholds for implausible speeds (> 25 km/h for mammals and > 180 km/h for birds). Data downloaded from Tucker et al. (2023) was already cleaned. Given the different structure of the two data sources (individual 'move2' objects (Kranstauber *et al.* 2024) for Movebank and one dataframe for Tucker *et al.* (2023), traits were processed separately and trait databases derived from the two sources were merged in the end.
2. We resampled each individual track to a 1 hour ('hourly') relocation interval with a 15 minute tolerance, to a 24 hour ('daily') relocation interval with a 1 hour tolerance, and to a weekly (168 hours) relocation interval with a 1 day tolerance using the 'amt' R package (Signer *et al.* 2019). Resampling was not, at this stage, stratified to a specific minute of the hour (for hourly resampling) or to a specific hour of the day (for daily resampling). The coordinate reference system was WGS84 (EPSG 4326) unless specified otherwise.
3. We extracted displacement distances (hourly and daily) in meters from the resampled track using the step_lengths() function in the amt package. For maximum displacement distances (daily, weekly, annual) we calculated pairwise Euclidean distances within the focal time frame

using the st_distance() function from the 'sf' R package (Pebesma & Bivand 2023). For range size calculation (daily, weekly, monthly, annual) we transformed coordinates to the Mollweide equal area projection. We used the mcp() function in the R package 'adehabitatHR' (Calenge 2006) to calculate the 95% Minimum Convex Polygon of all points in the focal time period. We extracted the range size in m^2. To quantify intensity of use, we used the resampled data (e.g., hourly, daily, weekly) to calculate the cumulative distance during the focal time period (e.g. during a given month) and divided these by the area used during the same period (e.g., MCP during the same week/month). Diurnality was currently exclusively calculated for individuals with hourly data and only for days with at least 20 hourly locations. In the future, diurnality calculations from coarser intervals of two or three hours might be implemented.

4. We summarized traits at the individual level as mean, median, coefficient of variation (CV), 5th and 95th percentile of the underlying individual movement traits. We summarized these individual level traits at the species level as the mean of each summary (e.g., the mean of all individual 95th percentiles). We also provide a version of the database including the underlying individual movement traits (e.g., range size for every month the individual was tracked) alongside the individual's trait summary (e.g., mean monthly range size).
5. We finally merged the databases derived from Movebank data and from the Tucker et al. (2023) study and excluded duplicate individuals from the Tucker et al. (2023) database because it only contained traits based on three months of data (1 February - 15 May 2019).
6. We excluded any records from reptiles and fish as we were uncertain whether our traits would be informative for these taxonomic classes.

Supplementary methods S2: Spatial annotation outlook

Animals adjust movement first and foremost to the environment (Nathan et al., 2008). To enable ecological insights, measured trait values therefore need to be annotated with environmental conditions. To facilitate flexible trait annotation through the end user (based on specific study interests/needs), we annotate all movement traits with the spatial location and temporal context.

In the *current* version of the MoveTraits database, in which we only used open-access animal tracking data, we annotated *species* trait summaries with the species' averaged spatial coordinate, *individual* trait summaries with the individual's averaged spatial coordinate, and *within-individual* repeated trait measures with the observed coordinate for the given trait.

For *future* updates of the database, to allow for the spatial annotation of traits extracted from access-restricted data, we are envisioning an annotation using standardized spatial grids at cell sizes of 10000km^2 (distance between cell centroids ~100km), 100km^2 (distance ~10km), and 1km^2 (distance ~1km) (Fig. S2). We plan to annotate *species* trait summaries with all 10000km^2 grid cells occupied by the species, *individual* trait summaries with all grid cells at 100km^2 (mandatory) or 1km^2 (optional, user-defined), and *within-individual* trait measures with grid cells at 100km^2, 1km^2, or the observed coordinate (user defined). With this spatial diffusion approach, we hope to strike a compromise between facilitating flexible data annotation and protecting sensitive location information, to incorporate traits from as many species and individuals as possible.

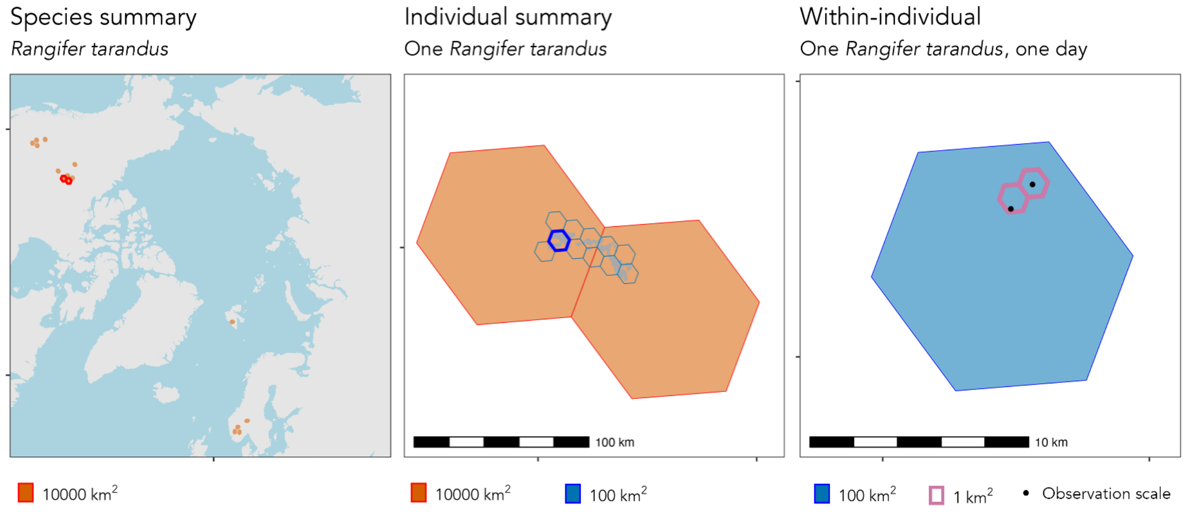

Figure S2: Spatial annotation using a standardized grid with cell sizes of 10000km^2 (distance between cell centroids ~100km), 100km^2 (distance ~10km), and 1km^2 (distance ~1km) across. We will annotate *species* traits with 10000km^2 grid cells. For restricted-access datasets, we will allow user-defined spatial annotation at 100km^2, 1km^2, or the observed coordinates at the *individual* and *within-individual* level.

References

Calenge, C. (2006). The package “adehabitat” for the R software: A tool for the analysis of space and habitat use by animals. *Ecol Modell*, 197, 516–519.

Kranstauber, B., Safi, K. & Scharf, A.K. (2024). move2: R package for processing movement data. *Methods Ecol Evol*, 15, 1561–1567.

Nathan, R., Getz, W.M., Revilla, E., Holyoak, M., Kadmon, R., Saltz, D. & Smouse, P.E. (2008). A movement ecology paradigm for unifying organismal movement research. *PNAS*, 105, 19052-19059.

Pebesma, E. & Bivand, R. (2023). Spatial Data Science: With Applications in R. *Spatial Data Science: With Applications in R*, 1–300.

Signer, J., Fieberg, J. & Avgar, T. (2019). Animal movement tools (amt): R package for managing tracking data and conducting habitat selection analyses. *Ecol Evol*, 9, 880–890.

Tucker, M.A., Schipper, A.M., Adams, T.S.F., Attias, N., Avgar, T., Babic, N.L., *et al.* (2023). Behavioral responses of terrestrial mammals to COVID-19 lockdowns. *Zenodo*. https://doi.org/10.5281/zenodo.7704108

Table S3. Overview of studies and their data owners included in the MoveTraits database version 0.1. Licence types are explained in the Supplementary methods S1. All raw data were standardised to hourly, daily, and weekly relocation intervals. Fixes at finer intervals were discarded. Traits were only calculated for datasets that fulfilled the minimum data requirements (Table S1). Studies listed informed at least one trait metric with at least one individual.

Source	Study ID	Species	Principal Investigator	Contact person	Citation	Recommended reference	License type
Movebank	82684	Bycanistes bucinator	Wolfgang Fiedler	Wolfgang Fiedler	Lenz J, Fiedler W, Caprano T, Friedrichs W, Gaese BH, Wikelski M, Böhning-Gaese K. 2011. Seed-dispersal distributions by trumpeter hornbills in fragmented landscapes. Proceedings of the Royal Society B Biological Sciences. https://doi.org/10.1098/rspb.2010.2383	https://doi.org/10.1098/rspb.2010.2383	CC_BY
Movebank	446579	Anas platyrhynchos	Martin Wikelski	Wolfgang Fiedler	Korner P, Sauter A, Fiedler W, Jenni L. 2016. Variable allocation of activity to daylight and night in the mallard. Animal Behaviour. 115: 69–79. https://doi.org/10.1016/j.anbehav.2016.02.026	https://doi.org/10.1016/j.anbehav.2016.02.026	CC_BY
Movebank	481458	Cathartes aura,Coragyps atratus	David Barber	David Barber	Bildstein KL, Barber D, Bechard MJ, Graña Grilli M, Therrien J. 2021. Data from: Study "Vultures Acopian Center USA GPS" (2003-2021). Movebank Data Repository. https://www.doi.org/10.5441/001/1.f3qt46r2 <br>Mallon JM, Bildstein KL, Fagan WF. 2021. Inclement weather forces stopovers and prevents migratory progress for obligate soaring migrants. Mov Ecol. 9:39. https://doi.org/10.1186/s40462-021-00274-6 <br>Graña Grilli M, Lambertucci SA, Therrien J-F, Bildstein KL. 2017. Wing size but not wing shape is related to migratory behavior in a soaring bird. J Avian Biol. 48(5):669-678. https://doi.org/10.1111/jav.01220 <br>Dodge S, Bohrer G, Bildstein K, Davidson SC, Weinzierl R, Mechard MJ, Barber D, Kays R, Brandes D, Han J, et al. 2014. Environmental drivers of variability in the movement ecology of turkey vultures (Cathartes aura) in North and South America. Philos T Roy Soc B. 369(1643):20130195. https://doi.org/10.1098/rstb.2013.0195 <br>Portions of these data are published with DOIs 10.5441/001/1.37r2b884 (study "Vultures Acopian Center USA 2003-2016") and 10.5441/001/1.46ft1k05 (study "Turkey vultures in North and South America (data from Dodge et al. 2014)")	https://doi.org/10.5441/001/1.f3qt46r2	CC_BY

Source	Study ID	Species	Principal Investigator	Contact person	Citation	Recommended reference	License type
Movebank	1764627	Syncerus caffer	Paul Cross	Paul Cross	Cross PC, Bowers JA, Hay CT, Wolhuter J, Buss P, Hofmeyr M, du Toit JT, Getz WM. 2016. Data from: Nonparameteric kernel methods for constructing home ranges and utilization distributions. Movebank Data Repository. https://www.doi.org/10.5441/001/1.j900f88t <br>Calabrese JM, Fleming CH, Gurarie E. 2016. ctmm: an r package for analyzing animal relocation data as a continuous-time stochastic process. Methods Ecol Evol. 7(9):1124-1132. https://doi.org/10.1111/2041-210X.12559 <br>Cross PC, Heisey DM, Bowers JA, Hay CT, Wolhuter J, Buss P, Hofmeyr M, Michel AL, Bengis RG, Bird TLF, et al. 2009. Disease, predation and demography: assessing the impacts of bovine tuberculosis on African buffalo by monitoring at individual and population levels. J Appl Ecol. 46(2):467-475. https://doi.org/10.1111/j.1365-2664.2008.01589.x <br>Getz WM, Fortmann-Roe S, Cross PC, Lyons AJ, Ryan SJ, Wilmers CC. 2007. LoCoH: Nonparameteric kernel methods for constructing home ranges and utilization distributions. PLoS ONE. 2(2):e207. https://doi.org/10.1371/journal.pone.0000207	https://doi.org/10.5441/001/1.j900f88t	CC_0
Movebank	1818825	Loxodonta cyclotis	Stephen Blake	Stephen Blake		Movebank study 'Forest Elephant Telemetry Programme'. Accessed on [date].	CC_BY
Movebank	1902221	Pecari tajacu	Roland Kays	Roland Kays	No papers published	Movebank study 'Collared Peccary on BCI'. Accessed on [date].	CC_BY
Movebank	2919708	Gyps africanus,Torgos tracheliotus	Ran Nathan	Orr Spiegel	Spiegel O, Getz WM, Nathan R. 2014. Data from: Factors influencing foraging search efficiency: Why do scarce lappet-faced vultures outperform ubiquitous white-backed vultures? (V2). Movebank Data Repository. https://www.doi.org/10.5441/001/1.mf903197 <br>Spiegel O, Getz WM, Nathan R. 2013. Factors influencing foraging search efficiency: why do scarce lappet-faced vultures outperform ubiquitous white-backed vultures? Am Nat. 181(5):E102-115. https://www.doi.org/10.1086/670009	https://doi.org/10.5441/001/1.mf903197	CC_0
Movebank	2927282	Anas platyrhynchos,Anas penelope,Aythya ferina	Wolfgang Fiedler	Wolfgang Fiedler	Data by Max Planck Institute for Animal Behavior, Vogelwarte Radolfzell, Germany.	Movebank study 'Lake Constance Ducks'. Accessed on [date].	CC_BY

Source	Study ID	Species	Principal Investigator	Contact person	Citation	Recommended reference	License type
Movebank	2930072	Steatornis caripensis	Martin Wikelski	Martin Wikelski	Holland RA, Wikelski M, Kuemmeth F, Bosque C. 2012. Data from: The secret life of oilbirds: new insights into the movement ecology of a unique avian frugivore. Movebank Data Repository. https://www.doi.org/10.5441/001/1.35fs26kq <br>Holland RA, Wikelski M, Kuemmeth F, Bosque C. 2009. The secret life of oilbirds: new insights into the movement ecology of a unique avian frugivore. PLoS ONE. 4(12):e8264. https://doi.org/10.1371/journal.pone.0008264	https://doi.org/10.5441/001/1.35fs26kq	CC_0
Movebank	2943485	Aquila chrysaetos,Haliaeetus leucocephalus,Pandion haliaetus,Falco peregrinus,Asio flammeus	Roland Kays	Roland Kays	Nye P, Hewitt G, Swenson T, Kays R. 2018. Data from: New York State bald eagle report 2010. Movebank Data Repository. https://www.doi.org/10.5441/001/1.s65q50j0 <br>Li Z, Han J, Ding B, Kays R. 2012. Mining periodic behaviors of object movements for animal and biological sustainability studies. Dat Min Knowl Disc. 24(2):355-386. https://doi.org/10.1007/s10618-011-0227-9 <br>Nye P. 2010. New York State bald eagle report 2010. Albany (NY): New York State Department of Environmental Conservation. 43 p. <br>Martell MS, Henny CJ, Nye PE, Solensky MJ. 2001. Fall migration routes, timing, and wintering sites of North American ospreys as determined by satellite telemetry. Condor. 103(4):715-724. https://doi.org/10.1650/0010-5422(2001)103[0715:FMRTAW]2.0.CO;2 <br>Rodriguez F, Martell M, Nye P, Bildstein KL. 2001. Osprey migration through Cuba. In: Bildstein K, Klem D Jr, editors. Hawkwatching in the Americas. North Wales (PA): Hawk Migration Association of North America. p. 107-117. <br>Bautz L, Nye P. 1987. Spring movement of an adult bald eagle from southeastern New York to central Ontario. The Eyas. 10(1):32-33.	https://doi.org/10.5441/001/1.s65q50j0	CC_0
Movebank	2980294	Anas strepera	Andrea Gehrold	Wolfgang Fiedler	Gehrold A, Bauer H-G, Fiedler W, Wikelski M. 2014. Great flexibility in autumn movement patterns of European Gadwalls (Anas strepera). Journal of Avian Biology. 45: 131–139. https://doi.org/10.1111/j.1600-048X.2013.00248.x<br><br>A subset of these data have been published as<br>Gehrold A, Wikelski M. 2013. Data from: Great flexibility in autumn movement patterns of European Gadwalls (Anas strepera). Movebank Data Repository. https://doi.org/10.5441/001/1.26dg08hv	https://doi.org/10.5441/001/1.26dg08hv	CC_BY
Movebank	2988309	Phalacrocorax carbo	Wolfgang Fiedler	Wolfgang Fiedler		Movebank study 'Great Cormorant Lake Constance MPIAB'. Accessed on [date].	CC_BY

Source	Study ID	Species	Principal Investigator	Contact person	Citation	Recommended reference	License type
Movebank	2988333	Larus fuscus	Martin Wikelski	Martin Wikelski	Wikelski M, Arriero E, Gagliardo A, Holland R, Huttunen MJ, Juvaste R, Mueller I, Tertitski G, Thorup K, Wild M, Alanko M, Bairlein F, Cherenkov A, Cameron A, Flatz R, Hannila J, Hüppop O, Kangasniemi M, Kranstauber B, Penttinen M, Safi K, Semashko V, Schmid H, Wistbacka R. 2015. Data from: True navigation in migrating gulls requires intact olfactory nerves. Movebank Data Repository. https://doi.org/10.5441/001/1.q986rc29 <br>Wikelski M, Arriero E, Gagliardo A, Holand R, Huttunen MJ, Juvaste R, Mueller I, Tertitski G, Thorup K, Wild M, et al. 2015. True navigation in migrating gulls requires intact olfactory nerves. Sci Rep. 5:17061. https://doi.org/10.1038/srep17061	https://doi.org/10.5441/001/1.q986rc29	CC_0
Movebank	3109235	Anas platyrhynchos	Jonas Waldenström	Jonas Waldenström	Bengtsson, D., Safi, K., Avril, A., Fiedler, W., Wikelski, M., Gunnarsson, G., Elmberg, J., Tolf, C., Olsen, B. & Waldenström, J. 2016. Does influenza A virus infection affect movement behaviour during stopover in its wild reservoir host? Royal Society Open Science 3: 150633. [http://dx.doi.org/10.1098/rsos.150633]. <br>Bengtsson, D., Avril, A., Gunnarsson, G., Elmberg, J., Söderquist, P., Norevik, G., Tolf, C., Safi, K., Fiedler, W., Wikelski, M., Olsen, B. & Waldenström, J. 2014. Movements, home-range size and habitat selection of Mallards during fall migration. PLOS ONE 9: 00764 [doi: 10.1371/journal.pone.0100764] 	https://doi.org/10.1098/rsos.150633	CC_BY_NC
Movebank	3780829	Bubo bubo	Martin Wikelski	Wolfgang Fiedler	Reinhard Vohwinkel et al. in prep.	Movebank study 'Eagle owl Reinhard Vohwinkel MPIAB'. Accessed on [date].	CC_BY
Movebank	4124813	Grus nigricollis	Sherub Sherub	Sherub Sherub	Sherub S, Wikelski M. 2024. Data from: Study "Black-necked crane Bhutan (UWICE-MPIAB)". Movebank Data Repository. https://doi.org/10.5441/001/1.601 <br>These data are described in<br>Yanco SW, Oliver RY, Iannarilli F, Carlson BS, Heine G, Mueller U, Richter N, Vorneweg B, Andryushchenko Y, Batbayar N, et al. 2024. Migratory birds modulate niche tradeoffs in rhythm with seasons and life history. Proc Natl Acad Sci USA. https://doi.org/10.1073/pnas.2316827121	https://doi.org/10.5441/001/1.601	CC_BY
Movebank	6770990	Fregata magnificens	Martin Wikelski	Martin Wikelski	Wikelski et al., birds and hurricanes, in prep.	Movebank study 'MPIAB PNIC hurricane frigate tracking'. Accessed on [date].	CC_BY
Movebank	6925808	Martes pennanti	Scott LaPoint	Scott LaPoint	Paper: LaPoint, S, Gallery P, Wikelski M, Kays R (2013) Animal behavior, cost-based corridor models, and real corridors. Landscape Ecology, v 28 i 8, p 1615–1630. doi:10.1007/s10980-013-9910-0 LaPoint S, Gallery P, Wikelski M, Kays R. 2013. Data from: Animal behavior, cost-based corridor models, and real corridors. Movebank Data Repository. https://doi.org/10.5441/001/1.2tp2j43g	https://doi.org/10.5441/001/1.2tp2j43g	CC_BY_NC
Movebank	7002955	Ciconia ciconia	Ran Nathan	Shay Rotics	Rotics et al. in prep	Movebank study 'HUJ MPIAB White Stork GSM E-Obs'. Accessed on [date].	CC_BY

Source	Study ID	Species	Principal Investigator	Contact person	Citation	Recommended reference	License type
Movebank	7023252	Papio anubis	Meg Crofoot	Meg Crofoot	Crofoot MC, Kays RW, Wikelski M. 2021. Data from: Study "Collective movement in wild baboons". Movebank Data Repository. https://www.doi.org/10.5441/001/1.3q2131q5 <br>Harel R, Loftus JC, Crofoot MC. 2021. Locomotor compromises maintain group cohesion in baboon troops on the move. P Roy Soc B. 288(1955):20210839. https://doi.org/10.1098/rspb.2021.0839 <br>Farine DR, Strandburg-Peshkin A, Couzin ID, Berger-Wolf TY, Crofoot MC. 2017. Individual variation in local interaction rules can explain emergent patterns of spatial organization in wild baboons. P Roy Soc B. 284(1853):20162243. https://doi.org/10.1098/rspb.2016.2243 <br>Strandburg-Peshkin A, Farine DR, Crofoot MC, Couzin ID. 2017. Habitat and social factors shape individual decisions and emergent group structure during baboon collective movement. eLife. 6:e19505. https://doi.org/10.7554/eLife.19505 <br>Farine DR, Strandburg-Peshkin A, Berger-Wolf T, Ziebart B, Brugere I, Li J, Crofoot MG. 2016. Both nearest neighbours and long-term affiliates predict individual locations during collective movement in wild baboons. Sci Rep. 6:27704. https://doi.org/10.1038/srep27704 <br>Strandburg-Peshkin A, Farine DR, Couzin ID, Crofoot MC. 2015. Shared decision-making drives collective movement in wild baboons. Science. 348–6241:1358–1361. https://doi.org/10.1126/science.aaa5099	https://doi.org/10.5441/001/1.3q2131q5	CC_0
Movebank	7431347	Ciconia ciconia	Movebank Administrator	Wolfgang Fiedler	Berthold P, Kaatz C, Kaatz M, Querner U, van den Bossche W, Chernetsov N, Fiedler W, Wikelski M. 2022. Data from: Study "MPIAB Argos white stork tracking (1991-2017)". Movebank Data Repository. https://www.doi.org/10.5441/001/1.k29d81dh	https://doi.org/10.5441/001/1.k29d81dh	CC_BY
Movebank	8019591	Bison bison	Stephen Blake	Stephen Blake		Movebank study 'Missouri Bison Tracking Project'. Accessed on [date].	CC_BY
Movebank	8849813	Ardea alba	Roland Kays	Roland Kays	Brzorad, J. N., M. C. Allen, S. Jennings, E. Condeso, S. Elbin, R. Kays, D. Lumpkin, S. Schweitzer, N. Tsipoura, and A. D. Maccarone. 2022. Seasonal Patterns in Daily Flight Distance and Space Use by Great Egrets (Ardea alba). Waterbirds 44:343–355.	https://doi.org/10.1675/063.044.0309	CC_0
Movebank	8927992	Anas platyrhynchos,Fulica atra	Martin Wikelski	Martin Wikelski	Wikelski et al., unpublished	Movebank study 'LifeTrack - Evros Delta'. Accessed on [date].	CC_BY
Movebank	9493881	Ciconia ciconia	Martin Wikelski	Wolfgang Fiedler	Data from study "LifeTrack White Stork Uzbekistan" in www.movebank.org by Max Planck institute of Animal Behavior (Radolfzell, Germany) and cooperation partners.	Movebank study 'LifeTrack White Stork Uzbekistan'. Accessed on [date].	CC_BY

Source	Study ID	Species	Principal Investigator	Contact person	Citation	Recommended reference	License type
Movebank	9651291	Neophron percnopterus	Evan Buechley	Evan Buechley	Buechley ER, Şekercioğlu CH. 2019. Data from: Satellite tracking a wide-ranging endangered vulture species to target conservation actions in the Middle East and East Africa. Movebank Data Repository. https://www.doi.org/10.5441/001/1.385gk270 <br>Phipps WL, López-López P, Buechley ER, Oppel S, Álvarez E, Arkumarev V, Bekmansurov R, Berger-Tal O, Bermejo A, Bounas A, Alanís IC, de la Puente J, Dobrev V, Duriez O, Efrat R, Fréchet G, García J, Galán M, García-Ripollés C, Gil A, Iglesias-Lebrija JJ, Jambas J, Karyakin IV, Kobierzycki E, Kret E, Loercher F, Monteiro A, Etxebarria JM, Nikolov SC, Pereira J, Peške L, Ponchon C, Realinho E, Saravia V, Şekercioğlu ÇH, Skartsi T, Tavares J, Teodósio J, Urios V, Vallverdú N. 2019. Spatial and temporal variability in migration of a soaring raptor across three continents. Front Ecol Evol. 7:323. https://doi.org/10.3389/fevo.2019.00323 <br>Buechley ER, McGrady MJ, Çoban E, Şekercioğlu ÇH. 2018. Satellite tracking a wide-ranging endangered vulture species to target conservation actions in the Middle East and East Africa. Biodivers Conserv. 27(9):2293-2310. https://doi.org/10.1007/s10531-018-1538-6 <br>Buechley ER, Oppel S, Beatty WS, Nikolov SC, Dobrev V, Arkumarev V, Saravia V, Bougain C, Bounas A, Kret E, Skartsi T, Aktay L, Ahababyan K, Frehner E, Şekercioğlu ÇH. 2018. Identifying critical migratory bottlenecks and high-use areas for an endangered migratory soaring bird across three continents. J Avian Biol. 49(7):e01629. https://doi.org/10.1111/jav.01629	https://doi.org/10.5441/001/1.385gk270	CC_BY
Movebank	10006517	Uria lomvia	Grant Gilchrist	Kyle Elliott		Movebank study 'Thick-billed Murres; Gilchrist; Digges Island, Canada'. Accessed on [date].	CC_0
Movebank	10157679	Ciconia ciconia	Azafzaf.H	Wolfgang Fiedler	Azafzaf, H., Feltrup-Azafzaf, C., Flack, A., Wikelski, M. & Fiedler, W. (year of access): GPS Tracking data from study "LifeTrack White Stork Tunisia" in www.movebank.org..	https://doi.org/10.1126/sciadv.1500931	CC_BY
Movebank	10204361	Pandion haliaetus	Barb Jensen	Barb Jensen		Movebank study 'Pandion haliaetus Osprey - SouthEast Michigan'. Accessed on [date].	CC_0

Source	Study ID	Species	Principal Investigator	Contact person	Citation	Recommended reference	License type
Movebank	10236270	Ciconia ciconia	Wolfgang Fiedler	Wolfgang Fiedler	Parts of this dataset are also available in the study "MPIO white stork lifetime tracking data (2013-2014)" and published as<br>Flack A, Fiedler W, Blas J, Pokrovski I, Mitropolsky B, Kaatz M, Aghababyan K, Khachatryan A, Fakriadis I, Makrigianni E, Jerzak L, Shamin M, Shamina C, Azafzaf H, Feltrup-Azafzaf C, Mokotjomela TM, Wikelski M. 2015. Data from: Costs of migratory decisions: a comparison across eight white stork populations. Movebank Data Repository. https://www.doi.org/10.5441/001/1.78152p3q <br>Flack A, Fiedler W, Blas J, Pokrovski I, Kaatz M, Mitropolsky M, Aghababyan K, Fakriadis Y, Makrigianni E, Jerzak L, et al. 2016. Costs of migratory decisions: a comparison across eight white stork populations. Science Advances. 2(1): e1500931. https://doi.org/10.1126/sciadv.1500931 <br>Kays R, Davidson SC, Berger M, Bohrer G, Fiedler W, Flack A, Hirt J, Hahn C, Gauggel D, Russell B, et al. 2021. The Movebank system for studying global animal movement and demography. Methods Ecol Evol. https://doi.org/10.1111/2041-210X.13767	https://doi.org/10.1111/2041-210X.13767	CC_BY
Movebank	10449318	Ciconia ciconia,Homo sapiens	Martin Wikelski	Wolfgang Fiedler	Data from study "LifeTrack White Stork Loburg" in www.movebank.org by Max Planck Institute of Animal Behavior (Radolfzell, Germany), Hebrew University of Jerusalem (Israel), University of Potsdam (Germany) and Storchenhof Loburg (Germany)	Movebank study 'LifeTrack White Stork Loburg'. Accessed on [date].	CC_BY
Movebank	10449535	Ciconia ciconia	Martin Wikelski	vasilis elias	Parts of this dataset are also available in the study "MPIO white stork lifetime tracking data (2013-2014)" and published as<br>Flack A, Fiedler W, Blas J, Pokrovski I, Mitropolsky B, Kaatz M, Aghababyan K, Khachatryan A, Fakriadis I, Makrigianni E, Jerzak L, Shamin M, Shamina C, Azafzaf H, Feltrup-Azafzaf C, Mokotjomela TM, Wikelski M. 2015. Data from: Costs of migratory decisions: a comparison across eight white stork populations. Movebank Data Repository. https://www.doi.org/10.5441/001/1.78152p3q <br>Flack A, Fiedler W, Blas J, Pokrovski I, Kaatz M, Mitropolsky M, Aghababyan K, Fakriadis Y, Makrigianni E, Jerzak L, et al. 2016. Costs of migratory decisions: a comparison across eight white stork populations. Science Advances. 2(1): e1500931. https://doi.org/10.1126/sciadv.1500931<br><br>Kays R, Davidson SC, Berger M, Bohrer G, Fiedler W, Flack A, Hirt J, Hahn C, Gauggel D, Russell B, et al. 2021. The Movebank system for studying global animal movement and demography. Methods Ecol Evol. https://doi.org/10.1111/2041-210X.13767	https://doi.org/10.1111/2041-210X.13767	CC_BY
Movebank	10449698	Ciconia ciconia	Ran Nathan	Shay Rotics	Rotics et al. unpublished data	Movebank study 'HUJ MPIAB White Stork GSM 2013'. Accessed on [date].	CC_BY

Source	Study ID	Species	Principal Investigator	Contact person	Citation	Recommended reference	License type
Movebank	10596067	Ciconia ciconia	Martin Wikelski	Andrea Flack	Parts of this dataset are also available in the study "MPIO white stork lifetime tracking data (2013-2014)" and published as<br>Flack A, Fiedler W, Blas J, Pokrovski I, Mitropolsky B, Kaatz M, Aghababyan K, Khachatryan A, Fakriadis I, Makrigianni E, Jerzak L, Shamin M, Shamina C, Azafzaf H, Feltrup-Azafzaf C, Mokotjomela TM, Wikelski M. 2015. Data from: Costs of migratory decisions: a comparison across eight white stork populations. Movebank Data Repository. https://www.doi.org/10.5441/001/1.78152p3q <br>Flack A, Fiedler W, Blas J, Pokrovski I, Kaatz M, Mitropolsky M, Aghababyan K, Fakriadis Y, Makrigianni E, Jerzak L, et al. 2016. Costs of migratory decisions: a comparison across eight white stork populations. Science Advances. 2(1): e1500931. https://doi.org/10.1126/sciadv.1500931<br><br>Kays R, Davidson SC, Berger M, Bohrer G, Fiedler W, Flack A, Hirt J, Hahn C, Gauggel D, Russell B, et al. 2021. The Movebank system for studying global animal movement and demography. Methods Ecol Evol. https://doi.org/10.1111/2041-210X.13767	https://doi.org/10.1111/2041-210X.13767	CC_BY
Movebank	10722328	Grus grus	Ramūnas Žydelis	Ramūnas Žydelis	Žydelis R, Desholm M, Månsson J, Nilsson L, Skov H. 2024. Data from: Study "GPS telemetry of Common Cranes, Sweden". Movebank Data Repository. https://doi.org/10.5441/001/1.597 <br>These data are described in<br>Yanco SW, Oliver RY, Iannarilli F, Carlson BS, Heine G, Mueller U, Richter N, Vorneweg B, Andryushchenko Y, Batbayar N, et al. 2024. Migratory birds modulate niche tradeoffs in rhythm with seasons and life history. Proc Natl Acad Sci USA. https://doi.org/10.1073/pnas.2316827121	https://doi.org/10.5441/001/1.597	CC_BY
Movebank	10763606	Ciconia ciconia	Martin Wikelski	Wolfgang Fiedler	Flack A, Fiedler W, Blas J, Pokrovsky I, Kaatz M, Mitropolsky M, Aghababyan K, Fakriadis I, Makrigianni E, Jerzak L, et al. 2016. Costs of migratory decisions: A comparison across eight white stork populations. Sci Adv. 2(1):e1500931. https://doi.org/10.1126/sciadv.1500931.	https://doi.org/10.1126/sciadv.1500931	CC_BY
Movebank	11223924	Anser fabalis	Tomas Aarvak	Tomas Aarvak		Movebank study 'Bean Goose Anser fabalis Finnmark.'. Accessed on [date].	CC_BY
Movebank	11948467	Didelphis virginiana,Procyon lotor	Roland Kays	Roland Kays		Movebank study 'NCSU Mammalogy Campus Carnivores'. Accessed on [date].	CC_0
Movebank	13978569	Phalcoboenus australis	David Barber	David Barber		Movebank study 'Striated Caracara Falkland Islands'. Accessed on [date].	CC_BY
Movebank	14288429	Equus hemionus	Petra Kaczensky	Petra Kaczensky	Kaczensky, P., Walzer, C. 2009. Gobi khulan GPS-ARGOS 2002-2008 tracking dataset.	Movebank study 'Khulan Mongolia GPS-ARGOS 2002-2008'. Accessed on [date].	CC_BY_NC
Movebank	14671003	Necrosyrtes monachus	David Barber	David Barber		Movebank study 'Hooded Vulture Africa'. Accessed on [date].	CC_BY

Source	Study ID	Species	Principal Investigator	Contact person	Citation	Recommended reference	License type
Movebank	15661938	Physeter macrocephalus	Daniel Palacios	Barb Lagerquist	Irvine LM, Follett TM, Winsor MH, Mate BR, Palacios DM. 2020. Data from: Study "Sperm whales Gulf of Mexico 2011-2013 - Argos data". Movebank Data Repository. doi: 10.5441/001/1.tj291471 <br>Irvine LM, Winsor MH, Follett TM, Mate BR, Palacios DM. 2020. An at-sea assessment of Argos location accuracy for three species of large whales, and the effect of deep-diving behavior on location error. Animal Biotelemetry. 8:20. doi: 10.1186/s40317-020-00207-x	https://doi.org/10.5441/001/1.44cb3946	CC_BY
Movebank	16880941	Cathartes aura	David Barber	David Barber	A more complete version of this dataset is stored in study "Vultures Acopian Center USA GPS".<br><br>Bildstein K, Barber D, Bechard MJ. 2014. Data from: Environmental drivers of variability in the movement ecology of turkey vultures (Cathartes aura) in North and South America. Movebank Data Repository. https://doi.org/10.5441/001/1.46ft1k05 <br>Dodge S, Bohrer G, Bildstein K, Davidson SC, Weinzierl R, Mechard MJ, Barber D, Kays R, Brandes D, Han J. 2014. Environmental drivers of variability in the movement ecology of turkey vultures (Cathartes aura) in North and South America. Philos T Roy Soc B. 369(1643):20130195. https://doi.org/10.1098/rstb.2013.0195	https://doi.org/10.5441/001/1.46ft1k05	CC_0
Movebank	17469219	Ardeidae ,Ardea herodias,Egretta thula,Ardea alba	John Brzorad	John Brzorad		Movebank study 'Egrets & Herons'. Accessed on [date].	CC_BY_NC
Movebank	19186107	Fregata magnificens	Patrick Jodice	Patrick Jodice	Jodice, P.G.R., K. Meyer, S. Zaluski, and L. Soanes	Movebank study 'Magnificent Frigatebird_BVI_GPS-PTT_2014-2016'. Accessed on [date].	CC_BY

Source	Study ID	Species	Principal Investigator	Contact person	Citation	Recommended reference	License type
Movebank	19411459	Panthera onca	Ronaldo Morato	Ronaldo Morato	Morato RG, Kantek DLZ, Miyazaki S, Deluque T, de Paula RC. 2021. Data from: Jaguar movement database: a GPS-based movement dataset of an apex predator in the Neotropics. Movebank Data Repository. https://www.doi.org/10.5441/001/1.3c4fv0m4 <br>These data are described in the following written publications:<br><br>Morato RG Thompson JJ, Paviolo A, De la Torre JA, Lima F, McBride Jr RT, De Paula RC, Cullen Jr L, Silveira L, Kantek DLZ, et al. 2018. Jaguar movement database: a GPS-based movement dataset of an apex predator in the Neotropics. Ecology. 99(7):1691. https://doi.org/10.1002/ecy.2379 <br>Morato RG, Connette GM, Stabach JA, De Paula RC, Ferraz KMPM, Kantek DLZ, Miyazaki SS, Pereira TDC, Silva LC, Paviolo A, et al. 2018. Resource selection in an apex predator and variation in response to local landscape characteristics. Biol Conserv. 228:233–40. https://doi.org/10.1016/j.biocon.2018.10.022 <br>Morato RG, Stabach JA, Fleming CH, Calabrese JM, De Paula RC, Ferraz KMPM, Kantek DLZ, Miyazaki SS, Pereira TDC, Araujo GR, et al. 2016. Space use and movement of a neotropical top predator: the endangered jaguar. PLoS ONE. 11(12):e0168176. https://doi.org/10.1371/journal.pone.0168176 <br>In addition, a version of these data are included as part of the following published datasets: <br>Morato RG, Thompson JJ, Paviolo A, de la Torre JA, Lima F, McBride Jr RT, Paula RC, Cullen Jr L, Silveira L, Kantek DLZ, et al. 2019. Data from: Jaguar Movement Database: a GPS-based movement dataset of an apex predator in the Neotropics. Dryad. https://doi.org/10.5061/dryad.2dh0223 <br>miltinhoastronauta. 2018. LEEClab/jaguar_movement: Jaguar Movement Database v1.0 released! (v1.0). Zenodo. https://doi.org/10.5281/zenodo.1345119 <br>https://github.com/LEEClab/jaguar_movement/tree/v1.0	https://doi.org/10.5441/001/1.3c4fv0m4	CC_0

Source	Study ID	Species	Principal Investigator	Contact person	Citation	Recommended reference	License type
Movebank	20202974	Gyps himalayensis	Martin Wikelski	Sherub Sherub	Portions of these data are published as<br>Sherub S and Wikelski M. 2021. Data from: Longer days enable higher diurnal activity for migratory birds [Himalayan griffons]. Movebank Data Repository. https://www.doi.org/10.5441/001/1.4n2501f5 <br>Sherub S, Wikelski M, Fiedler W, Davidson SC. 2016. Data from: Behavioural adaptations to flight into thin air. Movebank Data Repository. https://doi.org/10.5441/001/1.143v2p2k (study "High-altitude flights of Himalayan vultures (data from Sherub et al. 2016)") <br>Papers<br>Pokrovsky I, Kölzsch A, Sherub S, Fiedler W, Glazov P, Kulikova O, Wikelski M, Flack A. 2021. Longer days enable higher diurnal activity for migratory birds. J Anim Ecol. https://doi.org/10.1111/1365-2656.13484 <br>Sherub S, Bohrer G, Wikelski M, Weinzierl R. 2016. Behavioural adaptations to flight into thin air. Biology Letters. 12(10):20160432. https://doi.org/10.1098/rsbl.2016.0432	https://doi.org/10.5441/001/1.4n2501f5	CC_BY
Movebank	20873986	Haliaeetus leucocephalus	Roland Kays	Roland Kays	Roland Kays and Ted Simons	Movebank study 'LifeTrack Bald Eagle'. Accessed on [date].	CC_0

Source	Study ID	Species	Principal Investigator	Contact person	Citation	Recommended reference	License type
Movebank	21231406	*Ciconia ciconia*	Martin Wikelski	Wolfgang Fiedler	Fiedler W, Flack A, Schäfle W, Keeves B, Quetting M, Eid B, Schmid H, Wikelski M. 2024. Data from: Study "LifeTrack White Stork SW Germany" (2013-2023). Movebank Data Repository. https://doi.org/10.5441/001/1.ck04mn78_2 <br>These data are described in<br>Aikens EO, Nourani E, Fiedler W, Wikelski M, Flack A. 2024. Learning shapes the development of migratory behavior. Proc Natl Acad Sci USA. 121(12):e2306389121. https://doi.org/10.1073/pnas.2306389121<br><br>Kays R, Davidson SC, Berger M, Bohrer G, Fiedler W, Flack A, Hirt J, Hahn C, Gauggel D, Russell B, et al. 2022. The Movebank system for studying global animal movement and demography. Methods Ecol Evol. 13(2):419-431. https://doi.org/10.1111/2041-210X.13767 <br>Cheng Y, Fiedler W, Wikelski M, Flack A. 2019. "Closer-to-home" strategy benefits juvenile survival in a long-distance migratory bird. Ecology and Evolution. 9(16): 8945–8952. https://doi.org/10.1002/ece3.5395 <br>Flack A, Fiedler W, Blas J, Pokrovski I, Kaatz M, Mitropolsky M, Aghababyan K, Fakriadis Y, Makrigianni E, Jerzak L, et al. 2016. Costs of migratory decisions: a comparison across eight white stork populations. Science Advances. 2(1): e1500931. https://doi.org/10.1126/sciadv.1500931 <br>Weinzierl R, Bohrer G, Kranstauber B, Fiedler W, Wikelski M, Flack A. 2016. Wind estimation based on thermal soaring of birds. Ecology and Evolution. 6(24): 8706–8718. https://doi.org/10.1002/ece3.2585 <br>Parts of this dataset are also available in studies "Fall migration of white storks in 2014" (published as DOI 10.5441/001/1.bj96m274) and "MPIO white stork lifetime tracking data (2013-2014)" (published as DOI 10.5441/001/1.78152p3q).	https://doi.org/10.5441/001/1.ck04mn78_2	CC_BY

Source	Study ID	Species	Principal Investigator	Contact person	Citation	Recommended reference	License type
Movebank	24442409	Ciconia ciconia	Wolfgang Fiedler	Wolfgang Fiedler	Fiedler W, Leppelsack E, Leppelsack H, Stahl T, Wieding O, Wikelski M. 2024. Data from: Study "LifeTrack White Stork Bavaria" (2014-2023). Movebank Data Repository. https://doi.org/10.5441/001/1.v1cs4nn0_2 <br>Aikens EO, Nourani E, Fiedler W, Wikelski M, Flack A. 2024. Learning shapes the development of migratory behavior. Proc Natl Acad Sci USA. 121(12):e2306389121. https://doi.org/10.1073/pnas.2306389121<br><br>Brønnvik H, Nourani E, Fiedler W, Flack A. 2024. Experience reduces reliance on availability of conspecifics for route selection by a collectively migrating soaring bird. Curr Biol. 34(9):2030-2037.e3. https://doi.org/10.1016/j.cub.2024.03.052<br><br>Kays R, Davidson SC, Berger M, Bohrer G, Fiedler W, Flack A, Hirt J, Hahn C, Gauggel D, Russell B, et al. 2022. The Movebank system for studying global animal movement and demography. Methods Ecol Evol. 13(2):419-431. https://doi.org/10.1111/2041-210X.13767<br><br>Cheng Y, Fiedler W, Wikelski M, Flack A. 2019. "Closer-to-home" strategy benefits juvenile survival in a long-distance migratory bird. Ecology and Evolution. 9(16): 8945–8952. https://doi.org/10.1002/ece3.5395	https://doi.org/10.5441/001/1.v1cs4nn0_2	CC_BY
Movebank	28691134	Buteo platypterus	Laurie Goodrich	David Barber	contact PI	Movebank study 'Broad-winged Hawk habitat use, range, and movement ecology'. Accessed on [date].	CC_BY

Source	Study ID	Species	Principal Investigator	Contact person	Citation	Recommended reference	License type
Movebank	29799425	Branta leucopsis	Klaus-Michael Exo	Michael Exo	van der Jeugd H, Oosterbeek K, Ens BJ, Shamoun-Baranes J, Exo K. 2014. Data from: Forecasting spring from afar? Timing of migration and predictability of phenology along different migration routes of an avian herbivore [Barents Sea data]. Movebank Data Repository. https://www.doi.org/10.5441/001/1.ps244r11 <br>Shariati-Najafabadi M et al. 2016. Environmental parameters linked to the last migratory stage of barnacle geese en route to their breeding grounds. Animal Behavior. 118:81-95. https://doi.org/10.1016/j.anbehav.2016.05.018<br><br>Shariati-Najafabadi M et al. 2015. Satellite-versus temperature-derived green wave indices for predicting the timing of spring migration of avian herbivores. Ecological Indicators 58:322–331. https://doi.org/10.1016/j.ecolind.2015.06.005<br><br>Kölzsch A et al. 2015. Forecasting spring from afar? Timing of migration and predictability of phenology along different migration routes of an avian herbivore. J Anim Ecol. 84(1):272-283. https://doi.org/10.1111/1365-2656.12281<br><br>Shariatinajafabadi M et al. 2014. Migratory herbivorous waterfowl track satellite-derived green wave index. PLoS ONE 9(9):e108331. https://doi.org/10.1371/journal.pone.0108331<br><br>Ens BJ et al. 2008. Tracking of individual birds—report on WP 3230 (bird tracking sensor characterization) and WP 4130 (sensor adaptation and calibration for bird tracking system) of the FlySafe basic activities project. SOVON-onderzoeksrapport 2008/10, SOVON Vogelonderzoek Nederland, Beek-Ubbergen 78 p.	https://doi.org/10.5441/001/1.ps244r11	CC_0
Movebank	33622846	Uria lomvia	Grant Gilchrist	Kyle Elliott		Movebank study 'Thick-billed Murres; Gilchrist; Cape Graham Moore, Canada'. Accessed on [date].	CC_0
Movebank	33643212	Gyps fulvus,Gyps africanus,Torgos tracheliotus	Ran Nathan	Movement Ecology Lab at the Hebrew University of Jerusalem, Israel	Spiegel OM, Harel R, Centeno-Cuadros A, Hatzofe O, Getz WM, Nathan R. 2015. Data from: Moving beyond curve-fitting: using complementary data to assess alternative explanations for long movements of three vulture species. Movebank Data Repository. https://www.doi.org/10.5441/001/1.8c56f72s <br>Spiegel OM, Harel R, Centeno-Cuadros A, Hatzofe O, Getz WM, Nathan R. 2015. Moving beyond curve-fitting: using complementary data to assess alternative explanations for long movements of three vulture species. Am Nat. 185(2). https://www.doi.org/10.1086/679314	https://doi.org/10.5441/001/1.8c56f72s	CC_0

Source	Study ID	Species	Principal Investigator	Contact person	Citation	Recommended reference	License type
Movebank	37350671	Buceros bicornis,Rhyticeros undulatus	Aparajita Datta and Rohit Naniwadekar	Rohit	Naniwadekar R, Rathore A, Shukla U, Chaplod S, Datta A. 2019. Data from: How far do Asian forest hornbills disperse seeds? Movebank Data Repository. https://www.doi.org/10.5441/001/1.14sm8k1d <br>Naniwadekar R, Rathore A, Shukla U, Chaplod S, Datta A. 2019. How far do Asian forest hornbills disperse seeds? Acta Oecol. 101:103482. https://doi.org/10.1016/j.actao.2019.103482	https://doi.org/10.5441/001/1.14sm8k1d	CC_0
Movebank	40906102	Lemur catta	Teague O'Mara	Teague O'Mara	O'Mara. 2012. Development of feeding in ring-tailed lemurs. PhD Thesis. Arizona State University.	https://doi.org/http://hdl.handle.net/2286/R.A.93648	CC_0
Movebank	42451582	Numenius americanus	Jay Carlisle	Jay Carlisle		Movebank study 'Long-billed Curlew Migration from the Intermountain West'. Accessed on [date].	CC_BY_NC
Movebank	47450376	Grus antigone	Robert van Zalinge	Robert van Zalinge		Movebank study 'Sarus Crane, van Zalinge, Cambodia'. Accessed on [date].	CC_BY
Movebank	53460105	Papio anubis	Meg Crofoot	Meg Crofoot	Crofoot MC, Kays RW, Wikelski M. 2015. Data from: Shared decision-making drives collective movement in wild baboons. Movebank Data Repository. https://www.doi.org/10.5441/001/1.kn0816jn <br>Strandburg-Peshkin A, Farine DR, Couzin ID, Crofoot MC. 2015. Shared decision-making drives collective movement in wild baboons. Science. 348–6241: 1358–1361. https://doi.org/10.1126/science.aaa5099 <br>A larger dataset containing the data in this study is available as https://doi.org/10.5441/001/1.3q2131q5 and in the study "Collective movement in wild baboons".	https://doi.org/10.5441/001/1.kn0816jn	CC_BY
Movebank	67281010	Mycteria americana	Roland Kays	Roland Kays	Schweitzer S, Bryan AL Jr, Brzorad J, Kays R. 2023. Data from: Study "NC Wood Stork Tracking". Movebank Data Repository. https://doi.org/10.5441/001/1.303	Movebank study 'NC Wood Stork Tracking'. Accessed on [date].	CC_BY
Movebank	69724677	Branta bernicla,Branta leucopsis	Stefan Garthe	Stefan Garthe		Movebank study 'FTZ Geese Wadden Sea'. Accessed on [date].	CC_0

Source	Study ID	Species	Principal Investigator	Contact person	Citation	Recommended reference	License type
Movebank	74496970	Ciconia ciconia	Andrea Flack	Andrea Flack	Flack A, Fiedler W, Blas J, Pokrovski I, Mitropolsky B, Kaatz M, Aghababyan K, Khachatryan A, Fakriadis I, Makrigianni E, et al. 2015. Data from: Costs of migratory decisions: a comparison across eight white stork populations. Movebank Data Repository. https://www.doi.org/10.5441/001/1.78152p3q <br>Flack A, Fiedler W, Blas J, Pokrovski I, Kaatz M, Mitropolsky M, Aghababyan K, Fakriadis Y, Makrigianni E, Jerzak L, et al. 2016. Costs of migratory decisions: a comparison across eight white stork populations. Science Advances. 2(1): e1500931. https://doi.org/10.1126/sciadv.1500931<br><br>Kays R, Crofoot MC, Jetz W, Wikelski M. 2015. Terrestrial animal tracking as an eye on life and planet. Science. 348(6240):aaa2478. https://doi.org/10.1126/science.aaa2478	https://doi.org/10.5441/001/1.78152p3q	CC_BY
Movebank	76367850	Ciconia ciconia,Homo sapiens	Wolfgang Fiedler	Wolfgang Fiedler	Fiedler W, Hilsendegen C, Reis C, Lehmann J, Hilsendegen P, Schmid H, Wikelski M. 2024. Data from: Study "LifeTrack White Stork Rheinland-Pfalz" (2015-2023). Movebank Data Repository. https://doi.org/10.5441/001/1.4192t2j4_2 <br>Aikens EO, Nourani E, Fiedler W, Wikelski M, Flack A. 2024. Learning shapes the development of migratory behavior. Proc Natl Acad Sci USA. 121(12):e2306389121. https://doi.org/10.1073/pnas.2306389121<br><br>Brønnvik H, Nourani E, Fiedler W, Flack A. 2024. Experience reduces reliance on availability of conspecifics for route selection by a collectively migrating soaring bird. Curr Biol. 34(9):2030-2037.e3. https://doi.org/10.1016/j.cub.2024.03.052<br><br>Kays R, Davidson SC, Berger M, Bohrer G, Fiedler W, Flack A, Hirt J, Hahn C, Gauggel D, Russell B, et al. 2022. The Movebank system for studying global animal movement and demography. Methods Ecol Evol. 13(2):419-431. https://doi.org/10.1111/2041-210X.13767<br><br>Cheng Y, Fiedler W, Wikelski M, Flack A. 2019. "Closer-to-home" strategy benefits juvenile survival in a long-distance migratory bird. Ecology and Evolution. 9(16): 8945–8952. https://doi.org/10.1002/ece3.5395	https://doi.org/10.5441/001/1.4192t2j4_2	CC_BY
Movebank	79206236	Ciconia ciconia	Martin Wikelski	Wolfgang Fiedler	Grasso R, Gagliardo A, Zafarana M, Müller I, Schmid H, Fiedler W, Wikelski M. 2021. Data from: Study "LifeTrack White Stork Sicily". Movebank Data Repository. https://www.doi.org/10.5441/001/1.4v8q16qf <br>Kays R, Davidson SC, Berger M, Bohrer G, Fiedler W, Flack A, Hirt J, Hahn C, Gauggel D, Russell B, et al. 2021. The Movebank system for studying global animal movement and demography. Methods Ecol Evol. https://doi.org/10.1111/2041-210X.13767	https://doi.org/10.5441/001/1.4v8q16qf	CC_BY

Source	Study ID	Species	Principal Investigator	Contact person	Citation	Recommended reference	License type
Movebank	92261778	Cygnus cygnus	Dmitrijs Boiko	Wolfgang Fiedler	Dimitris Boiko, Wolfgang Fiedler & Martin Wikelski (2017): Project "Life Track Whooper Swan Latvia" at www.movebank.org	Movebank study 'LifeTrack Whooper Swan Latvia'. Accessed on [date].	CC_BY
Movebank	133992043	Anser albifrons	Andrea Kölzsch	Andrea Kölzsch	Kölzsch A, Kruckenberg H, Glazov P, Müskens GJDM, Wikelski M. 2016. Data from: Towards a new understanding of migration timing: slower spring than autumn migration in geese reflects different decision rules for stopover use and departure. Movebank Data Repository. https://www.doi.org/10.5441/001/1.31c2v92f <br>Kölzsch A, Müskens GJDM, Kruckenberg H, Glazov P, Weinzierl R, Nolet BA, Wikelski M. 2016. Towards a new understanding of migration timing: slower spring than autumn migration in geese reflects different decision rules for stopover use and departure. Oikos. 125(10):1496–1507. https://doi.org/10.1111/oik.03121	https://doi.org/10.5441/001/1.31c2v92f	CC_BY
Movebank	146932094	Grus grus	Mindaugas Dagys & Ramūnas Žydelis	Ramūnas Žydelis	Dagys M, Žydelis R. 2024. Data from: Study "Common Crane Lithuania GPS, 2015-2016". Movebank Data Repository. https://doi.org/10.5441/001/1.604 <br>These data are described in<br>Yanco SW, Oliver RY, Iannarilli F, Carlson BS, Heine G, Mueller U, Richter N, Vorneweg B, Andryushchenko Y, Batbayar N, et al. 2024. Migratory birds modulate niche tradeoffs in rhythm with seasons and life history. Proc Natl Acad Sci USA. https://doi.org/10.1073/pnas.2316827121	https://doi.org/10.5441/001/1.604	CC_BY
Movebank	150597370	Geronticus eremita	Didone Frigerio	Konrad Lorenz Research Station	Puehringer-Sturmayr V, Krejci J, Schuster R, et al. Space use and site fidelity in the endangered Northern Bald Ibis Geronticus eremita: Effects of age, season, and sex. Bird Conservation International. 2023;33:e10. doi:10.1017/S0959270922000053	https://doi.org/10.1017/S0959270922000053	CC_BY_NC
Movebank	154820583	Gyps fulvus	Goran Susic	Wolfgang Fiedler	Life Track Griffon Vulture Croatia Study	Movebank study 'LifeTrack Griffon Vulture Croatia'. Accessed on [date].	CC_BY
Movebank	172255794	Steatornis caripensis	David A. Rodríguez.	Bernd Vorneweg		Movebank study 'Lifetrack Oilbirds Costa Rica'. Accessed on [date].	CC_0

Source	Study ID	Species	Principal Investigator	Contact person	Citation	Recommended reference	License type
Movebank	172972156	Anthropoides virgo	Nyambayar Batbayar	Martin Wikelski	Batbayar N, Galtbalt B, Natsagdorj T, Sukhbaatar T, Wikelski M. 2024. Data from: Study "LifeTrack Mongolia Demoiselle cranes" Movebank Data Repository. https://doi.org/10.5441/001/1.599 <br>These data are described in<br>Yanco SW, Oliver RY, Iannarilli F, Carlson BS, Heine G, Mueller U, Richter N, Vorneweg B, Andryushchenko Y, Batbayar N, et al. 2024. Migratory birds modulate niche tradeoffs in rhythm with seasons and life history. Proc Natl Acad Sci USA. https://doi.org/10.1073/pnas.2316827121<br><br>Galtbalt B, Batbayar N, Sukhbaatar T, Vorneweg B, Heine G, Müller U, Wikelski M, Klaassen M. 2022. Differences in on-ground and aloft conditions explain seasonally different migration paths in demoiselle crane. Movement Ecology. https://doi.org/10.1186/s40462-022-00302-z <br>The processed data from Galtbalt et al. (2022) are published in Dryad at https://doi.org/10.5061/dryad.cnp5hqc1r	https://doi.org/10.5441/001/1.599	CC_BY_NC
Movebank	173641633	Ciconia ciconia	Wolfgang Fiedler	Wolfgang Fiedler	Fiedler W, Niederer W, Schönenberger A, Flack A, Wikelski M. 2024. Data from: Study "LifeTrack White Stork Vorarlberg" (2016-2023). Movebank Data Repository. https://doi.org/10.5441/001/1.71r7pp6q_2 <br>These data are described in<br>Aikens EO, Nourani E, Fiedler W, Wikelski M, Flack A. 2024. Learning shapes the development of migratory behavior. Proc Natl Acad Sci USA. 121(12):e2306389121. https://doi.org/10.1073/pnas.2306389121<br><br>Brønnvik H, Nourani E, Fiedler W, Flack A. 2024. Experience reduces reliance on availability of conspecifics for route selection by a collectively migrating soaring bird. Curr Biol. 34(9):2030-2037.e3. https://doi.org/10.1016/j.cub.2024.03.052<br><br>Cheng Y, Fiedler W, Wikelski M, Flack A. 2019. "Closer-to-home" strategy benefits juvenile survival in a long-distance migratory bird. Ecology and Evolution. 9(16): 8945–8952. https://doi.org/10.1002/ece3.5395	https://doi.org/10.5441/001/1.71r7pp6q_2	CC_BY
Movebank	175720577	Ciconia ciconia	Wolfgang Fiedler	Wolfgang Fiedler	Maxhuni Q, Gashi A, Hoxha L, Wolf G, Fiedler W. 2021. Data from: Study "LifeTrack White Stork Kosova". Movebank Data Repository. https://www.doi.org/10.5441/001/1.s367rd3k <br>Kays R, Davidson SC, Berger M, Bohrer G, Fiedler W, Flack A, Hirt J, Hahn C, Gauggel D, Russell B, et al. 2021. The Movebank system for studying global animal movement and demography. Methods Ecol Evol. https://doi.org/10.1111/2041-210X.13767	https://doi.org/10.5441/001/1.s367rd3k	CC_BY

Source	Study ID	Species	Principal Investigator	Contact person	Citation	Recommended reference	License type
Movebank	178979729	Canis lupus	Dave Latham, Stan Boutin	Maria Cecilia Latham	Latham ADM, Boutin S. 2019. Data from: Wolf ecology and caribou-primary prey-wolf spatial relationships in low productivity peatland complexes in northeastern Alberta. Movebank Data Repository. https://www.doi.org/10.5441/001/1.7vr1k987 <br>Latham AD. 2009. Wolf ecology and caribou-primary prey-wolf spatial relationships in low productivity peatland complexes in northeastern Alberta [dissertation]. [Alberta (CA)]: ProQuest Dissertations Publishing, University of Alberta. NR55419. 197 p. http://search.proquest.com/docview/305051214 <br>Latham AD, Latham MC, Boyce MS, Boutin S. 2011. Movement responses by wolves to industrial linear features and their effect on woodland caribou in northeastern Alberta. Ecol Appl. 21(8):2854–2865. https://doi.org/10.1890/11-0666.1	https://doi.org/10.5441/001/1.7vr1k987	CC_BY_NC
Movebank	180290122	Thalassoica antarctica	Descamps	Descamps	Descamps S, Tarroux A, Cherel Y, Delord K, Godø OR, Kato A, Krafft BA, Lorentsen S, Ropert-Coudert Y, Skaret G, Varpe Ø. 2016. Data from: At-sea distribution and prey selection of Antarctic petrels and commercial krill fisheries. Movebank Data Repository. https://www.doi.org/10.5441/001/1.q4gn4q56<br><br>These data are described in<br>Descamps S, Tarroux A, Cherel Y, Delord K, Godø OR, Kato A, Krafft BA, Lorentsen S-H, Ropert-Coudert Y, Skaret G, et al. 2016. At-sea distribution and prey selection of Antarctic petrels and commercial krill fisheries. PLoS ONE. 11(8):e0156968. https://doi.org/10.1371/journal.pone.0156968	https://doi.org/10.5441/001/1.q4gn4q56	CC_BY
Movebank	182459847	Cygnus cygnus	Martin Wikelski	Wolfgang Fiedler	Boiko D, Wikelski M, Fiedler W. 2019. Data from: Moulting sites of Latvian whooper swan Cygnus cygnus cygnets fitted with GPS-GSM transmitters. Movebank Data Repository. https://www.doi.org/10.5441/001/1.f89984gn <br>Boiko D, Wikelski M. 2019. Moulting sites of Latvian whooper swan Cygnus cygnus cygnets fitted with GPS-GSM transmitters. Wildfowl. 5: 229-241. https://wildfowl.wwt.org.uk/index.php/wildfowl/article/view/2714<br><br>Boiko D, Wikelski M. 2018. Abstract: Moulting sites of Latvian Whooper Swan Cygnus cygnus cygnets tagged with transmitters in summer 2016. p. 40: http://conference.emu.ee/userfiles/swan2018/Luigekonverents148X210web.pdf	https://doi.org/10.5441/001/1.f89984gn	CC_BY

Source	Study ID	Species	Principal Investigator	Contact person	Citation	Recommended reference	License type
Movebank	182746263	Gyps himalayensis	Martin Wikelski	Sherub Sherub	Sherub S, Wikelski M, Fiedler W, Davidson SC. 2016. Data from: Behavioural adaptations to flight into thin air. Movebank Data Repository. https://www.doi.org/10.5441/001/1.143v2p2k <br>Sherub S, Bohrer G, Wikelski M, Weinzierl R. 2016. Behavioural adaptations to flight into thin air. Biology Letters. 12(10):20160432. https://doi.org/10.1098/rsbl.2016.0432	https://doi.org/10.5441/001/1.143v2p2k	CC_0
Movebank	183209639	Larus fuscus	Stefan Garthe	Stefan Garthe	Garthe S, Schwemmer P, Paiva VH, Corman A-M, Fock HO, Voigt CC, Adler S (2016) Terrestrial and marine foraging strategies of an opportunistic seabird species breeding in the Wadden Sea. PLoS ONE 11(8): e0159630. doi:10.1371/journal.pone.0159630	https://doi.org/10.5441/001/1.nk286sc0	CC_BY_NC
Movebank	185780950	Geronticus eremita	Didone Frigerio	Konrad Lorenz Research Station	Puehringer-Sturmayr V, Krejci J, Schuster R, et al. Space use and site fidelity in the endangered Northern Bald Ibis Geronticus eremita: Effects of age, season, and sex. Bird Conservation International. 2023;33:e10. doi:10.1017/S0959270922000053	https://doi.org/10.1017/S0959270922000053	CC_BY_NC
Movebank	186178781	Pernis apivorus,Gyps fulvus	Daniel Schmidt-Rothmund	Wolfgang Fiedler		Movebank study 'Raptors NABU Moessingen public'. Accessed on [date].	CC_BY
Movebank	193545363	Canis latrans,Puma concolor	Julie Young, PhD	Peter Mahoney	Mahoney PJ, Ebinger M, Jaeger M, Shivik JA, Young JK. 2017. Data from: Uncovering behavioural states from animal activity and site fidelity patterns. Movebank Data Repository. https://www.doi.org/10.5441/001/1.7d8301h2 <br>These data are described in<br>Mahoney PJ, Young JK. 2017. Uncovering behavioural states from animal activity and site fidelity patterns. Methods Ecol Evol. 8(2):174-183. https://doi.org/10.1111/2041-210X.12658	https://doi.org/10.5441/001/1.7d8301h2	CC_0

Source	Study ID	Species	Principal Investigator	Contact person	Citation	Recommended reference	License type
Movebank	208413731	Connochaetes taurinus	Randall Boone	Jared Stabach	Stabach JA, Hughey L, Reid RS, Worden JS, Leimgruber P, Boone RB. 2020. Data from: Study "White-bearded wildebeest in Kenya". Movebank Data Repository. https://www.doi.org/10.5441/001/1.h0t27719 <br>Stabach JA, Hughey LF, Crego RD, Fleming CH, Hopcraft JGC, Leimgruber P, Morrison TA, Ogutu JO, Reid RS, Worden JS, Boone RB. 2022. Increasing anthropogenic disturbance restricts wildebeest movement across East African grazing systems. Front Ecol Evol. https://doi.org/10.3389/fevo.2022.846171 <br>Walton T, Boone R. 2017. Analyzing wildebeest behavior and effects of humans on activity in southwest Kenya. Poster [stored in this study; see Files] <br>Stabach JA. 2015. Movement, resource selection, and the physiological stress response of white-bearded wildebeest [dissertation]. [Fort Collins (CO, USA)]: Colorado State University. https://doi.org/10217/167207 <br>Boone RB, Reid RS, Lilieholm RJ, Worden JS, Ogutu JO. 2009. Wildebeest forage acquisition in fragmented landscapes under variable climates. National Science Foundation (NSF) DEB Grant 0919383.	https://doi.org/10.5441/001/1.h0t27719	CC_BY
Movebank	209824313	Canis lupus	Mark Hebblewhite	Mark Hebblewhite	Hebblewhite M, Merrill EH (2008) Modelling wildlife-human relationships for social species with mixed-effects resource selection models. Journal of Applied Ecology 45:834-844. doi:10.1111/j.1365-2664.2008.01466.x Hebblewhite M, Merrill EH (2007) Multiscale wolf predation risk for elk: does migration reduce risk? Oecologia 152:377-387. doi:10.1007/s00442-007-0661-y	https://doi.org/10.1111/j.1365-2664.2008.01466.x	CC_BY

Source	Study ID	Species	Principal Investigator	Contact person	Citation	Recommended reference	License type
Movebank	212096177	Ciconia ciconia	Wolfgang Fiedler	Wolfgang Fiedler	Fiedler W, Flack A, Schmid A, Reinhard U, Wikelski M. 2024. Data from: Study "LifeTrack White Stork Oberschwaben" (2014-2023). Movebank Data Repository. https://doi.org/10.5441/001/1.c42j3js7_2 <br>These data are described in<br>Aikens EO, Nourani E, Fiedler W, Wikelski M, Flack A. 2024. Learning shapes the development of migratory behavior. Proc Natl Acad Sci USA. 121(12):e2306389121. https://doi.org/10.1073/pnas.2306389121<br><br>Brønnvik H, Nourani E, Fiedler W, Flack A. 2024. Experience reduces reliance on availability of conspecifics for route selection by a collectively migrating soaring bird. Curr Biol. 34(9):2030-2037.e3. https://doi.org/10.1016/j.cub.2024.03.052<br><br>Kays R, Davidson SC, Berger M, Bohrer G, Fiedler W, Flack A, Hirt J, Hahn C, Gauggel D, Russell B, et al. 2021. The Movebank system for studying global animal movement and demography. Methods Ecol Evol. https://doi.org/10.1111/2041-210X.13767<br><br>Cheng Y, Fiedler W, Wikelski M, Flack A. 2019. "Closer-to-home" strategy benefits juvenile survival in a long-distance migratory bird. Ecology and Evolution. 9(16): 8945–8952. https://doi.org/10.1002/ece3.5395	https://doi.org/10.5441/001/1.c42j3js7_2	CC_BY

Source	Study ID	Species	Principal Investigator	Contact person	Citation	Recommended reference	License type
Movebank	216040785	Rangifer tarandus	Dale R. Seip	Sarah Davidson	Seip DR, Price E. 2019. Data from: Science update for the South Peace Northern Caribou (Rangifer tarandus caribou pop. 15) in British Columbia. Movebank Data Repository. https://www.doi.org/10.5441/001/1.p5bn656k <br>Seip D, Jones E. 2017. Population status of Central Mountain caribou herds in British Columbia and response to recovery management actions. Ministry of Environment and Climate Change Strategy. 18 p. https://www2.gov.bc.ca/assets/gov/environment/plants-animals-and-ecosystems/wildlife-wildlife-habitat/regional-wildlife/northeast-region/caribou/central_mountains_caribou_population_report_2017.pdf <br>Johnson CJ, Ehlers LPW, Seip DR. 2015. Witnessing extinction: cumulative impacts across landscapes and the future loss of an evolutionarily significant unit of woodland caribou in Canada. Biol Conserv. 186:176-186. https://doi.org/10.1016/j.biocon.2015.03.012 <br>BC Ministry of Environment. 2014. Science update for the South Peace Northern caribou (Rangifer tarandus caribou pop. 15) in British Columbia. Victoria, BC. 43 p. https://www2.gov.bc.ca/assets/gov/environment/plants-animals-and-ecosystems/wildlife-wildlife-habitat/caribou/science_update_final_from_web_jan_2014.pdf <br>Jones ES, Gillingham MP, Seip DR, Heard DC. 2007. Comparison of seasonal habitat selection between threatened woodland caribou ecotypes in central British Columbia. Rangifer. 27(4):111-128. https://doi.org/10.7557/2.27.4.325	https://doi.org/10.5441/001/1.p5bn656k	CC_BY
Movebank	217784323	Cathartes aura,Coragyps atratus	David Barber	David Barber	A more complete version of this dataset is stored in study "Vultures Acopian Center USA GPS".<br><br>Bildstein KL, Barber D, Bechard MJ, Graña Grilli M. 2016. Data from: Wing size but not wing shape is related to migratory behavior in a soaring bird. Movebank Data Repository. https://www.doi.org/10.5441/001/1.37r2b884 <br>Graña Grilli M, Lambertucci SA, Therrien J-F, Bildstein KL. 2017. Wing size but not wing shape is related to migratory behavior in a soaring bird. J Avian Biol. 48(5):669-678. https://doi.org/10.1111/jav.01220 <br>Dodge S, Bohrer G, Bildstein K, Davidson SC, Weinzierl R, Mechard MJ, Barber D, Kays R, Brandes D, Han J, et al. 2014. Environmental drivers of variability in the movement ecology of turkey vultures (Cathartes aura) in North and South America. Philos T Roy Soc B. 369(1643):20130195. https://doi.org/10.1098/rstb.2013.0195	https://doi.org/10.5441/001/1.37r2b884	CC_BY

Source	Study ID	Species	Principal Investigator	Contact person	Citation	Recommended reference	License type
Movebank	220078181	Thalassarche chrysostoma	David Thompson	Rachael Orben	Thompson DR, Torres LG, Sagar PM, Kroeger CE, Orben RA. 2017. Data from: Classification of animal movement behavior through residence in space and time. Movebank Data Repository. https://www.doi.org/10.5441/001/1.694p666h<br><br>Torres LG, Orben RA, Tolkova I, Thompson DR. 2017. Classification of animal movement behavior through residence in space and time. PLoS ONE. 12(1):e0168513. https://doi.org/10.1371/journal.pone.0168513	https://doi.org/10.5441/001/1.694p666h	CC_BY_NC
Movebank	227843945	Physeter macrocephalus	Daniel Palacios	Barb Lagerquist	Irvine L, Palacios DM, Urbán, Mate B. 2017. Sperm whale dive behavior characteristics derived from intermediate-duration archival tag data. Ecology and Evolution. 7:7822–7837. doi: 10.1002/ece3.3322	https://doi.org/10.5441/001/1.rj857167	CC_BY
Movebank	233141114	Grus vipio	Nyambayar Batbayar	Nyambayar Batbayar	Batbayar N, Galtbalt B, Natsagdorj T, Sukhbaatar T, Wikelski M. 2024. Data from: Study "White-naped crane Mongolia WSCC" Movebank Data Repository. https://doi.org/10.5441/001/1.600 <br>These data are described in<br>Yanco SW, Oliver RY, Iannarilli F, Carlson BS, Heine G, Mueller U, Richter N, Vorneweg B, Andryushchenko Y, Batbayar N, et al. 2024. Migratory birds modulate niche tradeoffs in rhythm with seasons and life history. Proc Natl Acad Sci USA. https://doi.org/10.1073/pnas.2316827121	https://doi.org/10.5441/001/1.600	CC_BY_NC
Movebank	236953686	Anas platyrhynchos	Wolfgang Fiedler	Wolfgang Fiedler	Duck data Max Planck Institute for Animal Behavior	Movebank study 'LifeTrack Ducks Lake Constance'. Accessed on [date].	CC_0
Movebank	249737372	Uria lomvia	Kyle Elliott	Allison Patterson		Movebank study 'Thick-billed murre Gilchrist and Elliott Digges 2016'. Accessed on [date].	CC_0
Movebank	268904527	Wallabia bicolor	Manuela Fischer	Manuela Fischer	Fischer M, Di Stefano J, Gras P, Kramer-Schadt S, Sutherland DR, Coulson G, Stillfried M (2020) Data from: Circadian rhythms enable efficient resource selection in a human-modified landscape. Movebank Data Repository. https://www.doi.org/10.5441/001/1.6ss053tn <br>Fischer M, Di Stefano J, Gras P, Kramer-Schadt S, Sutherland DR, Coulson G, Stillfried M (2019) Circadian rhythms enable efficient resource selection in a human-modified landscape. Ecology and Evolution 9(13): 7509–7527. https://doi.org/10.1002/ece3.5283	https://doi.org/10.5441/001/1.6ss053tn	CC_0
Movebank	270079388	Uria lomvia	Grant Gilchrist	Allison Patterson		Movebank study 'Thick-billed murre Gilchrist Cape Graham Moore 2016'. Accessed on [date].	CC_0
Movebank	277815715	Ichthyaetus audouinii	Faggio	G Faggio	Recorbet, B., Faggio, G. & Boutten, W. (2017. Etude par géolocalisation de l’utilisation de l’espace par le goéland d’Audouin reproducteur des ZPS du Golfe d’Ajaccio. CEN-Corse	Movebank study 'Ichthyaetus audouinii Corsica'. Accessed on [date].	CC_BY_NC

Source	Study ID	Species	Principal Investigator	Contact person	Citation	Recommended reference	License type
Movebank	295134472	Equus quagga	Chamaille-Jammes	Chamaille-Jammes		Movebank study 'Plains zebra Chamaill√©-Jammes Hwange NP'. Accessed on [date].	CC_BY_NC
Movebank	312267867	Larus marinus,Larus fuscus,Larus argentatus	Grigori Tertitski	Grigori Tertitski		Movebank study 'Solovki Larus Track'. Accessed on [date].	CC_0
Movebank	326568799	Gyps fulvus	Wolfgang Fiedler	Wolfgang Fiedler	Dieter Haas, Wolfgang Fiedler u.a.	Movebank study 'Griffon Vulture Albstadt Salzburg (Gypsi)'. Accessed on [date].	CC_BY
Movebank	358865092	Uria lomvia	Kyle Elliott	Allison Patterson		Movebank study 'Thick-billed murre Gilchrist and Elliott Digges 2015'. Accessed on [date].	CC_0
Movebank	384868221	Haliaeetus albicilla	Pawel; Mirski	Pawel; Mirski	Mirski P., unpubl. data.	Movebank study 'White-tailed Eagle Poland.'. Accessed on [date].	CC_BY_NC
Movebank	384882516	Aquila pomarina	Pawel; Mirski	Pawel; Mirski	Mirski P., unpubl.	Movebank study 'Spotted eagles NE Poland'. Accessed on [date].	CC_BY_NC
Movebank	439735878	Hypsignathus monstrosus	Sarah Olson	Sarah Olson	Olson SH, Bounga G, Ondzie A, Bushmaker T, Seifert SN, Kuisma E, et al. (2019) Lek-associated movement of a putative Ebolavirus reservoir, the hammer-headed fruit bat (Hypsignathus monstrosus), in northern Republic of Congo. PLoS ONE 14(10): e0223139. https://doi.org/10.1371/journal.pone.0223139	https://doi.org/10.1371/journal.pone.0223139	CC_BY
Movebank	467107447	Anser albifrons	Helmut Kruckenberg	Andrea Kölzsch	Kruckenberg H, Müskens GJDM, Ebbinge BS. 2018. Data from: A periodic Markov model to formalise animal migration on a network [white-fronted goose data]. Movebank Data Repository. https://www.doi.org/10.5441/001/1.kk38017f <br>Kölzsch A, Kleyheeg E, Kruckenberg H, Kaatz M, Blasius B. 2018. A periodic Markov model to formalise animal migration on a network. R Soc Open Sci. 5(6):180438. https://doi.org/10.1098/rsos.180438 <br>Kölzsch A, Müskens GJDM, Kruckenberg H, Glazov P, Weinzierl R, Nolet BA, Wikelski M. 2016. Towards a new understanding of migration timing: slower spring than autumn migration in geese reflects different decision rules for stopover use and departure. Oikos. 125(10):1496-1507. https://doi.org/10.1111/oik.03121 <br>Van Wijk RE, Kölzsch A, Kruckenberg H, Ebbinge BS, Müskens GJDM, Nolet BA. 2012. Individually tracked geese follow peaks of temperature acceleration during spring migration. Oikos. 121(5):655-664. https://doi.org/10.1111/j.1600-0706.2011.20083.x	https://doi.org/10.5441/001/1.kk38017f	CC_BY

Source	Study ID	Species	Principal Investigator	Contact person	Citation	Recommended reference	License type
Movebank	468460067	Nasua narica,Pecari tajacu,Ateles geoffroyi,Cebus capucinus,Potos flavus	Margaret Crofoot	Rasmus Havmoeller	Kays R, Hirsch B, Caillaud D, Mares R, Alavi S, Havmøller RW, Crofoot M. 2023. Data from: Multi-scale movement syndromes for comparative analyses of animal movement patterns. Movebank Data Repository. https://doi.org/10.5441/001/1.295 <br>These data are described in<br>Kays R, Hirsch B, Caillaud D, Mares R, Alavi S, Havmøller RW, Crofoot M. 2023. Multi-scale movement syndromes for comparative analyses of animal movement patterns. Move Ecol. 11:61. https://doi.org/10.1186/s40462-022-00365-y	https://doi.org/10.5441/001/1.295	CC_BY
Movebank	492444603	Canis lupus	Stan Boutin	Amanda Droghini	Bohm, H., A. Droghini, E. W. Neilson, and S. Boutin. 2018. Northern Alberta Grey Wolf, Study ID 492444603. Available on Movebank (movebank.org). Accessed on [access date].	https://doi.org/10.1371/journal.pone.0205742	CC_0
Movebank	496130871	Anthropoides virgo	Elena Ilyashenko	Ivan Pokrovskiy	Ilyashenko EI, Pokrovsky I, Ilyashenko VY, Mudrik EA, Korepov M, Mnatsekanov RA, Politov D, Fiedler W, Wikelski M. 2024. Data from: Study "1000 Cranes. Russia. Taman. Azov.". Movebank Data Repository. https://doi.org/10.5441/001/1.594 <br>These data are described in<br>Yanco SW, Oliver RY, Iannarilli F, Carlson BS, Heine G, Mueller U, Richter N, Vorneweg B, Andryushchenko Y, Batbayar N, et al. 2024. Migratory birds modulate niche tradeoffs in rhythm with seasons and life history. Proc Natl Acad Sci USA. https://doi.org/10.1073/pnas.2316827121	https://doi.org/10.5441/001/1.594	CC_BY
Movebank	496136836	Anthropoides virgo	Elena Ilyashenko	Ivan Pokrovskiy	Ilyashenko EI, Pokrovsky I, Ilyashenko VY, Mudrik EA, Korepov M, Politov D, Gugueva E, Fiedler W, Wikelski M. 2024. Data from: Study "1000 Cranes. Russia. Volga-Ural Interfluve.". Movebank Data Repository. https://doi.org/10.5441/001/1.595 <br>These data are described in<br>Yanco SW, Oliver RY, Iannarilli F, Carlson BS, Heine G, Mueller U, Richter N, Vorneweg B, Andryushchenko Y, Batbayar N, et al. 2024. Migratory birds modulate niche tradeoffs in rhythm with seasons and life history. Proc Natl Acad Sci USA. https://doi.org/10.1073/pnas.2316827121	https://doi.org/10.5441/001/1.595	CC_BY
Movebank	497087673	Anthropoides virgo	Elena Ilyashenko	Ivan Pokrovskiy	Ilyashenko EI, Pokrovsky I, Mudrik EA, Politov D, Postelnykh K, Ilyashenko VY, Fiedler W, Wikelski M. 2024. Data from: Study "1000 Cranes. Russia. Altai.". Movebank Data Repository. https://doi.org/10.5441/001/1.596 <br>These data are described in<br>Yanco SW, Oliver RY, Iannarilli F, Carlson BS, Heine G, Mueller U, Richter N, Vorneweg B, Andryushchenko Y, Batbayar N, et al. 2024. Migratory birds modulate niche tradeoffs in rhythm with seasons and life history. Proc Natl Acad Sci USA. https://doi.org/10.1073/pnas.2316827121	https://doi.org/10.5441/001/1.596	CC_BY

Source	Study ID	Species	Principal Investigator	Contact person	Citation	Recommended reference	License type
Movebank	497091077	Grus vipio,Anthropoides virgo	Elena Ilyashenko	Ivan Pokrovskiy	Ilyashenko EI, Pokrovsky I, Goroshko OA, Mudrik EA, Politov D, Ilyashenko VY, Fiedler W, Wikelski M. 2024. Data from: Study "1000 Cranes. Russia. Transbaikalia.". Movebank Data Repository. https://doi.org/10.5441/001/1.592 <br>These data are described in<br>Yanco SW, Oliver RY, Iannarilli F, Carlson BS, Heine G, Mueller U, Richter N, Vorneweg B, Andryushchenko Y, Batbayar N, et al. 2024. Migratory birds modulate niche tradeoffs in rhythm with seasons and life history. Proc Natl Acad Sci USA. https://doi.org/10.1073/pnas.2316827121	https://doi.org/10.5441/001/1.592	CC_BY
Movebank	497138661	Anthropoides virgo	Elena Ilyashenko	Ivan Pokrovskiy	Ilyashenko EI, Pokrovsky I, Gavrilov AE, Zaripova S, Fiedler W, Wikelski M. 2024. Data from: Study "1000 Cranes. Southern Kazakhstan.". Movebank Data Repository. https://doi.org/10.5441/001/1.591 <br>These data are described in<br>Yanco SW, Oliver RY, Iannarilli F, Carlson BS, Heine G, Mueller U, Richter N, Vorneweg B, Andryushchenko Y, Batbayar N, et al. 2024. Migratory birds modulate niche tradeoffs in rhythm with seasons and life history. Proc Natl Acad Sci USA. https://doi.org/10.1073/pnas.2316827121	https://doi.org/10.5441/001/1.591	CC_BY
Movebank	505156776	Anser anser	Harald Grabenhofer	Harald Grabenhofer	Grabenhofer, H. et al, unpublished Data	Movebank study 'Graugans Zugverhalten Neusiedler See'. Accessed on [date].	CC_BY_NC
Movebank	542653863	Grus grus	Elena Ilyashenko	Ivan Pokrovskiy	Ilyashenko EI, Pokrovsky I, Ilyashenko VY, Korepov M, Fiedler W, Wikelski M. 2024. Data from: Study "1000 Cranes. Russia. Common Crane.". Movebank Data Repository. https://doi.org/10.5441/001/1.593 <br>These data are described in<br>Yanco SW, Oliver RY, Iannarilli F, Carlson BS, Heine G, Mueller U, Richter N, Vorneweg B, Andryushchenko Y, Batbayar N, et al. 2024. Migratory birds modulate niche tradeoffs in rhythm with seasons and life history. Proc Natl Acad Sci USA. https://doi.org/10.1073/pnas.2316827121	https://doi.org/10.5441/001/1.593	CC_BY
Movebank	560041066	Ciconia ciconia	Ran Nathan	Shay Rotics	Rotics S, Kaatz M, Turjeman S, Zurell D, Wikelski M, Sapir N, Eggers U, Fiedler W, Jeltsch F, Nathan R. 2018. Data from: Early arrival at breeding grounds: causes, costs and a trade-off with overwintering latitude. Movebank Data Repository. https://www.doi.org/10.5441/001/1.v8d24552 <br>Rotics S, Kaatz M, Turjeman S, Zurell D, Wikelski M, Sapir N, Eggers U, Fiedler W, Jeltsch F, Nathan R. 2018. Early arrival at breeding grounds: causes, costs and a trade-off with overwintering latitude. J Anim Ecol. 87(6):1627-1638. https://doi.org/10.1111/1365-2656.12898	https://doi.org/10.5441/001/1.v8d24552	CC_BY
Movebank	581719332	Pelecanus occidentalis	Jordan Karubian	Jordan Karubian		Movebank study 'Brown Pelican Raccoon Island LA 2014-2017'. Accessed on [date].	CC_BY_NC

Source	Study ID	Species	Principal Investigator	Contact person	Citation	Recommended reference	License type
Movebank	604806671	Circus aeruginosus	Anny Anselin	Tanja Milotic	Anselin A, Desmet P, Milotic T, Janssens K, T'Jollyn F, De Bruyn L, Bouten W (2019) MH_WATERLAND - Western marsh harriers (Circus aeruginosus, Accipitridae) breeding near the Belgium-Netherlands border. Dataset. https://doi.org/10.5281/zenodo.3532940	https://doi.org/10.1007/s10336-020-01785-6	CC_0
Movebank	631036041	Anthropoides virgo	Nyambayar Batbayar	Nyambayar Batbayar	Batbayar N, Galtbalt B, Natsagdorj T, Sukhbaatar T, Wikelski M. 2024. Data from: Study "1000 Cranes. Mongolia." Movebank Data Repository. https://doi.org/10.5441/001/1.598 <br>These data are described in<br>Yanco SW, Oliver RY, Iannarilli F, Carlson BS, Heine G, Mueller U, Richter N, Vorneweg B, Andryushchenko Y, Batbayar N, et al. 2024. Migratory birds modulate niche tradeoffs in rhythm with seasons and life history. Proc Natl Acad Sci USA. https://doi.org/10.1073/pnas.2316827121	https://doi.org/10.5441/001/1.598	CC_BY_NC
Movebank	657674643	Anser albifrons	Andrea Kölzsch	Andrea Kölzsch	Kölzsch A, Müskens GJDM, Moonen S, Kruckenberg H, Glazov P, Wikelski M. 2019. Data from: Flyway connectivity and exchange primarily driven by moult migration in geese [North Sea population]. Movebank Data Repository. https://www.doi.org/10.5441/001/1.ct72m82n <br>Kölzsch A, Müskens GJDM, Szinai P, Moonen S, Glazov P, Kruckenberg H, Wikelski M, Nolet BA. 2019. Flyway connectivity and exchange primarily driven by moult migration in geese. Move Ecol. 7:3. https://doi.org/10.1186/s40462-019-0148-6	https://doi.org/10.5441/001/1.ct72m82n	CC_BY
Movebank	657965212	Anser albifrons	Andrea Kölzsch	Andrea Kölzsch	Müskens GJDM, Szinai P, Sapi T, Kölzsch A, Wikelski M, Nolet BA. 2019. Data from: Flyway connectivity and exchange primarily driven by moult migration in geese [Pannonic population]. Movebank Data Repository. https://www.doi.org/10.5441/001/1.46b0mq21 <br>Kölzsch A, Müskens GJDM, Szinai P, Moonen S, Glazov P, Kruckenberg H, Wikelski M, Nolet BA. 2019. Flyway connectivity and exchange primarily driven by moult migration in geese. Move Ecol. 7:3. https://doi.org/10.1186/s40462-019-0148-6	https://doi.org/10.5441/001/1.46b0mq21	CC_BY
Movebank	672882373	Milvus milvus	Patrick Scherler	Patrick Scherler		Movebank study 'Milvus_milvus_atlantismarcuard'. Accessed on [date].	CC_BY_NC
Movebank	673728219	Ovis dalli	James P Lawler	James P Lawler	Lawler JP, Griffith B, Johnson D, Burch J. 2005. The effects of military jet overflights on Dall's Sheep in Interior Alaska. Report to the Department of the Air Force. National Park Service, Alaska Region Natural Resource Technical Report NPS/AR/NRTR-2005-51, 181 p.	https://doi.org/http://purl.access.gpo.gov/GPO/LPS64886	CC_BY

Source	Study ID	Species	Principal Investigator	Contact person	Citation	Recommended reference	License type
Movebank	681056756	Pteropus lylei	Julien Cappelle	Julien Cappelle	Choden K, Ravon S, Epstein JH, Hoem T, Furey N, Gely M, Jolivot A, Hul V, Neung C, Tran A, Cappelle J (2020) Data from: Pteropus lylei primarily forages in residential areas in Kandal, Cambodia. Movebank Data Repository. https://www.doi.org/10.5441/001/1.j25661td <br>Choden K, Ravon S, Epstein JH, Hoem T, Furey N, Gely M, Jolivot A, Hul V, Neung C, Tran A, Cappelle J (2019) Pteropus lylei primarily forages in residential areas in Kandal, Cambodia. Ecology and Evolution 9(7): 4181–4191. https://doi.org/10.1002/ece3.5046	https://doi.org/10.5441/001/1.j25661td	CC_0
Movebank	736029750	Loxodonta africana	Abi Tamim Vanak	Abi Tamim Vanak	Slotow R, Thaker M, Vanak AT (2019) Data from: Fine-scale tracking of ambient temperature and movement reveals shuttling behavior of elephants to water. Movebank Data Repository. https://www.doi.org/10.5441/001/1.403h24q5 <br>Thaker M, Gupte PR, Prins HHT, Slotow R, Vanak AT. 2019. Fine-scale tracking of ambient temperature and movement reveals shuttling behavior of elephants to water. Front Ecol Evol. 7:4. https://doi.org/10.3389/fevo.2019.00004	https://doi.org/10.5441/001/1.403h24q5	CC_BY_NC

Source	Study ID	Species	Principal Investigator	Contact person	Citation	Recommended reference	License type
Movebank	897981076	Cervus elaphus	Mark Hebblewhite	Mark Hebblewhite	Data for 2001-2020 are published as<br>Hebblewhite M, Merrill EH, Martin H, Berg JE, Bohm H, Eggeman SL. 2020. Data from: Study "Ya Ha Tinda elk project, Banff National Park, 2001-2020 (females)". Movebank Data Repository. https://www.doi.org/10.5441/001/1.5g4h5t6c <br>Tucker MA, Böhning-Gaese K, Fagan WF, Fryxell JM, Van Moorter B, Alberts SC, Ali AH, Allen AM, Attias N, Avgar T, et al. 2018. Moving in the Anthropocene: global reductions in terrestrial mammalian movements. Science 359(6374):466–469. https://www.doi.org/10.1126/science.aam9712 <br>Eggeman S, Hebblewhite M, Bohm H, Whittington J, Merrill E. 2016. Behavioral flexibility in migratory behavior in a long-lived large herbivore. J Anim Ecol 85(3):785-797. https://www.doi.org/10.1111/1365-2656.12495 <br>Hebblewhite M, Merrill EH, Morgantini LE, White CA, Allen JR, Bruns E, Thurston L, Hurd TE. 2006. Is the migratory behaviour of montane elk herds in peril? The case of Alberta's Ya Ha Tinda elk herd. Wildlife Soc B 34(5):1280-1294. https://www.doi.org/10.2193/0091-7648(2006)34[1280:ITMBOM]2.0.CO;2 <br>Hebblewhite M, Merrill EH. 2009. Trade-offs between predation risk and forage differ between migrant strategies in a migratory ungulate. Ecology 90(12):3445-3454. https://www.doi.org/10.1890/08-2090.1 <br>Hebblewhite M, Merrill EH. 2008. Modelling wildlife-human relationships for social species with mixed-effects resource selection models. J Appl Ecol 45(3):834-844. https://www.doi.org/10.1111/j.1365-2664.2008.01466.x <br>Hebblewhite M, Merrill EH, McDermid G. 2008. A multi-scale test of the forage maturation hypothesis in a partially migratory ungulate population. Ecol Monogr 78(2):141-166. https://www.doi.org/10.1890/06-1708.1 <br>Hebblewhite M, Merrill EH. 2007. Multiscale wolf predation risk for elk: does migration reduce risk? Oecologia 152(2):377-387. https://www.doi.org/10.1007/s00442-007-0661-y <br>Hebblewhite M. 2006. Linking predation risk and forage to ungulate population dynamics [dissertation]. Edmonton, Alberta: University of Alberta.	https://doi.org/10.5441/001/1.5g4h5t6c	CC_0
Movebank	909521569	Pernis apivorus	Patrik Byholm	Elham Nourani	Nourani E, Vansteelant WMG, Byholm P, Safi K. 2020 Dynamics of the energy seascape can explain intra-specific variations in sea-crossing behaviour of soaring birds. Biol. Lett. 16: 20190797. http://dx.doi.org/10.1098/rsbl.2019.0797	https://doi.org/10.1098/rsbl.2019.0797	CC_BY_NC

Source	Study ID	Species	Principal Investigator	Contact person	Citation	Recommended reference	License type
Movebank	914913822	Pelecanus onocrotalus	Ran Nathan	Ron Efrat	Efrat R, Hatzofe O, Nathan R. 2019. Data from: Landscape-dependent time versus energy optimisations in pelicans migrating through a large ecological barrier. Movebank Data Repository. https://www.doi.org/10.5441/001/1.hs79pk45 <br>Efrat R, Hatzofe O, Nathan R. 2019. Landscape-dependent time versus energy optimisations in pelicans migrating through a large ecological barrier. Funct Ecol. 33(11):2161-2171. https://doi.org/10.1111/1365-2435.13426	https://doi.org/10.5441/001/1.hs79pk45	CC_0
Movebank	922263102	Circus aeruginosus	Almut Schlaich & Tonio Schaub	Tanja Milotic	Koks B, Schlaich A, Schaub T, Klaassen R, Anselin A, Desmet P, Milotic T, Janssens K, Bouten W (2019) H_GRONINGEN - Western marsh harriers (Circus aeruginosus, Accipitridae) breeding in Groningen (the Netherlands). Dataset. https://doi.org/10.5281/zenodo.3552507	https://doi.org/10.3897/zookeys.947.52570	CC_0
Movebank	924418031	Gallinago gallinago,Larus canus	Marcin	Marcin	www.interrex-tracking.com	Movebank study 'Common Snipe GPS-GSM logger Poland-Russia'. Accessed on [date].	CC_0

Source	Study ID	Species	Principal Investigator	Contact person	Citation	Recommended reference	License type
Movebank	933711994	Cervus elaphus	Mark S. Boyce	Sarah Davidson	Boyce MS and Ciuti S (2020) Data from: Human selection of elk behavioural traits in a landscape of fear. Movebank Data Repository. https://www.doi.org/10.5441/001/1.j484vk24 <br>Paton DG, Ciuti S, Quinn M, Boyce MS (2017) Hunting exacerbates the response to human disturbance in large herbivores while migrating through a road network. Ecosphere 8(6): e01841. https://doi.org/10.1002/ecs2.1841 <br>Prokopenko CM, Boyce MS, Avgar T (2017) Characterizing wildlife behavioural responses to roads using integrated step selection analysis. Journal of Applied Ecology 54: 470-479. https://doi.org/10.1111/1365-2664.12768 <br>Prokopenko CM, Boyce MS, Avgar T (2017) Extent-dependent habitat selection in a migratory large herbivore: road avoidance across scales. Landscape Ecology 32: 313-325. https://doi.org/10.1007/s10980-016-0451-1 <br>Roberts DR, Bahn V, Ciuti S, Boyce MS, Elith J, Guillera-Arroita G, Hauenstein S, Lahoz-Monfort JJ, Schöder B, Thuiller W, Warton DI, Wintle BA, Hartig F, Dormann CF (2017) Cross-validation strategies for data with temporal, spatial, hierarchical, or phylogenetic structure. Ecography 40: 913-929. https://doi.org/10.1111/ecog.02881 <br>Thurfjell H, Ciuti S, Boyce MS (2017) Learning from the mistakes of others: How female elk (Cervus elaphus) adjust behaviour with age to avoid hunters. PLoS ONE 12(6): e0178082. https://doi.org/10.1371/journal.pone.0178082 <br>Benz RA, Boyce MS, Thurfjell H, Paton DG, Musiani M, Dormann CF, et al. (2016) Dispersal ecology informs design of large-scale wildlife corridors. PLoS ONE 11(9): e0162989. https://doi.org/10.1371/journal.pone.0162989 <br>Ensing EP, Ciuti S, de Wijs FALM, Lentferink DH, ten Hoedt A, Boyce MS, Hut RA (2014) GPS based daily activity patterns in European red deer and North American elk (Cervus elaphus): indication for a weak circadian clock in ungulates. PLoS ONE 9(9): e106997. https://doi.org/10.1371/journal.pone.0106997 <br>Killeen J, Thurfjell H, Ciuti S, Paton D, Musiani M, Boyce MS (2014) Habitat selection during ungulate dispersal and exploratory movement at broad and fine scale with implications for conservation management. Movement Ecology 2:15. https://doi.org/10.1186/s40462-014-0015-4 <br>Thurfjell H, Ciuti S, Boyce MS (2014) Applications of step-	https://doi.org/10.5441/001/1.j484vk24	CC_BY
Movebank	938783961	Buteo buteo,Circus aeruginosus	Geert Spanoghe	Tanja Milotic	Spanoghe G, Desmet P, Milotic T, Janssens K, De Regge N, Vanoverbeke J, Bouten W (2019) MH_ANTWERPEN - Western marsh harriers (Circus aeruginosus, Accipitridae) breeding near Antwerp (Belgium). Dataset. https://doi.org/10.5281/zenodo.3550093	https://doi.org/10.3897/zookeys.947.52570	CC_0

Source	Study ID	Species	Principal Investigator	Contact person	Citation	Recommended reference	License type
Movebank	943824007	Balaenoptera physalus,Balaenoptera musculus	Daniel Palacios	Barb Lagerquist	Irvine LM, Palacios DM, Lagerquist BA, Mate BR, Follett TM. 2019. Data from: Scales of blue and fin whale feeding behavior off California, USA, with implications for prey patchiness. Movebank Data Repository. https://www.doi.org/10.5441/001/1.47h576f2 <br>Irvine LM, Palacios DM, Lagerquist BA, Mate BR. 2019. Scales of blue and fin whale feeding behavior off California, USA, with implications for prey patchiness. Front Ecol Evol. 7:338. https://doi.org/10.3389/fevo.2019.00338 <br>Irvine LM, Winsor MH, Follett TM, Mate BR, Palacios DM. 2020. An at-sea assessment of Argos location accuracy for three species of large whales, and the effect of deep-diving behavior on location error. Animal Biotelemetry. 8:20. https://doi.org/10.1186/s40317-020-00207-x	https://doi.org/10.5441/001/1.47h576f2	CC_BY
Movebank	985143423	Larus fuscus	Eric Stienen	Peter Desmet	Stienen EWM, Desmet P, Milotic T, Hernandez F, Deneudt K, Bouten W, Müller W, Matheve H, Lens L (2019) LBBG_ZEEBRUGGE - Lesser black-backed gulls (Larus fuscus, Laridae) breeding at the southern North Sea coast (Belgium and the Netherlands). Dataset. https://doi.org/10.5281/zenodo.3540799	https://doi.org/10.5281/zenodo.3540799	CC_0
Movebank	986040562	Larus argentatus	Eric Stienen	Peter Desmet	Stienen EWM, Desmet P, Milotic T, Hernandez F, Deneudt K, Matheve H, Bouten W (2019) HG_OOSTENDE - Herring gulls (Larus argentatus, Laridae) breeding at the southern North Sea coast (Belgium). Dataset. https://doi.org/10.5281/zenodo.3541811	https://doi.org/10.5281/zenodo.3541811	CC_0
Movebank	1007794443	Larus marinus	Stefan Garthe	Stefan Garthe	Borrmann RM, Phillips RA, Clay TA, Garthe S. 2019. Data from: High foraging site fidelity and spatial segregation among individual great black-backed gulls. Movebank Data Repository. https://www.doi.org/10.5441/001/1.ht5jf68h <br>Borrmann RM, Phillips RA, Clay TA, Garthe S. 2019. High foraging site fidelity and spatial segregation among individual great black-backed gulls. J Avian Biol. 50(12). https://doi.org/10.1111/jav.02156	https://doi.org/10.5441/001/1.ht5jf68h	CC_BY_NC

Source	Study ID	Species	Principal Investigator	Contact person	Citation	Recommended reference	License type
Movebank	1030734949	Cygnus columbianus	Rascha Nuijten	Rascha Nuijten	Nuijten RJM, Gerrits T, de Vries PP, Müskens GJDM, Nolet BA. 2020. Data from: Less is more: on-board lossy compression of accelerometer data increases biologging capacity. Movebank Data Repository. https://www.doi.org/10.5441/001/1.8ms7mm80 <br>Nuijten RJM, Gerrits T, Shamoun-Baranes J, Nolet BA. 2020. Less is more: on-board lossy compression of accelerometer data increases biologging capacity. J Anim Ecol. 89(1):237-247. https://www.doi.org/10.1111/1365-2656.13164	https://doi.org/10.5441/001/1.8ms7mm80	CC_0
Movebank	1049685237	Anser albifrons	Andrea Kölzsch	Andrea Kölzsch	Kölzsch A, Müskens GJDM, Glazov P, Kruckenberg H, Wikelski M. 2020. Data from: Goose parents lead migration V. Movebank Data Repository. https://www.doi.org/10.5441/001/1.ms87s2m6<br><br>Kölzsch A, Flack A, Müskens GJDM, Kruckenberg H, Glazov P, Wikelski M. 2020. Goose parents lead migration V. J Avian Biol. 51(3). https://doi.org/10.1111/jav.02392	https://doi.org/10.5441/001/1.ms87s2m6	CC_0
Movebank	1071101052	Larus argentatus	Robert Ronconi	Robert Ronconi	Ronconi RA, Shlepr KR. 2020. Data from: Study "Herring Gulls (Larus Argentatus); Ronconi; Kent Island, Canada". Movebank Data Repository. https://www.doi.org/10.5441/001/1.t5n4s456 <br>Anderson CM, Gilchrist HG, Ronconi RA, Shlepr KR, Clark DE, Fifield DA, Robertson GJ, Mallory ML. 2020. Both short and long distance migrants use energy-minimizing strategies in North American herring gulls. Movement Ecology. 8:26. https://doi.org/10.1186/s40462-020-00207-9	https://doi.org/10.5441/001/1.t5n4s456	CC_0
Movebank	1071134107	Larus argentatus	Robert Ronconi	Robert Ronconi	Ronconi RA, Shlepr KR. 2020. Data from: Study "Herring Gulls (Larus Argentatus); Ronconi; Brier Island, Canada". Movebank Data Repository. https://www.doi.org/10.5441/001/1.282vr7kd <br>Anderson CM, Gilchrist HG, Ronconi RA, Shlepr KR, Clark DE, Fifield DA, Robertson GJ, Mallory ML. 2020. Both short and long distance migrants use energy-minimizing strategies in North American herring gulls. Movement Ecology. 8:26. https://doi.org/10.1186/s40462-020-00207-9	https://doi.org/10.5441/001/1.282vr7kd	CC_0
Movebank	1073231887	Buteo jamaicensis	Pete Bloom	Pete Bloom	Bloom PH (2015) Northward summer migration of red-tailed hawks fledged from southern latitudes. Journal of Raptor Research 49(1): 1-17. https://doi.org/10.3356/jrr-14-54.1 <br>Bloom PH (2011) Vagrancy, natal dispersal and migrations of red-tailed and red-shouldered hawks banded in the Pacific Flyway. Ph.D. dissertation, University of Idaho, Moscow, ID USA.	https://doi.org/10.3356/jrr-14-54.1	CC_BY

Source	Study ID	Species	Principal Investigator	Contact person	Citation	Recommended reference	License type
Movebank	1077432441	Pteropus poliocephalus	Wayne Boardman	David Roshier	Roshier D, Boardman WSJ. 2021. Data from: Spring foraging movements of an urban population of grey-headed flying foxes (Pteropus poliocephalus). Movebank Data Repository. https://www.doi.org/10.5441/001/1.5bd6pq55 <br>Boardman WSJ, Roshier D, Reardon T, Burbidge K, McKeown A, Westcott DA, Caraguel CGB, Prowse TAA. 2021. Spring foraging movements of an urban population of grey-headed flying foxes (Pteropus poliocephalus). J Urban Ecol. 7(1):juaa034. https://doi.org/10.1093/jue/juaa034	https://doi.org/10.5441/001/1.5bd6pq55	CC_0
Movebank	1080341217	Larus argentatus	Dan Clark	Christine Anderson	Clark DE, Mackenzie SA, Koenen K, Whitney J, DeStefano S. 2020. Data from: Study "Herring Gulls (Larus Argentatus); Clark; Massachussets, United States". Movebank Data Repository. https://www.doi.org/10.5441/001/1.3th8b5q3 <br>Anderson CM, Gilchrist HG, Ronconi RA, Shlepr KR, Clark DE, Fifield DA, Robertson GJ, Mallory ML. 2020. Both short and long distance migrants use energy-minimizing strategies in North American herring gulls. Movement Ecology. 8:26. https://doi.org/10.1186/s40462-020-00207-9	https://doi.org/10.5441/001/1.3th8b5q3	CC_0
Movebank	1080341737	Larus argentatus	Robert Ronconi	Robert Ronconi	Ronconi RA, Taylor PD. 2020. Data from: Study "Herring Gulls (Larus Argentatus); Ronconi; Sable Island, Canada". Movebank Data Repository. https://www.doi.org/10.5441/001/1.3264ss3v <br>Anderson CM, Gilchrist HG, Ronconi RA, Shlepr KR, Clark DE, Fifield DA, Robertson GJ, Mallory ML. 2020. Both short and long distance migrants use energy-minimizing strategies in North American herring gulls. Movement Ecology. 8:26. https://doi.org/10.1186/s40462-020-00207-9	https://doi.org/10.5441/001/1.3264ss3v	CC_0
Movebank	1087068449	Tockus deckeni	Martin Wikelski	Katherine Mertes	Mertes K, Jetz W, Wikelski M (2020) Data from: Hierarchical multi-grain models improve descriptions of species' environmental associations, distribution, and abundance. Movebank Data Repository. https://www.doi.org/10.5441/001/1.cp97k9j1 <br>Schwartz K, Jarzyna M, Jetz W (2020) Hierarchical multi-grain models improve descriptions of species' environmental associations, distribution, and abundance. Ecological Applications. https://doi.org/10.1002/eap.2117	https://doi.org/10.5441/001/1.cp97k9j1	CC_0
Movebank	1088836380	Lynx rufus,Pekania pennanti	Scott LaPoint	Scott LaPoint	LaPoint et al. in progress	Movebank study 'Carnivore movements near Black Rock Forest New York'. Accessed on [date].	CC_BY

Source	Study ID	Species	Principal Investigator	Contact person	Citation	Recommended reference	License type
Movebank	1091848505	Larus ,Larus hyperboreus,Larus glaucescens,Larus smithsonianus	Andrew Ramey	Andrew Ramey	</br>Ramey, A.M., Ahlstrom, C.A., van Toor, M.L., Woksepp, H., Chandler, J.C., Reed, J.A., Reeves, A.B., Waldenström, J., Franklin, A.B., Bonnedahl, J., and Douglas, D.C. 2020, Tracking data for three large-bodied gull species and hybrids (Larus spp.) (ver 1.0, June 2020): U.S. Geological Survey data release, doi:10.5066/P9FZ4OJW</br>	https://doi.org/10.1016/j.scitotenv.2020.144551	CC_0
Movebank	1099562810	Haematopus ostralegus	Geert Spanoghe	Tanja Milotic	Spanoghe G, Desmet P, Milotic T, Van Ryckegem G, Vanoverbeke J, Ens BJ, Bouten W (2020) O_WESTERSCHELDE - Eurasian oystercatchers (Haematopus ostralegus, Haematopodidae) breeding in East Flanders (Belgium). Dataset. https://doi.org/10.5281/zenodo.3734898	https://doi.org/10.3897/zookeys.1123.90623	CC_0
Movebank	1120749252	Nasua narica,Pecari tajacu,Ateles geoffroyi,Cebus capucinus,Potos flavus	Meg Crofoot	Meg Crofoot	Kays R, Hirsch B, Caillaud D, Mares R, Alavi S, Havmøller RW, Crofoot M. 2023. Data from: Multi-scale movement syndromes for comparative analyses of animal movement patterns. Movebank Data Repository. https://doi.org/10.5441/001/1.295 <br>These data are described in<br>Kays R, Hirsch B, Caillaud D, Mares R, Alavi S, Havmøller RW, Crofoot M. 2023. Multi-scale movement syndromes for comparative analyses of animal movement patterns. Move Ecol. 11:61. https://doi.org/10.1186/s40462-022-00365-y	https://doi.org/10.5441/001/1.295	CC_BY
Movebank	1123149708	Pagophila eburnea	Morten Frederiksen	Morten Frederiksen	Frederiksen, M. (2019) GPS tracking of breeding ivory gulls in NE Greenland 2018-2019	Movebank study 'Ivory gull N Greenland 2018/19'. Accessed on [date].	CC_BY_NC
Movebank	1207235729	Canis latrans	Nucharin Songsasen, PhD, DVM	Jared Stabach		Movebank study 'Coyote (Canis latrans) movement in the northern blue ridge mountains - Virginia'. Accessed on [date].	CC_0
Movebank	1208105916	Larus cachinnans	INTERREX	INTERREX		Movebank study 'Caspian Gulls - Poland'. Accessed on [date].	CC_0
Movebank	1229945587	Grus grus	Petras Kurlavičius	Aleksej Perzhu	Unpublished & ongoing study by Lithuanian University of Educational Sciences (LEU)	Movebank study 'Common Crane 2020 (Lithuanian University of Educational Studies; LEU)'. Accessed on [date].	CC_0

Source	Study ID	Species	Principal Investigator	Contact person	Citation	Recommended reference	License type
Movebank	1241071371	Vulpes lagopus	Dominique Berteaux	Dominique Berteaux	Clermont J., Grenier-Potvin A., Duchesne É., Couchoux C., Dulude-de Broin F., Beardsell A., Bêty J., Berteaux D. 2021. The predator activity landscape predicts the anti-predator behavior and distribution of prey in a tundra community. Ecosphere 12(12):e03858. https://doi.org/10.1002/ecs2.3858<br><br>Clermont J., Woodward-Gagné S., Berteaux D. 2021. Digging into the behaviour of an active hunting predator: arctic fox prey caching events revealed by accelerometry. Mov Ecol 9:58. https://doi.org/10.1186/s40462-021-00295-1<br><br>Poulin M-P, Clermont J, Berteaux D. 2021. Extensive daily movement rates measured in territorial arctic foxes. Ecol Evol. 11(6):2503-14. https://www.doi.org/ 10.1002/ece3.7165<br><br>Grenier-Potvin A, Clermont J, Gauthier G, Berteaux D. 2021. Prey and habitat distribution are not enough to explain predator habitat selection: addressing intraspecific interactions, behavioural state and time. Mov Ecol. 9:12. https://www.doi.org/10.1186/s40462-021-00250-0 	https://doi.org/10.1002/ece3.7165	CC_0
Movebank	1252230318	Ciconia ciconia	Marcin Tobółka	Marcin Tobółka	Tobółka, M., Nowak, B., Białas, J.T., Jagiełło, Z., Zielińska, Z. 2020. The influence of parental experience and conditions during breeding and non-breeding period on reproductive success of long-lived birds, an example of the White Stork.	Movebank study 'White stork Ciconidae Western Poland'. Accessed on [date].	CC_0
Movebank	1258895879	Larus fuscus,Larus argentatus	Eric Stienen	Peter Desmet	Stienen EWM, Buijs R-J, de Visser J, Fijn R, Lilipaly S, Platteeuw M, Desmet P (2023) DELTATRACK - Herring gulls (Larus argentatus, Laridae) and lesser black-backed gulls (Larus fuscus, Laridae) breeding at Neeltje Jans (Netherlands). Dataset. https://doi.org/10.5281/zenodo.10209520	https://doi.org/10.5281/zenodo.10209520	CC_0
Movebank	1259686571	Larus fuscus,Larus argentatus	Eric Stienen	Peter Desmet	Stienen EWM, Müller W, Lens L, Milotic T, Desmet P (2021) LBBG_JUVENILE - Juvenile lesser black-backed gulls (Larus fuscus, Laridae) and herring gulls (Larus argentatus, Laridae) hatched in Zeebrugge (Belgium). Dataset. https://doi.org/10.5281/zenodo.5075868	https://doi.org/10.5281/zenodo.5075868	CC_0
Movebank	1266784970	Corvus corone	JIGUET Frédéric	Jiguet Frédéric		Movebank study 'Corvus corone [ID_PROG 883]'. Accessed on [date].	CC_BY_NC

Source	Study ID	Species	Principal Investigator	Contact person	Citation	Recommended reference	License type
Movebank	1278021460	Buteo buteo,Circus cyaneus,Circus pygargus,Asio flammeus,Circus aeruginosus	Geert Spanoghe	Peter Desmet	Spanoghe G, Janssens K, Klaassen R, Schaub T, Milotic T, Desmet P (2021) BOP_RODENT - Rodent specialized birds of prey (Circus, Asio, Buteo) in Flanders (Belgium). Dataset. https://doi.org/10.5281/zenodo.5735405	https://doi.org/10.5281/zenodo.5735405	CC_0
Movebank	1326534946	Rissa ,Rissa tridactyla	Kyle Elliott	Kyle Elliott		Movebank study 'Black-legged Kittiwake Rissa tridactyla Middleton Island'. Accessed on [date].	CC_BY
Movebank	1375204238	Pygoscelis adeliae	Yan Ropert-Coudert	Candice Michelot	Michelot et al (Accepted in Plos One) Adélie penguins foraging consistency and site fidelity are conditioned by breeding status and environmental conditions	https://doi.org/10.1371/journal.pone.0244298	CC_BY_NC
Movebank	1393954358	Cathartes aura	Martin Wikelski	Kamran Safi		Movebank study 'Cathartes aura MPIAB Cuba'. Accessed on [date].	CC_BY_NC
Movebank	1395952585	Numenius arquata	Philipp Schwemmer	Philipp Schwemmer	Schwemmer P, Garthe S. 2021. Data from: Migrating curlews on schedule: departure and arrival patterns of a long-distance migrant depend on time and breeding location rather than on wind conditions. Movebank Data Repository. https://www.doi.org/10.5441/001/1.715k46g2 <br>Schwemmer P, Mercker M, Vanselow KH, Bocher P, Garthe S. 2021. Migrating curlews on schedule: departure and arrival patterns of a long-distance migrant depend on time and breeding location rather than on wind conditions. Movement Ecology. https://doi.org/10.1186/s40462-021-00252-y	https://doi.org/10.5441/001/1.715k46g2	CC_0
Movebank	1404033416	Anas rubripes	Chris Williams	Amanda Hoyt		Movebank study 'NC Breeding ABDU'. Accessed on [date].	CC_0
Movebank	1406291704	Panthera onca	Ronaldo Morato	Ronaldo Morato	Morato, RG; Tortato, F.	Movebank study 'Jaguar Rescue'. Accessed on [date].	CC_BY
Movebank	1410035327	Ciconia ciconia	Ben Carlson	Ben Carlson	Carlson BS, Rotics S, Nathan R, Wikelski M, Jetz W. 2021. Individual environmental niches in mobile organisms. Nat Commun. 12:4572. doi: 10.1038/s41467-021-24826-x	https://doi.org/10.5441/001/1.rj21g1p1	CC_0
Movebank	1415844328	Anser fabalis	Antti Piironen	Antti Piironen	Piironen A, Paasivaara A, Laaksonen T. 2021. Data from: Birds of three worlds: moult migration to high Arctic expands a boreal-temperate flyway to a third biome. Movebank Data Repository. https://www.doi.org/10.5441/001/1.22kk5126 <br>Piironen A, Paasivaara A, Laaksonen T. 2021. Birds of three worlds: moult migration to high Arctic expands a boreal-temperate flyway to a third biome. Movement Ecology. 9:47. https://www.doi.org/10.1186/s40462-021-00284-4	https://doi.org/10.5441/001/1.22kk5126	CC_BY

Source	Study ID	Species	Principal Investigator	Contact person	Citation	Recommended reference	License type
Movebank	1426277950	Tyto furcata	Matthew Johnson	Allison Huysman	Huysman AE, Castañeda XA, Johnson MD. 2021. Data from: Habitat selection by a predator of rodent pests is resilient to wildfire in a vineyard agroecosystem. Movebank Data Repository. https://www.doi.org/10.5441/001/1.82b5h1rk <br>Huysman AE, Johnson MD. 2021. Habitat selection by a predator of rodent pests is resilient to wildfire in a vineyard agroecosystem. Ecol Evol. https://doi.org/10.1002/ece3.8416 <br>Castañeda XA, Huysman AE, Johnson MD. 2021. Barn Owls select uncultivated habitats for hunting in a winegrape growing region of California. Ornithol Appl. 123(1):duaa058. https://doi.org/10.1093/ornithapp/duaa058	https://doi.org/10.5441/001/1.82b5h1rk	CC_BY
Movebank	1429664821	Connochaetes taurinus	Tom Morrison	Tom Morrison	Morrison TA, Link W, Newmark W, Foley C, Bolger DT. 2016. Tarangire revisited: population consequences of loss of migratory connectivity in a tropical ungulate. Biological Conservation. 197, 53-60.	https://doi.org/10.1016/j.biocon.2016.02.034	CC_BY_NC
Movebank	1445058753	Larus fuscus	Marcin	Marcin	www.interrex-tracking.com	Movebank study 'Larus heuglini_1'. Accessed on [date].	CC_0
Movebank	1448377103	Mycteria americana	Mathieu Basille	Mathieu Basille	Basille M, Borkhataria RR, Bryan AL, Bucklin DN, Picardi S, Frederick PC. 2021. Data from: Study "Wood stork (Mycteria americana) Southeastern US 2004–2019". Movebank Data Repository. DOI: 10.5441/001/1.r0h6725k<br><br>Picardi S, Frederick PC, Borkhataria RR, Basille M. 2020. Partial migration in a subtropical wading bird in the Southeastern United States. Ecosphere. 11(2):e03054. DOI: 10.1002/ecs2.3054<br><br>For the two particular individuals that moved to Mexico, see:<br>Picardi S, Borkhataria RR, Bryan AL, Jr, Frederick PC, Basille M. 2018. GPS telemetry reveals occasional dispersal of Wood Storks from the Southeastern US to Mexico. Caribbean Naturalist. Special Issue 2:23–29.	https://doi.org/10.5441/001/1.r0h6725k	CC_0
Movebank	1448409403	Vanellus vanellus	Jelle Loonstra	Jelle Loonstra		Movebank study 'Lapwing NFW Vanellus Vanellus'. Accessed on [date].	CC_BY_NC
Movebank	1480583194	Hydroprogne caspia	Patrik Byholm	Patrik Byholm	Byholm P, Beal M, Lötberg U, Åkesson S. 2022. Data from: Paternal transmission of migration knowledge in a long-distance bird migrant. Movebank Data Repository. https://www.doi.org/10.5441/001/1.352qf1cv <br>Byholm P, Beal M, Isaksson N, Lötberg U, Åkesson S. 2022. Paternal transmission of migration knowledge in a long-distance bird migrant. Nat Commun. https://doi.org/10.1038/s41467-022-29300-w	https://doi.org/10.5441/001/1.352qf1cv	CC_BY

Source	Study ID	Species	Principal Investigator	Contact person	Citation	Recommended reference	License type
Movebank	1531481854	Phaethon aethereus,Sula dactylatra,Sula leucogaster,Fregata aquila,Onychoprion fuscatus	Diane Elizabeth Baum	Diane Elizabeth Baum	Ascension Island Government and MPIAB	Movebank study 'ICARUS Ascension Island Government'. Accessed on [date].	CC_0
Movebank	1539583795	Cuculus canorus	Dutch Montagu's Harrier Foundation	Dutch Montagu's Harrier Foundation	MPIAB and Dutch Montagu's Harrier Foundation	Movebank study 'ICARUS Cuckoo Holland'. Accessed on [date].	CC_0
Movebank	1542155599	Gallinago gallinago,Cuculus canorus	Nyambayar Batbayar	Nyambayar Batbayar	Wildlife science and conservation center of Mongolia and MPIAB	Movebank study 'ICARUS Mongolia cuckoos Nymba'. Accessed on [date].	CC_0
Movebank	1542192442	Streptopelia turtur	JIGUET Frédéric	Jiguet Frédéric	MNHN and MPIAB	Movebank study 'ICARUS Turtle doves'. Accessed on [date].	CC_BY_NC
Movebank	1542206798	Cuculus canorus	JIGUET Frédéric	Jiguet Frédéric	MNHN and MPIAB	Movebank study 'ICARUS Cuckoo Fred Jiguet [ID_PROG 1189]'. Accessed on [date].	CC_BY_NC
Movebank	1543123177	Onychoprion fuscatus	Christopher John Feare	Martin Wikelski	Seychelles Governmen, Chris Feare, Rachel Bristol, Christine Larose and MPIAB	Movebank study 'ICARUS Seychelles Sooty terns'. Accessed on [date].	CC_0
Movebank	1562253659	Ciconia ciconia	Wolfgang Fiedler	Wolfgang Fiedler	Data from study "LifeTrack White Stork Sarralbe" in www.movebank.org by Max Planck Institute of Animal Behavior (Radolfzell, Germany) and Comune de Sarralbe	Movebank study 'LifeTrack White Stork Sarralbe [ID_PROG 1093]'. Accessed on [date].	CC_0
Movebank	1575973021	Hydroprogne caspia	Susanne Akesson	Susanne Akesson	Åkesson S, Lötberg U, Rueda-Uribe C. 2022. Data from: Study "Tracking of Caspian Terns (Hydroprogne caspia) in the Swedish Baltic Sea 2017-2020". Movebank Data Repository. https://www.doi.org/10.5441/001/1.hg1v55ct<br><br>Rueda-Uribe C, Lötberg U, Ericsson M, Tesson SVM, Åkesson S. 2021. First tracking of declining Caspian Terns (Hydroprogne caspia) breeding in the Baltic Sea reveals high migratory dispersion and disjunct annual ranges as obstacles to effective conservation. J Avian Biol. 52(9). https://doi.org/10.1111/jav.02743	https://doi.org/10.5441/001/1.hg1v55ct	CC_BY_NC

Source	Study ID	Species	Principal Investigator	Contact person	Citation	Recommended reference	License type
Movebank	1605024900	Chelydra serpentina,Branta canadensis,Anas platyrhynchos,Aix sponsa,Accipiter cooperii,Buteo jamaicensis,Buteo lineatus,Meleagris gallopavo,Bubo virginianus,Strix varia,Didelphis virginiana,Procyon lotor,Canis latrans	Stephen Blake	Stephen Blake		Movebank study 'Forest Park Living Lab'. Accessed on [date].	CC_0
Movebank	1605797471	Haematopus ostralegus	Bruno J. Ens	Peter Desmet	Dijkstra B, Dillerop R, Oosterbeek K, Bouten W, Desmet P, van der Kolk H, Ens BJ (2021) O_ASSEN - Eurasian oystercatchers (Haematopus ostralegus, Haematopodidae) breeding in Assen (the Netherlands). Dataset. https://doi.org/10.5281/zenodo.5653310	https://doi.org/10.5281/zenodo.5653310	CC_0
Movebank	1605798640	Haematopus ostralegus	Bruno J. Ens	Peter Desmet	Dokter AM, Oosterbeek K, Baptist M, Desmet P, van der Kolk H, Bouten W, Ens BJ (2021) O_BALGZAND - Eurasian oystercatchers (Haematopus ostralegus, Haematopodidae) wintering on Balgzand (the Netherlands). Dataset. https://doi.org/10.5281/zenodo.5653441	https://doi.org/10.3897/zookeys.1123.90623	CC_0
Movebank	1605799506	Haematopus ostralegus	Bruno J. Ens	Peter Desmet	Oosterbeek K, Bom R, Shamoun-Baranes J, Desmet P, van der Kolk H, Bouten W, Ens BJ (2021) O_SCHIERMONNIKOOG - Eurasian oystercatchers (Haematopus ostralegus, Haematopodidae) breeding on Schiermonnikoog (the Netherlands). Dataset. https://doi.org/10.5281/zenodo.5653477	https://doi.org/10.3897/zookeys.1123.90623	CC_0
Movebank	1605802367	Haematopus ostralegus	Bruno J. Ens	Henk-Jan van der Kolk	van der Kolk H, Oosterbeek K, Jongejans E, Frauendorf M, Allen AM, Bouten W, Desmet P, de Kroon H, Ens BJ, van de Pol M (2021) O_VLIELAND - Eurasian oystercatchers (Haematopus ostralegus, Haematopodidae) breeding and wintering on Vlieland (the Netherlands). Dataset. https://doi.org/10.5281/zenodo.5653890	https://doi.org/10.1111/ibi.13035	CC_0

Source	Study ID	Species	Principal Investigator	Contact person	Citation	Recommended reference	License type
Movebank	1605803389	Haematopus ostralegus	Bruno J. Ens	Peter Desmet	Oosterbeek K, de Jong J, Desmet P, van der Kolk H, Bouten W, Ens BJ (2021) O_AMELAND - Eurasian oystercatchers (Haematopus ostralegus, Haematopodidae) breeding on Ameland (the Netherlands). Dataset. https://doi.org/10.5281/zenodo.5647596	https://doi.org/10.3897/zookeys.1123.90623	CC_0
Movebank	1609400843	Ichthyaetus melanocephalus	Eric Stienen	Peter Desmet	Stienen EWM, Desmet P, Milotic T, Spanoghe G, Janssens K (2022) MEDGULL_ANTWERPEN - Mediterranean gulls (Ichthyaetus melanocephalus, Laridae) breeding near Antwerp (Belgium). Dataset. https://doi.org/10.5281/zenodo.6599272	https://doi.org/10.5281/zenodo.6599272	CC_0
Movebank	1623175929	Rupicapra rupicapra	Martin Wikelski	Martin Wikelski	MPIAB and Miriam Wiesner	Movebank study 'MPIAB Wiesner Chamois Conservation '. Accessed on [date].	CC_0
Movebank	1671751878	Struthio camelus	Dr Willem Burger	Willem Burger		Movebank study 'Tchad Redneck Ostrich'. Accessed on [date].	CC_BY_NC
Movebank	1701931040	Pernis apivorus	Wolfgang Fiedler	Wolfgang Fiedler		Movebank study 'Honey Buzzard Care Centre Release S Germany'. Accessed on [date].	CC_0
Movebank	1720694224	Canis latrans,Puma concolor	Julie Young	Peter Mahoney		Movebank study 'USU: Coyote and Puma (Fishlake NF, UT)'. Accessed on [date].	CC_0
Movebank	1724475305	Larus michahellis	Marcin	Marcin		Movebank study 'Yellow-legged Gulls'. Accessed on [date].	CC_0
Movebank	1767692280	Larus hyperboreus	Kyle Elliott	Allison Patterson	Baak JE, Patterson A, Gilchrist HG, Elliott KH. 2021. Data from: First evidence of diverging migration and overwintering strategies in glaucous gulls (Larus hyperboreus) from the Canadian Arctic. Movebank Data Repository. https://www.doi.org/10.5441/001/1.tj948m64 <br>Baak JE, Patterson A, Gilchrist HG, Elliott KH. 2021. First evidence of diverging migration and overwintering strategies in glaucous gulls (Larus hyperboreus) from the Canadian Arctic. Anim Migr. 8:98-109. https://doi.org/10.1515/ami-2020-0107	https://doi.org/10.5441/001/1.tj948m64	CC_BY
Movebank	1832666571	Caracal caracal	Laurel Serieys	Laurel Serieys	Serieys LEK, Bishop JM. 2024. Data from: Study "Caracal movement ecology study in Cape Town, South Africa". Movebank Data Repository. https://doi.org/10.5441/001/1.317 <br>These data are described in<br>Serieys LEK, Bishop JM, Rogan MS, Smith JA, Suraci JP, O'Riain MJ, Wilmers CC. 2023. Anthropogenic activities and age class mediate carnivore habitat selection in a human-dominated landscape. iScience. 26(7):107050. https://doi.org/10.1016/j.isci.2023.107050	https://doi.org/10.5441/001/1.317	CC_BY_NC

Source	Study ID	Species	Principal Investigator	Contact person	Citation	Recommended reference	License type
Movebank	1841091905	Numenius arquata	Geert Spanoghe	Peter Desmet	Spanoghe G, Janssens K, Nijs G, Milotic T, Desmet P (2021) CURLEW_VLAANDEREN - Eurasian curlews (Numenius arquata, Scolopacidae) breeding in Flanders (Belgium). Dataset. https://doi.org/10.5281/zenodo.5779130	https://doi.org/10.5281/zenodo.5779130	CC_0
Movebank	1841261165	Anas penelope	Jonas Waldenström	Marielle van Toor	van Toor ML, Kharitonov S, Švažas S, Dagys M, Kleyheeg E, Müskens G, Ottosson U, Žydelis R, Waldenström J. 2021. Migration distance affects how closely Eurasian wigeons follow spring phenology during migration. Mov Ecol. 9:61. doi: 10.1186/s40462-021-00296-0	https://doi.org/10.5441/001/1.dv5mm289	CC_BY
Movebank	1852945982	Pteropus melanotus	Christopher	Christopher	Todd CM, Westcott DA, Martin JM, Rose K, McKeown A, Hall J, Welbergen JA. 2022. Data from: Study "Movements of the Christmas Island flying fox, Australia". Movebank Data Repository. https://www.doi.org/10.5441/001/1.mn019k4d <br>These data are described in<br>Todd CM, Westcott DA, Martin JM, Rose K, McKeown A, Hall J, Welbergen JA. 2022. Body-size dependent foraging strategies in the Christmas Island flying-fox: implications for seed and pollen dispersal within a threatened island ecosystem. Mov Ecol. 10:19. https://doi.org/10.1186/s40462-022-00315-8	https://doi.org/10.5441/001/1.mn019k4d	CC_0
Movebank	1879445455	Chelonia mydas,Varanus salvator,Dendrocygna javanica,Anas platyrhynchos,Accipiter trivirgatus,Milvus migrans,Gyps himalayensis,Fulica atra,Numenius arquata,Tyto alba,Homo sapiens,Canis lupus,Bubo sumatranus,Nisaetus alboniger	Ratiwan Sitdhibutr	Martin Wikelski		Movebank study 'Poultry network Thailand 2022'. Accessed on [date].	CC_BY

Source	Study ID	Species	Principal Investigator	Contact person	Citation	Recommended reference	License type
Movebank	1904388965	Branta leucopsis,Anser albifrons,Anser anser	Bart Nolet	Nelleke Buitendijk	Buitendijk NH, de Jager M, Kruckenberg H, Kölzsch A, Moonen S, Müskens GJDM, Nolet BA. 2023. Data from: More grazing, more damage? Assessed damage to grassland relates non-linearly to goose grazing pressure. Movebank Data Repository. https://www.doi.org/10.5441/001/1.fk899541 <br>These data are described in<br>Buitendijk N, de Jager M, Hornman M, Kruckenberg H, Kölzsch A, Moonen S, Nolet B. 2022. More grazing, more damage? Assessed yield loss on agricultural grassland relates non-linearly to goose grazing pressure. J Appl Ecol. 59(12): 2878-2889. https://doi.org/10.1111/1365-2664.14306	https://doi.org/10.5441/001/1.fk899541	CC_BY
Movebank	1907973121	Tapirus terrestris,calibration	Patricia Medici	Michael Noonan	Medici EP. 2023. Data from: Study "Lowland tapirs, Tapirus terrestris, in Southern Brazil". Movebank Data Repository. https://www.doi.org/10.5441/001/1.03ck4s52 <br>These data are described in<br>Fleming CH, Deznabi I, Alavi S, Crofoot MC, Hirsch BT, Medici EP, Noonan MJ, Kays R, Fagan WF, Sheldon D, et al. 2022. Population-level inference for home-range areas. Methods Ecol Evol. 13(5):1027-1041. https://doi.org/10.1111/2041-210X.13815 <br>Medici EP, Mezzini S, Fleming CH, Calabrese JM, Noonan MJ. 2022. Movement ecology of vulnerable lowland tapirs between areas of varying human disturbance. Move Ecol. 10:14. https://doi.org/10.1186/s40462-022-00313-w	https://doi.org/10.5441/001/1.03ck4s52	CC_BY_NC
Movebank	1909487338	Anas platyrhynchos	Martin Wikelski	Martin Wikelski	MPIAB and FAO	Movebank study 'Poultry network China 2022'. Accessed on [date].	CC_0
Movebank	1941203363	Aquila rapax,Trigonoceps occipitalis,Terathopius ecaudatus	Andre Botha	Martin Wikelski	Vultures for Africa and MPIAB	Movebank study 'South Africa vultures VfA MPIAB'. Accessed on [date].	CC_0
Movebank	1958514115	Aquila chrysaetos	Anders Tøttrup	Signe Agermose Mathiasen Andersen		Movebank study 'The Danish Golden Eagle Project'. Accessed on [date].	CC_BY_NC
Movebank	2105214573	Branta canadensis	Manon Sorais	Manon Sorais	Sorais M., Patenaude-Monette M., Sharp C., Askren R., LaRocque A., Leblon B., and Giroux J.-F.	Movebank study 'North-East American Canada goose migration 2015-2021'. Accessed on [date].	CC_0
Movebank	2190520177	Larus michahellis	Marcin	Marcin	www.interrex-tracking.com	Movebank study 'Larus michahellis X Larus cachinnans hybrids'. Accessed on [date].	CC_0
Movebank	2272852276	Larus fuscus	Marcin	Marcin	www.interrex-tracking.com	Movebank study 'Gulls - Minsk'. Accessed on [date].	CC_BY_NC
Movebank	2277774459	Pelecanus occidentalis	Paul Leberg	Brock Geary		Movebank study 'Brown Pelican Leberg Gulf of Mexico 2019'. Accessed on [date].	CC_BY_NC

Source	Study ID	Species	Principal Investigator	Contact person	Citation	Recommended reference	License type
Movebank	2290252202	Branta leucopsis,Anser albifrons,Anser fabalis,Anser brachyrhynchus	Bart Nolet	Andrea Kölzsch	Kölzsch A, Lameris TK, Müskens GJDM, Schreven KHT, Buitendijk NH, Kruckenberg H, Moonen S, Heinicke T, Cao L, Madsen J, Wikelski M, Nolet BA. 2022. Data from: Wild goose chase: geese flee high and far, and with aftereffects from New Year's fireworks. Movebank Data Repository. https://www.doi.org/10.5441/001/1.g51fs0jv <br>These data are described in<br>Kölzsch A, Lameris TK, Müskens GJDM, Schreven KHT, Buitendijk NH, Kruckenberg H, Moonen S, Heinicke T, Cao L, Madsen J, et al. 2022. Wild goose chase: geese flee high and far, and with aftereffects from New Year's fireworks. Conserv Lett. e12927. https://doi.org/10.1111/conl.12927	https://doi.org/10.5441/001/1.g51fs0jv	CC_BY
Movebank	2298738353	Larus fuscus	Eric Stienen	Peter Desmet	Stienen EWM, Müller W, Lens L, Milotic T, Desmet P (2023) LBBG_ADULT - Lesser black-backed gulls (Larus fuscus, Laridae) breeding in Belgium. Dataset. https://doi.org/10.5281/zenodo.10055493	https://doi.org/10.5281/zenodo.10055493	CC_0
Movebank	2313947453	Platalea leucorodia	Geert Spanoghe	Peter Desmet	Spanoghe G, Janssens K, Milotic T, Desmet P (2023) SPOONBILL_VLAANDEREN - Eurasian spoonbills (Platalea leucorodia, Threskiornithidae) in Flanders (Belgium). Dataset. https://doi.org/10.5281/zenodo.10055132	https://doi.org/10.5281/zenodo.10055132	CC_0
Movebank	2379775078	Hypsignathus monstrosus	Elodie Schloesing	Elodie Schloesing	Schloesing E, Caron A, Chambon R, Courbin N, Labadie M, Nina R, Mouti Mdadinga F, Ngoubili W, Sandial D, N'Kaya-Tobi , Bourgarel M, De Nys HM, Cappelle J. 2024. Data from: Foraging and mating behaviors of Hypsignathus monstrosus at the bat-human interface in central African rainforest. Movebank Data Repository. https://doi.org/10.5441/001/1.278 <br>These data are described in<br>Schloesing E, Caron A, Chambon R, Courbin N, Labadie M, Nina R, Mouiti Mbadinga F, Ngoubili W, Sandiala D, N'Kaya Tobi, Bourgarel M, De Nys HM, Cappelle J. 2023. Foraging and mating behaviors of Hypsignathus monstrosus at the bat-human interface in central African rainforest. Ecol Evol. 13(7):e10240. https://doi.org/10.1002/ece3.10240	https://doi.org/10.5441/001/1.278	CC_0
Movebank	2398637362	Falco naumanni	Javier Bustamante	Javier Bustamante		Movebank study '(EBD) Lesser Kestrel (Falco naumanni) Senegal, MERCURIO-SUMHAL'. Accessed on [date].	CC_BY_NC
Movebank	2425475445	Gyps bengalensis,Gyps himalayensis	A B M Sarowar ALAM	A B M Sarowar ALAM		Movebank study 'Vulture Conservation Bangladesh'. Accessed on [date].	CC_0
Movebank	2526306516	Mergus merganser	Anthony Wetherhill	Anthony Wetherhill		Movebank study 'Satellite tracking of Goosander in Scotland'. Accessed on [date].	CC_BY_NC

Source	Study ID	Species	Principal Investigator	Contact person	Citation	Recommended reference	License type
Movebank	2608802883	Rangifer tarandus	Leif Egil Loe	Samantha P. H. Dwinnell	Loe, L. E., B. B. Hansen, A. Stien, S. D. Albon, R. Bischof, A. Carlsson, R. J. Irvine, M. Meland, I. M. Rivrud, E. Ropstad, V. Veiberg, and A. Mysterud. 2016. Behavioral buffering of extreme weather events in a high- Arctic herbivore. Ecosphere 7(6):e01374. 10.1002/ecs2.1374	https://doi.org/10.1002/ecs2.1374	CC_BY_NC
Movebank	2630711281	Morus bassanus	William A. Montevecchi	Kyle d'Entremont	d'Entremont KJN, Davoren GK, Montevecchi WA. 2023. Data from: Study "Northern Gannet Breeding Season GPS Data from Cape St. Mary's, NL, Canada: 2019 to 2022". Movebank Data Repository. https://www.doi.org/10.5441/001/1.5km7v2s3 <br>These data are described in<br>d'Entremont KJN, Davoren GK, Walsh CJ, Wilhelm SI, Montevecchi WA. 2022. Intra- and inter-annual shifts in foraging tactics by parental northern gannets Morus bassanus indicate changing prey fields. Mar Ecol Prog Ser. 698:155-170. https://www.doi.org/10.3354/meps14164	https://doi.org/10.5441/001/1.5km7v2s3	CC_BY_NC
Movebank	2636372210	Lynx rufus,Canis latrans	Laura Prugh	Laura Prugh	Prugh LR. 2023. Data from: Study "GPS tracking of bobcats and coyotes in northern Washington". Movebank Data Repository. https://www.doi.org/10.5441/001/1.gm93267b <br>These data are described in<br>Prugh LR, Cunningham CX, Windell RM, Kertson BN, Ganz TR, Walker SL, Wirsing AJ. 2023. Fear of large carnivores amplifies human-caused mortality for mesopredators. Science. 380(6646):754-758. https://doi.org/10.1126/science.adf2472 <br>Bassing SB, DeVivo M, Ganz TR, Kertson BN, Prugh LR, Roussin T, Satterfield L, Windell RM, Wirsing AJ, Gardner B. 2023. Are we telling the same story? Comparing inferences made from camera trap and telemetry data for wildlife monitoring. Ecol Appl. 33(1):e2745. https://doi.org/10.1002/eap.2745	https://doi.org/10.5441/001/1.gm93267b	CC_BY
Movebank	2658117564	Morus bassanus	Jana W E Jeglinski	Jana W E Jeglinski	Jeglinski JWE, Matthiopoulos J, Votier SC, Lane JV. 2024. Data from: Strong breeding colony fidelity in northern gannets following high pathogenicity avian influenza HPAIV outbreak. Movebank Data Repository. https://doi.org/10.5441/001/1.608 <br>These data are described in<br>Grémillet D, Ponchon A, Provost P, Gamble A, Abed-Zahar M, Bernard A, Courbin N, Delavaud G, Deniau A, Fort J, et al. 2023. Strong breeding colony fidelity in northern gannets following high pathogenicity avian influenza HPAIV outbreak. Biol Conserv. 286:110269. https://doi.org/10.1016/j.biocon.2023.110269<br><br>Jeglinski JWE, Lane JV, Votier SC, Furness RW, Hamer KC, McCafferty DJ, Nager RG, Sheddan M, Wanless S, Matthiopoulos J. 2024. HPAIV outbreak triggers short-term colony connectivity in a seabird metapopulation. Sci Rep. 14:3126. https://doi.org/10.1038/s41598-024-53550-x	https://doi.org/10.5441/001/1.608	CC_BY

Source	Study ID	Species	Principal Investigator	Contact person	Citation	Recommended reference	License type
Movebank	2658220054	Morus bassanus	Jana W E Jeglinski	Jana W E Jeglinski	Jeglinski JWE, Lane JV, Votier SC, Furness RW, Hamer KC, McCafferty DJ, Nager R, Sheddan M, Wanless S, Matthiopoulos J. 2024. Data from: HPAIV outbreak triggers short-term colony connectivity in a seabird metapopulation. Movebank Data Repository. https://doi.org/10.5441/001/1.607 <br>These data are described in<br>Jeglinski JWE, Lane JV, Votier SC, Furness RW, Hamer KC, McCafferty DJ, Nager RG, Sheddan M, Wanless S, Matthiopoulos J. 2024. HPAIV outbreak triggers short-term colony connectivity in a seabird metapopulation. Sci Rep. 14:3126. https://doi.org/10.1038/s41598-024-53550-x	https://doi.org/10.5441/001/1.607	CC_BY_NC
Movebank	2830157729	Nycticorax nycticorax	Mike Ward	Sarah Slayton		Movebank study 'Chicago BCNH Project'. Accessed on [date].	CC_0
Movebank	2915700754	Bubulcus ibis	Benjamin Vollot	Benjamin Vollot		Movebank study 'Bubulcus ibis_Vollot_ENVT_65_ID_PROG1035'. Accessed on [date].	CC_0
Movebank	2944153255	Larus smithsonianus	Mark Mallory	Sarah Gutowsky	Mallory ML, Craik S, Allard KA, Gutowsky S. 2023. Data from: Study "American Herring Gulls - GPS - Lobster Bay, Southwest Nova Scotia, Canada". Movebank Data Repository. https://www.doi.org/10.5441/001/1.292 <br>These data are described in<br>Gutowsky S, Baak JE, Craik S, Mallory M, Knutson N, d'Entremont A, Allard K. 2023. Seasonal and circadian patterns of herring gull (Larus smithsoniansus) movements reveal temporal shifts in industry and coastal island interaction. Ecol Solut Evid. 4(3):e12274. https://doi.org/10.1002/2688-8319.12274	https://doi.org/10.5441/001/1.292	CC_0
Movebank	2961927604	Falco naumanni	Javier Bustamante	Javier Bustamante	Lesser kestrel movements, EBD-CSIC, projects MERCURIO-SUMHAL	Movebank study '(EBD) Lesser Kestrel (Falco naumanni) Spain, MERCURIO-SUMHAL'. Accessed on [date].	CC_BY_NC
Movebank	2970193504	Falco tinnunculus	Javier Bustamante	Javier Bustamante	Common kestrel (Falco tinnunculus) movements, EBD-CSIC, projects MERCURIO & SUMHAL	Movebank study '(EBD) Common Kestrel (Falco tinnunculus) Spain, MERCURIO-SUMHAL'. Accessed on [date].	CC_BY_NC
Movebank	2976678046	Odocoileus virginianus	Blaise Ashley Newman	Blaise Ashley Newman	Newman BA, Dyal JR, Miller KV, Cherry MJ, D'Angelo GJ. 2023. Data from: Influence of visual perception on movement decisions by an ungulate prey species. Movebank Data Repository. https://www.doi.org/10.5441/001/1.293 <br>These data are described in<br>Newman BA, Dyal JR, Miller KV, Cherry MJ, D'Angelo GJ. 2023. Influence of visual perception on movement decisions by an ungulate prey species. Biol Open. 12(10):bio059932. https://doi.org/10.1242/bio.059932 <br>Dyal JR, Miller KV, Cherry MJ, D'Angelo GJ. 2022. White-tailed deer movement in response to helicopter surveys. Wildlife Soc Bull. 46(5):e1383. https://doi.org/10.1002/wsb.1383	https://doi.org/10.5441/001/1.293	CC_BY

Source	Study ID	Species	Principal Investigator	Contact person	Citation	Recommended reference	License type
Movebank	2984378217	Anthropoides virgo	Yuriy	Ivan Pokrovskiy	Andryushchenko Y, Pokrovsky I, Bernd V, Fiedler W, Wikelski M. 2024. Data from: Study "1000 Cranes. Ukraine.". Movebank Data Repository. https://doi.org/10.5441/001/1.590 <br>These data are described in<br>Yanco SW, Oliver RY, Iannarilli F, Carlson BS, Heine G, Mueller U, Richter N, Vorneweg B, Andryushchenko Y, Batbayar N, et al. 2024. Migratory birds modulate niche tradeoffs in rhythm with seasons and life history. Proc Natl Acad Sci USA. https://doi.org/10.1073/pnas.2316827121	https://doi.org/10.5441/001/1.590	CC_BY
Movebank	3094468724	Neophron percnopterus	Ron Efrat	Ron Efrat	Efrat R, Hatzofe O, Mueller T, Sapir N, Berger-Tal O. 2023. Data from: Early life and acquired experiences interact in shaping migratory and flight behaviors. Movebank Data Repository. https://www.doi.org/10.5441/001/1.298 <br>These data are described in<br>Efrat R, Hatzofe O, Mueller T, Sapir N, Berger-Tal O. 2023. Early life and acquired experiences interact in shaping migratory and flight behaviors. Curr Biol. https://doi.org/10.1016/j.cub.2023.11.012	https://doi.org/10.5441/001/1.298	CC_BY
Movebank	3131029654	Milvus milvus	Jan Skrabal	Jan Skrabal	Škrábal J, Literák I, Raab R. 2023. Study "Milvus_milvus_Soaring_over_Adriatic_sea". Movebank Data Repository. https://doi.org/10.5441/001/1.300 <br>These data are described in<br>Škrábal J, Krejčí Š, Raab R, Sebastián-González E, Literák I. 2023. Soaring over open waters: horizontal winds provide lift to soaring migrants in weak thermal conditions. Movement Ecol. 11:76. https://doi.org/10.1186/s40462-023-00438-6	https://doi.org/10.5441/001/1.300	CC_BY_NC
Movebank	3133580132	Lepus europaeus	Chloe tavernier	Chloe tavernier		Movebank study 'Lepus.europanus-Tavernier-SolarPark'. Accessed on [date].	CC_BY_NC
Movebank	3179890710	Vulpes vulpes	Tom Porteus	Tom Porteus	Porteus TA, Short MJ, Hoodless AN, Reynolds JC. 2024. Data from: Study "Red Fox (Vulpes vulpes) in UK wet grasslands". Movebank Data Repository. https://doi.org/10.5441/001/1.304 <br>These data are described in<br>Porteus TA, Short MJ, Hoodless AN, Reynolds JC. 2024. Movement ecology and minimum density estimates of red foxes in wet grassland habitats used by breeding wading birds. Eur J Wildlife Res. 70:8. https://doi.org/10.1007/s10344-023-01759-y	https://doi.org/10.5441/001/1.304	CC_0
Movebank	3186161092	Anatidae ,Dendrocygna bicolor	Manuel Olivier Grosselet	Manuel Olivier Grosselet		Movebank study 'Dendrocygna bicolor movement from Mexico City Manuel Grosselet'. Accessed on [date].	CC_0
Movebank	3218698288	Rynchops niger	Manuel Olivier Grosselet	Manuel Olivier Grosselet		Movebank study 'rynchops niger'. Accessed on [date].	CC_0

Source	Study ID	Species	Principal Investigator	Contact person	Citation	Recommended reference	License type
Movebank	3367931169	Nucifraga caryocatactes	Eike Lena Neuschulz	Eike Lena Neuschulz	Graf V, Sorensen MC, Mueller T, Neuschulz EL. 2024. Data from: Study "Movement patterns of seed dispersing spotted nutcrackers (Nucifraga caryocatactes)". Movebank Data Repository. https://doi.org/10.5441/001/1.324 <br>These data are described in<br>Sorensen MC, Mueller T, Donoso I, Graf V, Merges D, Vanoni M, Fiedler W, Neuschulz EL. Scatter-hoarding birds disperse seeds to sites unfavorable for plant regeneration. Mov Ecol. 10:38. https://doi.org/10.1186/s40462-022-00338-1 <br>Graf V, Mueller T, Grüebler MU, Kormann UG, Albrecht J, Hertel AG, Sorensen MC, Tschumi M, Neuschulz EL. In press, 2024. Individual behavior shapes patterns of bird-mediated seed dispersal. Funct Ecol.	https://doi.org/10.5441/001/1.324	CC_BY
Movebank	3413045568	Streptopelia turtur	JIGUET Frédéric	Andrea Kölzsch	HABITRACK: Habitat tracking for the conservation of huntable bird species	Movebank study 'Habitrack European Turtle Dove'. Accessed on [date].	CC_BY
Movebank	3578422072	Tyto furcata	Hermann Wagner	Hermann Wagner		Movebank study 'Galapagos barn owl project'. Accessed on [date].	CC_0
Movebank	3791354435	Panthera leo	Kevin MacFarlane	Kevin MacFarlane	MacFarlane, Kevin, 2014, The Ecology and Management of Kalahari lions in a Conflict Area in Central Botswana, PhD, Australian National University	https://doi.org/10.25911/5d78d54c1a50c	CC_0
Movebank	3809257699	Panthera leo	Kevin MacFarlane	Robert Heinsohn	MacFarlane K. 2014. The ecology and management of Kalahari lions in a conflict area in central Botswana [dissertation]. [Canberra (Australia)]: Australian National University. https://doi.org/10.25911/5d78d54c1a50c	https://doi.org/10.25911/5d78d54c1a50c	CC_BY
Movebank	3902656356	Nycticorax nycticorax	Mike Ward	Sarah Slayton		Movebank study 'Chicago BCNH Project (Druid)'. Accessed on [date].	CC_0
Movebank	3945977313	Aphriza virgata	Shawn Crimmins	Sam Simon		Movebank study 'Surfbird_UAF_BLM_InteriorAlaska'. Accessed on [date].	CC_0
Movebank	4447816442	Rissa tridactyla	Jacob Davies	Jacob Davies		Movebank study 'BTO - Whinnyfold 2021 - Kittiwake'. Accessed on [date].	CC_0
Movebank	4645303014	Vultur gryphus	Hannah Williams	Hannah Williams		Movebank study 'Juvenile Condor Cordoba Tatu Carreta Reserve'. Accessed on [date].	CC_0
Movebank	4725153649	Choloepus hoffmanni	Amelia Symeou	Amelia Symeou		Movebank study 'Choloepus hoffmanni, The Sloth Conservation Foundation, Puerto Viejo de Talamanca Costa Rica'. Accessed on [date].	CC_0
Movebank	4732684199	Bradypus variegatus	Rebecca Cliffe	Amelia Symeou		Movebank study 'Bradypus variegatus, The Sloth Conservation Foundation, Puerto Viejo de Talamanca Costa Rica'. Accessed on [date].	CC_0
Movebank	4739876185	Rallus ,Rallus elegans	Richard Temple Jr.	Richard Temple Jr.		Movebank study 'KIRA SW LA'. Accessed on [date].	CC_0

Source	Study ID	Species	Principal Investigator	Contact person	Citation	Recommended reference	License type
Movebank	4901146318	Connochaetes taurinus	Grant Hopcraft	Tom Morrison	Hopcraft, J.G.C., Morrison, T.A., and Stabach, J.A. (2024). Data from: Movement of resident wildebeest across the Masai Mara Ecosystem, Kenya. Movebank Data Repository.	Movebank study 'White-bearded wildebeest (Connochaetes taurinus) - Greater Mara Ecosystem (2017-2021)'. Accessed on [date].	CC_BY_NC
Movebank	5175345606	Lynx rufus,Urocyon cinereoargenteus	Laurel Serieys	Laurel Serieys	Serieys LEK, Matsushima SS, Wilmers CC. 2024. Data from: Study "Bobcat habitat connectivity study in central California". Movebank Data Repository. https://doi.org/https://doi.org/10.5441/001/1.323 <br>These data are described in<br>Serieys LEK, Rogan MS, Matsushima SS, Wilmers CC. 2021. Road-crossings, vegetative cover, land use and poisons interact to influence corridor effectiveness. Biol Conserv. 253:108930. https://doi.org/10.1016/j.biocon.2020.108930	https://doi.org/10.5441/001/1.323	CC_0
Tucker at al 2023		Alces alces	Bram Van Moorter				
Tucker at al 2023		Alces alces	Matthew Kauffman				
Tucker at al 2023		Antilocapra americana	Chris Geremia				
Tucker at al 2023		Antilocapra americana	Julie Young				
Tucker at al 2023		Antilocapra americana	Matthew Kauffman				
Tucker at al 2023		Canis aureus	Hubert Potocnik				
Tucker at al 2023		Canis latrans	David Drake				
Tucker at al 2023		Canis lupus	Daniel Stahler				
Tucker at al 2023		Canis lupus	Jerrold Belant				
Tucker at al 2023		Canis lupus	Mark Hebblewhite				
Tucker at al 2023		Capreolus capreolus	Francesca Cagnacci				
Tucker at al 2023		Cervus canadensis	Daniel Stahler				
Tucker at al 2023		Cervus canadensis	Julie Young				
Tucker at al 2023		Cervus canadensis	Kim Poole				
Tucker at al 2023		Cervus elaphus	Erling Meisingset				
Tucker at al 2023		Cervus elaphus	Johannes Signer				

Source	Study ID	Species	Principal Investigator	Contact person	Citation	Recommended reference	License type
Tucker at al 2023		Cervus elaphus	Marco Heurich				
Tucker at al 2023		Cervus elaphus	Peter Sunde				
Tucker at al 2023		Chrysocyon brachyurus	Rogerio Cunha de Paula				
Tucker at al 2023		Connochaetes taurinus	Grant Hopcraft				
Tucker at al 2023		Equus hemionus	Petra Kaczensky / Buuveibaatar Bayarbaatar				
Tucker at al 2023		Loxodonta africana	Morgan Hauptfleisch				
Tucker at al 2023		Loxodonta africana	Robert Pringle				
Tucker at al 2023		Odocoileus hemionus	Chris Geremia				
Tucker at al 2023		Odocoileus hemionus	Julie Young				
Tucker at al 2023		Odocoileus hemionus	Matthew Kauffman				
Tucker at al 2023		Odocoileus virginianus	Guillaume Bastille-Rousseau				
Tucker at al 2023		Odocoileus virginianus	Jerrold Belant				
Tucker at al 2023		Odocoileus virginianus	Vickie DeNicola				
Tucker at al 2023		Ovis canadensis	Chris Geremia				
Tucker at al 2023		Ovis canadensis	Kevin Monteith				
Tucker at al 2023		Ovis canadensis californiana	Julie Young				
Tucker at al 2023		Ovis canadensis canadensis	Julie Young				
Tucker at al 2023		Ovis canadensis nelsoni	Julie Young				
Tucker at al 2023		Panthera leo	Jacob Goheen				
Tucker at al 2023		Procapra gutturosa	Nandintsetseg Dejid				
Tucker at al 2023		Puma concolor	Christopher Wilmers				
Tucker at al 2023		Puma concolor	Julie Young				
Tucker at al 2023		Rangifer tarandus	Allicia Kelly				

Source	Study ID	Species	Principal Investigator	Contact person	Citation	Recommended reference	License type
Tucker at al 2023		Rangifer tarandus	Bram Van Moorter				
Tucker at al 2023		Tragelaphus angasii	Robert Pringle				
Tucker at al 2023		Tragelaphus strepsiceros	Robert Pringle				
Tucker at al 2023		Tragelaphus sylvaticus	Robert Pringle				
Tucker at al 2023		Ursus arctos	Jerrold Belant				
Tucker at al 2023		Ursus arctos	Jonas Kindberg				
Tucker at al 2023		Ursus arctos	Nuria Selva				
