# Supplementary material for: MoveTraits—A Database for Integrating Animal Behaviour Into Trait‐Based Ecology
Source: Ecol Lett. 2025 Dec 31;29(1):e70297. doi: 10.1111/ele.70297 (PMC12755192; doi:10.1111/ele.70297)
Supplement: Supplementary file 2 — Appendix S2: ele70297‐sup‐0002‐AppendixS2.pdf. [file ELE-29-0-s002.pdf]

MoveTraits.V0.1 Tutorial

Anne G. Hertel

8/20/2025

Contents

Download the database 1

Load MoveTraits database 1

Species level . . . 2

Individual level . . . 3

Within Individual level . . . 4

```r
library(tidyverse);library(bit64)
```

Download the database

Please download the three levels of the database from figshare and store in your local directory:

https://doi.org/10.6084/m9.figshare.28611890.v2

Mind - when you use the MoveTraits database, please always cite the database alongside the original paper:

Hertel, A., L. T. Beumer, R. Royaute, M. A. Tucker, J. Albrecht, R. S. Beltran, F. Cagnacci, S. Davidson, N. Dejid, R. Kays, A. Kölzsch, E. L. Neuschulz, K. Safi, A. K. Scharf, M. Schleuning, M. Wikelski, S. Åkesson, Y. Andryushchenko, M. Basille, G. Bastille-Rousseau, N. Batbayar, J. Belant, O. Berger-Tal, D. Berteaux, J. T. Bialas, J. Bishop, W. Boardman, R. Boone, A. Botha, M. Boyce, E. Buechley, P. Byholm, S. Chamaillé, P. Desmet, A. Droghini, Ł. Dylewski, R. Efrat, K. H. Elliott, A. Flack, M. Frederiksen, B. Geary, R. Heinsohn, M. Heurich, L. M. Irvine, Z. Jagiello, J. W. E. Jeglinski, F. Jiguet, M. Johnson, S. LaPoint, A. D. M. Latham, L. E. Loe, U. Lötberg, M. Mallory, S. Matsushima, E. Meisingset, C. Michelot, T. Milotic, R. Morato, A. Mysterud, R. Naniwadekar, S. Olson, F. Ossi, D. M. Palacios, S. Picardi, I. Pokrovsky, L. Prugh, N. Ranc, Y. Ropert-Coudert, D. Roshier, S. Rotics, N. Sapir, D. Schmidt-Rothmund, L. Serieys, S. Sherub, J. Signer, O. Spiegel, R. Temple, G. Tertitski, K. Thorup, M. Tobolka, A. P. Tøttrup, C. Wallace, L. Wilde, C. Wilmers and T. Mueller (2025). MoveTraits database, figshare. https://doi.org/10.6084/m9.figshare.28611890.v2

Beumer, L. T., A. G. Hertel, R. Royauté, M. A. Tucker, J. Albrecht, R. S. Beltran, F. Cagnacci, S. C. Davidson, N. Dejid, R. Kays, A. Kölzsch, A. Lohr, E. L. Neuschulz, K. Safi, A. K. Scharf, M. Schleuning, M. Wikelski and T. Mueller (2025). "MoveTraits – A database for integrating animal behaviour into trait-based ecology." bioRxiv. https://doi.org/10.1101/2025.03.15.643440

Load MoveTraits database

The MoveTraits database version 0.1 has been compiled from open access data from Tucker et al. 2023 "Behavioral responses of terrestrial mammals to COVID-19 lockdowns" (https://zenodo.org/records/7704108, file "Tucker_Road_Spatial.rds"; only data from 2019) and from all openly available GPS datasets in Movebank (effective 03.2025).

The database has three levels: species level trait summaries, individual level traits summaries, within individual repeated trait measures. The three levels are stored in separate files.

Species level

```r
MoveTrait.v0.1.sp <- readRDS("MoveTrait.v0.1_species.sum_20250311.rds")

dim(MoveTrait.v0.1.sp)
```

```
## [1] 163  83
```

The species level database consists of 83 columns

Table 1: Species level database column descriptions

ColumnName	Explanation
species	Species latin name
common_name	Species common name
class	mammal or bird
movement.mode	walk, fly, swim, arboreal
n1h	number of records contributing to the species summary for hourly displacement distance [m]
d1h.mean	species mean hourly displacement distance [m]
d1h.median	species median hourly displacement distance [m]
d1h.cv	species coefficient of. variation for hourly displacement distance [m]
d1h.95	species 95th percentile of hourly displacement distance [m]
d1h.05	species 5th percentile of hourly displacement distance [m]
n24h.days, d24h.mean, d24h.median, d24h.cv, d24h.95, d24h.05	species level summary of 24 hour displacement distance [m]
n.dmax24h.days, dmax24h.mean, dmax24h.median, dmax24h.cv, dmax24h.95, dmax24h.05	species level summary of maximum 24 hour displacement distance [m]
n.dmax7d.weeks, dmax7d.mean, dmax7d.median, dmax7d.cv, dmax7d.95, dmax7d.05	species level summary of maximum 7-day displacement distance [m]
n.max12m.years, dmax12m.mean, dmax12m.median, dmax12m.cv, dmax12m.95, dmax12m.05	species level summary of maximum 12-month displacement distance [m]
n.mcp24h.days, mcp24h.mean, mcp24h.median, mcp24h.cv, mcp24h.95, mcp24h.05	species level summary of daily range size / minimum convex polygon [m^2]
n.mcp7d.weeks, mcp7d.mean, mcp7d.median, mcp7d.cv, mcp7d.95, mcp7d.05	species level summary of weekly range size / minimum convex polygon [m^2]
n.mcp1m.months, mcp1m.mean, mcp1m.median, mcp1m.cv, mcp1m.95, mcp1m.05	species level summary of monthly range size / minimum convex polygon [m^2]
n.mcp12m.years, mcp12m.mean, mcp12m.median, mcp12m.cv, mcp12m.95, mcp12m.05	species level summary of annual range size / minimum convex polygon [m^2]
n.iou24h.days, iou24h.mean, iou24h.median, iou24h.cv, iou24h.95, iou24h.05	species level summary of daily intensity of use
n.iou1m.month, iou1m.mean, iou1m.median, iou1m.cv, iou1m.95, iou1m.05	species level summary of monthly intensity of use

ColumnName	Explanation
n.iou12m.year, iou12m.mean, iou12m.median, iou12m.cv, iou12m.95, iou12m.05	species level summary of annual intensity of use
n.di.days, di.mean, di.median, di.cv, di.95, di.05	species level summary of diurnaliuty index
contact_person_name	contact person name as stated on movebank or from tucker - multiple if species was tracked by multiple studies

You can use dplyr to query the dataframe. For example we can filter all arboreal species and pull their species name and mean daily displacement distance.

```
MoveTrait.v0.1.sp |>
  filter(movement.mode %in% c("arboreal")) |>
  dplyr::select(species, d24h.mean)
```

```
## # A tibble: 4 x 2
## # Groups:   species [4]
##   species              d24h.mean
##   <fct>                    <dbl>
## 1 Cebus capucinus          351.
## 2 Ateles geoffroyi         623.
## 3 Choloepus hoffmanni       20.1
## 4 Bradypus variegatus      414.
```

Or to tally how many bird and mammal species are contained

```
MoveTrait.v0.1.sp |> group_by(class) |>  tally()
```

```
## # A tibble: 2 x 2
##   class      n
##   <chr>  <int>
## 1 bird     108
## 2 mammal    55
```

Individual level

```
MoveTrait.v0.1.ind <- readRDS("MoveTrait.v0.1_individual.sum_20250311.rds")
MoveTrait.v0.1.ind$study_id <- as.integer64(MoveTrait.v0.1.ind$study_id)
```

The individual level database follows the same structure as the species level database. But contains more individual level information:

Table 2: Individual level database column descriptions

ColumnName	Explanation
study_id	Movebank study identifier
individual_id	Movebank individual identifier
species	Species latin name
common_name	Species common name
class	mammal or bird
movement.mode	walk, fly, swim, arboreal
sex	female - f, male - m, unknown - u
animal_mass	mass in grams

ColumnName	Explanation
animal_life_stage	estimated age in years or categorized as adult, subadult etc
source	Movebank or Tucker
mean.longitude	The individuals mean track longitude
mean.latitude	The individuals mean track latitude
median_timelag_mins	The tracks median fix interval in minutes
tracking_duration_days, tracking_start_date, tracking_end_date	the tracking duration, start and end date
contact_person_name	contact person name as stated on movebank
license_type	Movebank license
citation	ciattion as stated on movebank

We collected movement traits from a total of 107 bird and 56 mammal species represented by 6351 individuals (3646 birds, 2691 mammals). Data of 4560 individuals were collected from Movebank and 1777 from Tucker et al.

```
##      n
## 1 6351
```

```
## # A tibble: 2 x 2
##   class      n
##   <chr>  <int>
## 1 bird     108
## 2 mammal    55
```

```
## # A tibble: 2 x 2
##   class      n
##   <chr>  <int>
## 1 bird    3660
## 2 mammal  2691
```

```
## # A tibble: 2 x 2
##   source              n
##   <chr>           <int>
## 1 Tucker2023       1777
## 2 movebank.mar2025  4574
```

Within Individual level

```
MoveTrait.v0.1_rep.ind <- readRDS("MoveTrait.v0.1_withinindividual_20250311.rds")
```

The third level of the database provides repeated individual trait measures. It is structured in the following way - first we provide the individual level summaries, simialr to "MoveTrait.v0.1.ind", for each individual and every trait we provide the underlying repeated trait measures as a list of nested dataframes in columns 91 - 103

```
names(MoveTrait.v0.1_rep.ind[,91:103])
```

```
##  [1] "displ.1h"     "displ.24h"    "maxdispl.24h" "maxdispl.7d"  "maxdispl.12m"
##  [6] "mcp.24h"      "mcp.7d"       "mcp.1m"       "iou.24h"      "iou.1m"
## [11] "mcp.12m"      "iou.12m"      "diurnality"
```

```
class(MoveTrait.v0.1_rep.ind[,91])
```

```
## [1] "list"
```

For example for column 91 - “displ.1h” (hourly displacements), the underlying distribution for the first individual in the dataframe can be accessed in the following way:

```r
MoveTrait.v0.1_rep.ind[,91][[1]]
```

```
## # A tibble: 151 x 4
##    t_                     d1h    x_    y_
##    <dttm>               <dbl> <dbl> <dbl>
##  1 2014-07-20 15:08:00    33.3 -77.5  62.5
##  2 2014-07-20 15:56:00    42.0 -77.5  62.5
##  3 2014-07-20 16:45:00    48.0 -77.5  62.5
##  4 2014-07-20 17:33:00    83.4 -77.5  62.5
##  5 2014-07-21 03:20:00 24384.  -77.5  62.5
##  6 2014-07-21 04:06:00 21567.  -77.6  62.8
##  7 2014-07-21 04:52:00 20437.  -77.4  62.9
##  8 2014-07-21 06:56:00 31738.  -77.1  62.9
##  9 2014-07-21 12:15:00 88357.  -77.6  62.6
## 10 2014-07-21 14:22:00  5373.  -78.9  63.1
## # i 141 more rows
```

Which provides:

Table 3: Within individual level - hourly displacement column descriptions

ColumnName	Explanation
study_id	The starting time the step was taken
individual_id	The distance
species	Longitude
common_name	Latitude

We can unnest the repeated measures information for all individuals and one trait at a time in the following way:

First identify the trait of interest and the column names for this trait

```r
names(MoveTrait.v0.1_rep.ind[,91:103])
```

```
##  [1] "displ.1h"     "displ.24h"    "maxdispl.24h" "maxdispl.7d"  "maxdispl.12m"
##  [6] "mcp.24h"      "mcp.7d"       "mcp.1m"       "iou.24h"      "iou.1m"
## [11] "mcp.12m"      "iou.12m"      "diurnality"
```

```r
names(MoveTrait.v0.1_rep.ind |>
  dplyr::select(displ.24h) |>
  unnest(displ.24h))
```

```
## [1] "t_"   "d24h" "x_"   "y_"
```

Mind - because not all individuals have sufficient data for every trait to be estimated, these individuals have no data attached (NULL), for example the second individual for daily displacement:

```r
MoveTrait.v0.1_rep.ind[,92][[1]]
```

```
## NULL
```

To build a new dataframe containing individual level information (species, individual identity, mass etc) and within individual traits, we can select the columns of interest in the following way:

```r
d24h.rep.ind <-
  MoveTrait.v0.1_rep.ind |>
  unnest(displ.24h) |>
  dplyr::select("individual_id","species","common_name","animal_mass",
                "d24h","t_","x_","y_","contact_person_name","source")
```

```r
str(d24h.rep.ind)
```

```
## tibble [1,539,822 x 10] (S3: tbl_df/tbl/data.frame)
##  $ individual_id      : chr [1:1539822] "1008227508" "1008227508" "1008227508" "1008227508" ...
##  $ species            : chr [1:1539822] "Larus marinus" "Larus marinus" "Larus marinus" "Larus marinu
##  $ common_name        : chr [1:1539822] "great black-backed gull" "great black-backed gull" "great bl
##  $ animal_mass        : 'units' num [1:1539822] 1564 1564 1564 1564 1564 ...
##   ..- attr(*, "units")=List of 2
##   .. ..$ numerator  : chr "g"
##   .. ..$ denominator: chr(0)
##   .. ..- attr(*, "class")= chr "symbolic_units"
##  $ d24h               : num [1:1539822] 57 57.4 22.5 19.2 13.8 ...
##  $ t_                 : POSIXct[1:1539822], format: "2016-05-01 00:09:08" "2016-05-01 23:49:50" ...
##  $ x_                 : num [1:1539822] 8.47 8.47 8.47 8.47 8.47 ...
##  $ y_                 : num [1:1539822] 54.8 54.8 54.8 54.8 54.8 ...
##  $ contact_person_name: chr [1:1539822] "stefangarthe (Stefan Garthe)" "stefangarthe (Stefan Garthe)
##  $ source             : chr [1:1539822] "movebank.mar2025" "movebank.mar2025" "movebank.mar2025" "mo
```
